# Reaction hijacking inhibition of *Plasmodium falciparum* asparagine tRNA synthetase

**DOI:** 10.21203/rs.3.rs-3198291/v1

**Published:** 2023-07-27

**Authors:** Stanley C. Xie, Yinuo Wang, Craig J. Morton, Riley D. Metcalfe, Con Dogovski, Charisse Flerida A. Pasaje, Elyse Dunn, Madeline R Luth, Krittikorn Kumpornsin, Eva S Istvan, Joon Sung Park, Kate J. Fairhurst, Nutpakal Ketprasit, Tomas Yeo, Okan Yildirim, Mathamsanqa N. Bhebhe, Dana M. Klug, Peter J. Rutledge, Luiz C. Godoy, Sumanta Dey, Mariana Laureano De Souza, Jair L. Siqueira-Neto, Yawei Du, Tanya Puhalovich, Mona Amini, Gerry Shami, Duangkamon Loesbanluechai, Shuai Nie, Nicholas Williamson, Gouranga P. Jana, Bikash C. Maity, Patrick Thomson, Thomas Foley, Derek S. Tan, Jacquin C Niles, Byung Woo Han, Daniel E Goldberg, Jeremy Burrows, David A. Fidock, Marcus C.S. Lee, Elizabeth A. Winzeler, Michael D. W. Griffin, Matthew H. Todd, Leann Tilley

**Affiliations:** 1 Department of Biochemistry and Pharmacology, Bio21 Molecular Science and Biotechnology Institute, The University of Melbourne, Melbourne, VIC 3010, Australia; 2 School of Pharmacy, University College London, London WC1N 1AX, United Kingdom; 3 Biomedical Manufacturing Program, CSIRO, Clayton South, Australia; 4 Center for Structural Biology, Center for Cancer Research, National Cancer Institute, Frederick, MD 21702, USA; 5 Department of Biological Engineering, Massachusetts Institute of Technology, Cambridge, MA 02139, USA; 6 Department of Pediatrics, School of Medicine, University of California, San Diego, La Jolla, California 92093, USA; 7 Parasites and Microbes Programme, Wellcome Sanger Institute, Hinxton, CB10 1SA, United Kingdom; 8 Calibr, Division of the Scripps Research Institute, La Jolla, CA 92037, USA; 9 Division of Infectious Diseases, Department of Medicine, Washington University in St. Louis, USA; 10 Research Institute of Pharmaceutical Sciences & Natural Products Research Institute, College of Pharmacy, Seoul National University, Seoul 08826, Republic of Korea; 11 Center for Malaria Therapeutics and Antimicrobial Resistance, Columbia University Medical Center, New York, NY 10032, USA; 12 Department of Microbiology and Immunology, Columbia University Medical Center, New York, NY 10032, USA; 13 Chemical Biology Program, Sloan Kettering Institute, Memorial Sloan Kettering Cancer Center, New York, NY 10065, USA; 14 School of Chemistry, University of Sydney, Camperdown, NSW 2006, Australia; 15 Melbourne Mass Spectrometry and Proteomics Facility, Bio21 Molecular Science and Biotechnology Institute, The University of Melbourne, Melbourne, VIC 3010, Australia; 16 TCG Lifesciences Private Limited, Salt-lake Electronics Complex, Kolkata, India; 17 School of Chemistry, The University of Edinburgh, Edinburgh EH9 3JJ, United Kingdom; 18 Medicines for Malaria Venture, 20, Route de Pré-Bois 1215, Geneva 15, Switzerland; 19 Division of Infectious Diseases, Department of Medicine, Columbia University Medical Center, New York, NY 10032, USA; 20 Wellcome Centre for Anti-Infectives Research, Biological Chemistry and Drug Discovery, University of Dundee, Dundee DD1 4HN, United Kingdom; 21 Structural Genomics Consortium, University College London, London WC1N 1AX, United Kingdom

## Abstract

Malaria poses an enormous threat to human health. With ever increasing resistance to currently deployed drugs, breakthrough compounds with novel mechanisms of action are urgently needed. Here, we explore pyrimidine-based sulfonamides as a new low molecular weight inhibitor class with drug-like physical parameters and a synthetically accessible scaffold. We show that the exemplar, OSM-S-106, has potent activity against parasite cultures, low mammalian cell toxicity and low propensity for resistance development. *In vitro* evolution of resistance using a slow ramp-up approach pointed to the *Plasmodium falciparum* cytoplasmic asparaginyl tRNA synthetase (*Pf*AsnRS) as the target, consistent with our finding that OSM-S-106 inhibits protein translation and activates the amino acid starvation response. Targeted mass spectrometry confirms that OSM-S-106 is a pro-inhibitor and that inhibition of *Pf*AsnRS occurs via enzyme-mediated production of an Asn-OSM-S-106 adduct. Human AsnRS is much less susceptible to this reaction hijacking mechanism. X-ray crystallographic studies of human AsnRS in complex with inhibitor adducts and docking of pro-inhibitors into a model of Asn-tRNA-bound *Pf*AsnRS provide insights into the structure activity relationship and the selectivity mechanism.

## Introduction

Malaria is a devastating disease. In 2021, *Plasmodium falciparum*, the most deadly of the malaria species, affected more than 200 million people and caused more than 600,000 deaths, mostly of African children^1^. Disruptions to funding and services due to the COVID-19 pandemic exacerbated the problems caused by widespread resistance of parasites to currently used therapies^2^, as well as resistance of the mosquito vectors to pyrethroid insecticides^3^. In particular, the recent emergence in Africa of artemisinin resistance-conferring K13 mutations^4, 5^ is of great concern. There is an urgent need to develop new antimalarial compounds with novel mechanisms of action.

A recent study showed that some *P. falciparum* aminoacyl-tRNA synthetases (aaRSs) are susceptible to reaction hijacking by nucleoside sulfamates^6^. Tight-binding nucleoside sulfamate-amino acid adducts are generated in the active site, thereby blocking enzyme activity. A *Plasmodium*-specific reagent, ML901, was identified that hijacks *P. falciparum* tyrosine RS (*Pf*TyrRS). By contrast, *Homo sapiens* Tyr-RS (*Hs*TyrRS) does not catalyse formation of the adduct. X-ray crystallography revealed that differential flexibility of a loop over the catalytic site may underpin differential susceptibility to reaction-hijacking by ML901 ^6^.

**Fig. 1.**
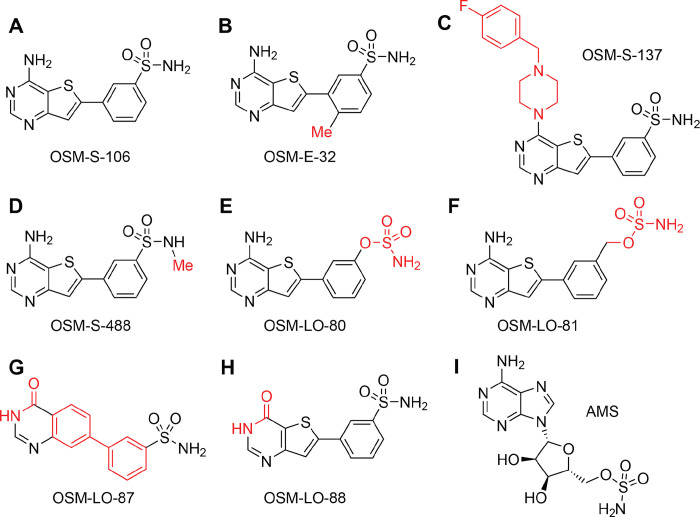
Structures of OSM-S-106, OSM-S-106 derivatives and adenosine 5’-sulfamate. (**A**) OSM-S-106. (**B**) OSM-E-32. (**C**) OSM-S-137. (**D**) OSM-S-488. (**E**) OSM-LO-80. (**F**) OSM-LO-81. (**G**) OSM-LO-87. (**H**) OSM-LO-88. (**I**) AMS. Structural differences between OSM-S-106 and derivatives (**B**-**H**) are highlighted in red.

Here, we explored a new chemical class of reaction hijacking inhibitors. OSM-S-106 (Fig. 1A) is an aminothieno pyrimidine benzene sulfonamide, with activity against *P. falciparum* cultures. OSM-S-106 was first identified as part of a screen of compounds from a GSK library (originally tagged as TCMDC-135294, ^7^). While OSM-S-106 is structurally divergent from the nucleoside sulfamates previously shown to target *Pf*aaRSs via the reaction hijacking mechanism, mass spectrometry-based identification of covalent adducts and biochemical analyses revealed that Asn-tRNA-bound *Pf*AsnRS is indeed susceptible to attack by OSM-S-106. By contrast, *Hs*AsnRS is much less susceptible to hijacking by OSM-S-106.

AsnRSs are class II aaRSs, characterised by an α/β fold, with a highly conserved active site. We solved, for the first time, the crystal structure of *Hs*AsnRS in complex with the natural intermediate, Asn-AMP, as well as with synthetically generated Asn-OSM-S-106, providing insights into ligand-induced changes in the enzyme structure. We generated an AlphaFold model of the *Pf*AsnRS dimer. *Pf*AsnRS harbours a large insert adjacent to motif I that is predicted to affect the dynamics of binding of substrates and release of products from the active site. This *Plasmodium*-specific structural feature may underpin differences in susceptibility to reaction hijacking. We generated a molecular model of *Pf*AsnRS in complex with the native product, tRNA-Asn; and docked OSM-S-106 and derivatives into the AMP-binding site. This analysis provided insights into the structure-activity relationships that underpin the potency of OSM-S-106.

## Results

### Selection of OSM-S-106

A previous screen of 2 million GSK Public Limited Company (PLC) compounds against *P. falciparum* cultures yielded the Tres Cantos Antimalarial Set (TCAMS) library with 13,500 active compounds^7^. One of these compounds, OSM-S-106 (TCMDC-135294; Fig. 1A), was considered attractive from a medicinal chemistry perspective due to its ligand efficient structure, its synthetically accessible scaffold and its drug-like properties (Table S1). Consequently, OSM-S-106 was chosen as the subject of an Open Source Malaria campaign^8, 9^.

### Synthesis and characterisation of OSM-S-106 and derivatives

**Scheme 1.**
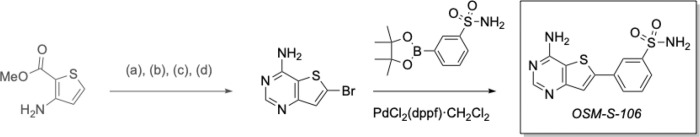
Aminothieno pyrimidine sulfamate synthesis protocol.

Synthesis of OSM-S-106 and its derivatives was achieved using the aminothieno pyrimidine synthesis protocol, as delineated in the Supplementary Material. Briefly, the pyrimidine cores for the OSM-S-106 were prepared using a two-step heterocycle synthesis, followed by bromination and amination and finally a Suzuki coupling with benzenesulfonamide pinacol boronate (Scheme 1), to yield OSM-S-106 in yields ranging from 45% to 70%. The synthetic sequence was conducted on a gram scale, giving 200 mg or more of the final product. The molecule appears stable in the solid state with respect to degradation under ambient conditions (Supplementary Material and Supplementary Dataset 1).

### OSM-S-106 exhibits selective activity against Plasmodium blood and liver stages and low intrinsic clearance by human microsomes

We confirmed that OSM-S-106 exhibits good activity against the 3D7 line of *P. falciparum* (50% inhibitory concentration (IC_50_72h_) = 0.058 ± 0.017 μM; n = 8). Importantly, OSM-S-106 also prevented the development of *P. berghei* in liver cells (HepG2-A16-CD81-EGF; IC_50_ = 0.25/0.42 μM; n = 2, Table 1). OSM-S-106 exhibited low cytotoxicity against the HepG2 cell line (IC_50_ = 49.6/47.3 μM; n = 2), indicating a selectivity index (IC_50_^HepG2^/ IC_50_^PbLuc^) of over 140-fold (Table 1). OSM-S-106 is stable during incubation with human microsomes (t_1/2_ = 395/619 min; n = 2); but shows rapid intrinsic clearance in mouse microsomes (t_1/2_ = 19.7/20.4 min; n = 2) (Table S2).

### SAR analysis reveals compound features that are needed for potent activity

Several substitutions of the aminothienopyrimidine core, the pendant aromatic ring and the primary sulfonamide were prepared, as described in the Supplementary Information, to establish structure activity relationships (SAR), with a readout of activity against 3D7 cultures. The addition of a methyl group to the thiophene ring (OSM-E-32; Fig. 1B), with the aim of reducing compound planarity, was not tolerated (Table 1), nor was the addition of a methyl group to the primary sulfonamide (OSM-S-488; Fig. 1D). A compound bearing a larger amine substituent on the pyrimidine ring (OSM-S-137, Fig 1C) showed low (though not zero) activity (IC_50_ = 4.4 ± 2.6 μM). Conversion of the sulfonamide to a sulfamate (OSM-LO-80; Fig. 1E; 2.1 μM) or a sulfamate with an extended linker (OSM-LO-81; Fig. 1F; 0.9 μM) decreased activity (Table 1).

An hydroxyquinazolinyl benzene sulfonamide, MMV026546, was also identified in the TCAMS library. Here we resynthesised this pyridone sulfonamide (renamed OSM-LO-87, Fig. 1G); and also generated the corresponding oxo-thienopyrimidinyl benzene sulfonamide (OSM-LO-88, Fig. 1H). OSM-LO-87 exhibited no antimalarial potency, suggesting that the initial report was a false positive; while OSM-LO-88 exhibited very low activity 18.7 ± 1.8 μM (Table 1). Given the marked sensitivity of OSM-S-106 to substitution, we continued the characterisation of the initial hit, and sought to identify the target to better understand the requirements for activity.

### OSM-S-106 exhibits a low propensity for developing resistance.

*In vitro* evolution and whole genome sequencing has been used extensively to explore *P. falciparum*’s propensity for developing resistance and to identify novel antimalarial drug targets and resistance mechanisms^10, 11^. A single-step selection was set up, using 10^7^ Dd2-B2 parasites in each well of a 24-well plate, with OSM-S-106 at a concentration of 3 x IC_90_ (508 nM). No recrudescent parasites were observed over a 60-day selection period.

To validate our protocol, a reference selection was run in parallel using 2×10^5^ Dd2-B2 parasites in each well of a 96-well plate with a *Plasmodium*-specific dihydroorotate dehydrogenase inhibitor (DSM265; ^12^) at 5 x IC_50_ (58 nM). This yielded 14 recrudescent wells (corresponding to a Minimum Inoculum for Resistance (MIR) of 1.4 × 10^6^), with evidence of separate amplification events encompassing the *dhodh* locus consistent with increased IC_50_ values (Supplementary Table 3,4; Supplementary Dataset 2). Based on these studies, we conclude that the MIR value for OSM-S-106 is > 2.4 × 10^8^.

### *Parasites selected against OSM-S-106 in a slow ramp-up method acquire mutations in asparaginyl tRNA synthetase (Pf*AsnRS*) and nucleoside transporter 4 (PfNT4)*

We employed a gradual ramp-up exposure method, which has been reported to increase the success of evolving resistant parasites^13^. Starting at the IC_50_72h_ concentration, we gradually increased to four times the IC_50_72h_ concentration over two months in a Dd2 genetic background (10^9^ parasites). This selection yielded 21 newly emerged coding variants in 10 unique core genes (Table 2; Supplementary Table 5; Supplementary Datasets 3,4). Sequencing six clones (2 from flask 3 and 4 from flask 2) and comparing SNVs/Indels and CNVs present in the clones relative to their isogenic parents identified two candidate genes of interest. Parasites from both flasks contained a missense mutation (S22C or H320L) in PF3D7_0103200, which encodes *P. falciparum* nucleoside transporter 4 (*Pf*NT4). In addition, parasites from both flasks contained mutations in the PF3D7_0211800 locus, which encodes *P. falciparum* cytoplasmic asparaginyl tRNA synthetase (*Pf*AsnRS). All four Dd2-OSM-2 clones harboured an R487S change in *Pf*AsnRS, while both Dd2-OSM-3 clones and one of the Dd2-OSM-2 clones had a Copy Number Variant (CNV) across a genomic segment on chromosome 2 that contains *Pf*AsnRS. This CNV has never been reported before nor have SNVs in *PfAsnRS*. Of interest, the precise boundaries of the CNV region varied between clones, suggesting independent events. The likelihood of missense mutations and CNVs in the same gene by chance is extremely low. Based on our experience with hundreds of selections we hypothesized that *Pf*NT4 is more likely a drug resistance gene and that *Pf*AsnRS is the target.

### OSM-S-106 inhibits protein translation and induces the amino acid starvation response

**Fig. 2.**
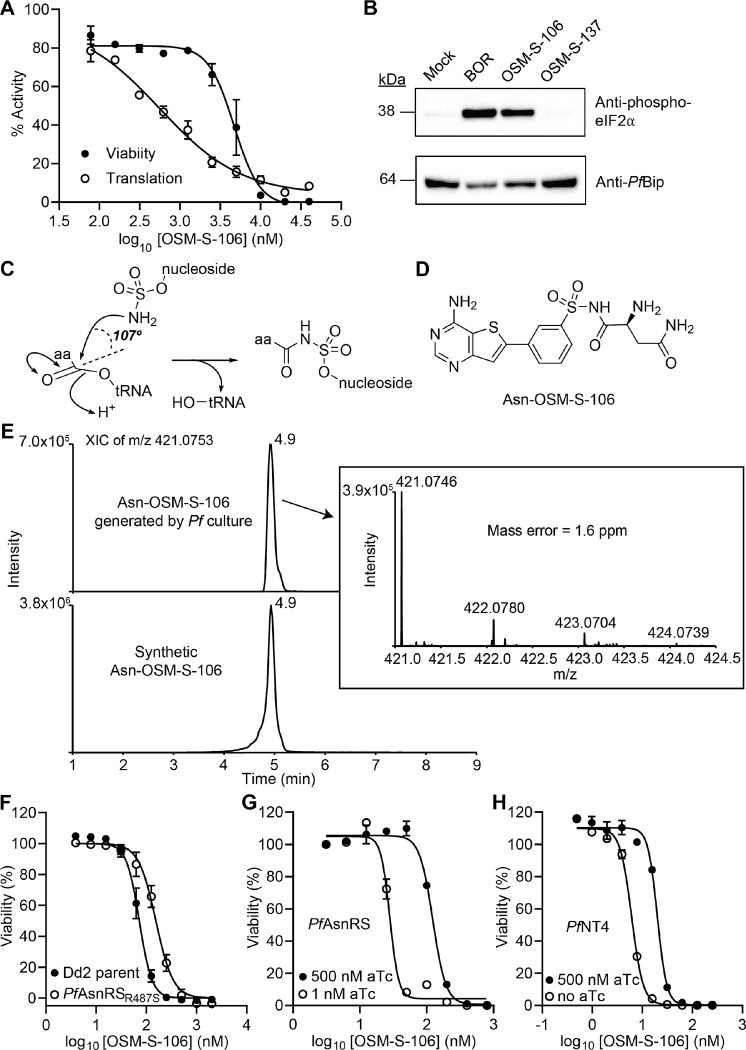
Identification of the *P. falciparum* target of OSM-S-106 (**A**) *P. falciparum* cultures (Cam3.II-rev; trophozoite stage; 30–35 h p.i.) were exposed to OSM-S-106 for 6 h. Protein translation was assessed in the last two hours of the incubation, via the incorporation of OPP. Aliquots of inhibitor-exposed cultures were washed and returned to cultures, and viability was estimated at the trophozoite stage of the next cycle. IC_50_ (Translation) = 0.51 μM, IC_50_ (Viability) = 4.7 μM. Error bars correspond to SEM of three independent experiments. (**B**) Trophozoite stage Cam3.II_rev parasites (30–35 h p.i.) were incubated with 0.05% DMSO (Mock), 50 nM borrelidin (BOR) or 2.5 μM OSM-S-106 or 2.5 μM OSM-S-137 for 3 h. Western blots of lysates were probed for phosphorylated eIF2α with *Pf*BiP as a loading control. Additional blots are presented in Supplementary Fig. 1C. (**C**) Schematic showing aaRS-catalysed attack of a nucleoside sulfamate on an activated amino acid to form an amino acid adduct. (**D**) Structure of Asn-OSM-S-106. (**E**) *P. falciparum*-infected RBCs were treated with 10 μM OSM-S-106 for 3 h. Extracts were subjected to LCMS. The extracted ion chromatograms of the Asn-OSM-S-106 adduct generated by *P. falciparum* (upper panel) and the synthetic conjugate at m/z 421.0753 (lower panel). The inset shows MS analysis of the parasite-generated Asn-OSM-S-106 adduct. (**F**) Sensitivity to OSM-S-106 exposure (72-h) for a cloned wildtype line (Dd2) and a CRISPR-edited clone harbouring *Pf*AsnRS_R487S_. Data represent 5 replicates and error bars correspond to SD. See Supplementary Table 6 for data values. (**G,H**) Sensitivity to OSM-S-106 exposure (72-h) for aptamer-regulatable *Pf*AsnRS (**G**) and *Pf*NT4 (**H**) lines upon addition of aTc (closed circles) and with the target expression reduced (open circles), with data normalized to a no drug control. Data represent the mean of three replicates and error bars correspond to SD. See Supplementary Table 7 for data values.

To investigate *Pf*AsnRS as a potential target, we examined the ability of OSM-S-106 to inhibit protein translation. We employed an in-cell assay of protein translation in *P. falciparum* trophozoites, monitored by incorporation of a clickable derivative of the puromycin homologue, O-propargyl-puromycin (OPP)^14, 15^. Following a 6-h exposure, protein translation is inhibited with an IC_50_ value of 0.51 μM (Fig. 2A), consistent with *Pf*AsnRS being the target. The same pulsed exposure to OSM-S-106 leads to loss of viability in the next cycle; albeit with a higher IC_50_ value (4.7 μM; Fig. 2A). The data are consistent with previous reports showing that even short-term inhibition of aaRSs is lethal^6, 15^. As a control, we showed that pulsed (6 h) exposure to WR99210, an inhibitor of *PfDHFR*, prevented replication into the next cycle, but had no short-term effect on protein translation (Supplementary Fig. 1A). Exposure to cycloheximide, which inhibits protein translation by interfering with the ribosome, also inhibited OPP incorporation; however, a 6-h exposure had no effect on viability (Supplementary Fig. 1B).

Inhibition of aminoacyl tRNA synthetases leads to a build-up of uncharged tRNA, which in turn leads to eIF2α phosphorylation^16, 17^. OSM-S-106 exposure triggers eIF2α phosphorylation, to a similar extent as the known threonyl-tRNA synthetase inhibitor, borrelidin (Fig. 2B; Supplementary Fig. 1C). OSM-S-137, a derivative of OSM-S-106, with lower activity (Fig. 1C), has no effect under the same exposure conditions (Fig. 2B; Supplementary Fig. 1C). Taken together, the data are consistent with OSM-S-106 targeting *Pf*AsnRS.

### OSM-S-106 hijacks the catalytic activity of P. falciparum aminoacyl tRNA synthetases

The identification of *Pf*AsnRS as a potential target of OSM-S-106 was of particular interest to our team given that the compound bears a primary sulfonamide attached to an aromatic ring structure, reminiscent of nucleoside sulfamates, such as ML901 and adenosine 5’-*O*-sulfamate (AMS), that have been shown to be pro-inhibitors of aaRSs^6^ (Fig. 2C). We therefore considered the possibility that OSM-S-106 might exert its activity against *Pf*AsnRS via a reaction hijacking mechanism. Such a mechanism would be expected to generate an Asn-OSM-S-106 conjugate (Fig. 2D). We treated *P. falciparum* cultures with 1 μM or 10 μM OSM-S-106 for 3 h and used targeted mass spectrometry to search for the 20 possible amino acid conjugates. Extracts were subjected to liquid chromatography-coupled with mass spectrometry (LC-MS) and the anticipated masses were interrogated. The extract yielded a strong signal for Asn-OSM-S-106, with a precursor ion at m/z 421.0746, retention time at 4.9 min and fragmentation spectrum consistent with that of the synthetic Asn-OSM-S-106 conjugate (Fig. 2E; Supplementary Fig. 2A). In the samples with 10 μM OSM-S-106 treatment, minor MS peaks were also detected for the adducts of glycine and alanine along with MS/MS spectra containing characteristic OSM-S-106 ion at m/z 307 (Supplementary Fig. 2B–E), suggesting that these GlyRS and AlaRS are also weakly susceptible to inhibition via the reaction hijacking mechanism.

### Further characterisation of OSM-S-106 targets

To investigate *Pf*AsnRS as a target, transfectants harbouring the *Pf*AsnRS_R487S_ mutation were generated in a Dd2 parent line (Supplementary Fig. 3A–C). The mutant line exhibited a 2.3-fold decreased sensitivity to OSM-S-106 (Fig. 2F; Supplementary Table 6), consistent with *Pf*AsnRS being an important target.

We used the TetR/DOZI-RNA aptamer module to conditionally regulate the expression of some of the *P. falciparum* gene products in which mutations arose during evolution of resistance (see Table 2), namely *Pf*AsnRS, *Pf*GDH3 and *Pf*NT4. We also modulated the level of cytoplasmic *Pf*AlaRS (PF3D7_1367700) and *Pf*GlyRS (PF3D7_1420400), given that we observed production of a low level of adducts of OSM-S-106 with these amino acids. OSM-S-106 contains a sulfonamide group that is predicted to bind tightly to carbonic anhydrase^18^, suggesting *P. falciparum* carbonic anhydrase (*Pf*CA; PF3D7_1140000) as another possible target, so, we also knocked down *Pf*CA. Luminescence-based viability assays revealed that knockdown of the cytoplasmic aaRSs perturbs the growth of parasites (Supplementary Fig. 3D), indicating the genes are essential for blood stage development. By contrast, knockdown of *Pf*NT4, *Pf*GDH3 and *Pf*CA did not have a significant impact on parasite growth, consistent with previous studies for *Pf*NT4 and *Pf*GDH3 ^19, 20^.

Upon knockdown of *Pf*AsnRS, the parasites exhibited a 6.6-fold enhancement in susceptibility to OSM-S-106 compared to the control (Fig. 2G; Supplementary Table 7), confirming the inhibitory interaction. *Pf*NT4 knockdown also sensitized the parasites to OSM-S-106 (3-fold shift; Fig. 2H; Supplementary Table 7). Differential sensitivity to OSM-S-106 was not observed following knockdown of *Pf*AlaRS, *Pf*GlyRS, *Pf*GDH3, *Pf*CA (Supplementary Fig. 3E–H; Supplementary Table 7), arguing against these proteins being important targets of the inhibitor.

### Recombinant HsAsnRS has very limited capacity to generate Asn-OSM-S-106 adducts compared to PfAsnRS

Using an *E. coli* expression system, we generated recombinant *Pf*AsnRS and *Hs*AsnRS. Following removal of the His-tag and generation of wildtype enzymes, analytical ultracentrifugation revealed that the proteins are dimeric in solution (Supplementary Fig. 4). We used targeted mass spectrometry to examine the ability of recombinant *Pf*AsnRS and *Hs*AsnRS to generate the Asn-OSM-S-106 conjugate. The enzymes were incubated with ATP, Asn and *E. coli* tRNA in the presence of OSM-S-106 (10 μM). Following precipitation of the tRNA and protein, the supernatants were subjected to LC-MS analysis. For *Pf*AsnRS, we detected a peak at m/z 421.0735 with retention time at 7.1 min, consistent with that of the Asn-OSM-S-106 standard (Supplementary Fig. 5A). The identity of the adduct was further confirmed by MS/MS analysis compared with the synthetic standard (Supplementary Fig. 5B). By contrast, a signal with 18-fold lower intensity was detected for Asn-OSM-S-106 when *Hs*AsnRS was incubated with OSM-S-106 under the same conditions (Supplementary Fig. 5C).

### OSM-S-106 inhibits ATP consumption by PfAsnRS but not HsAsnRS

**Fig. 3.**
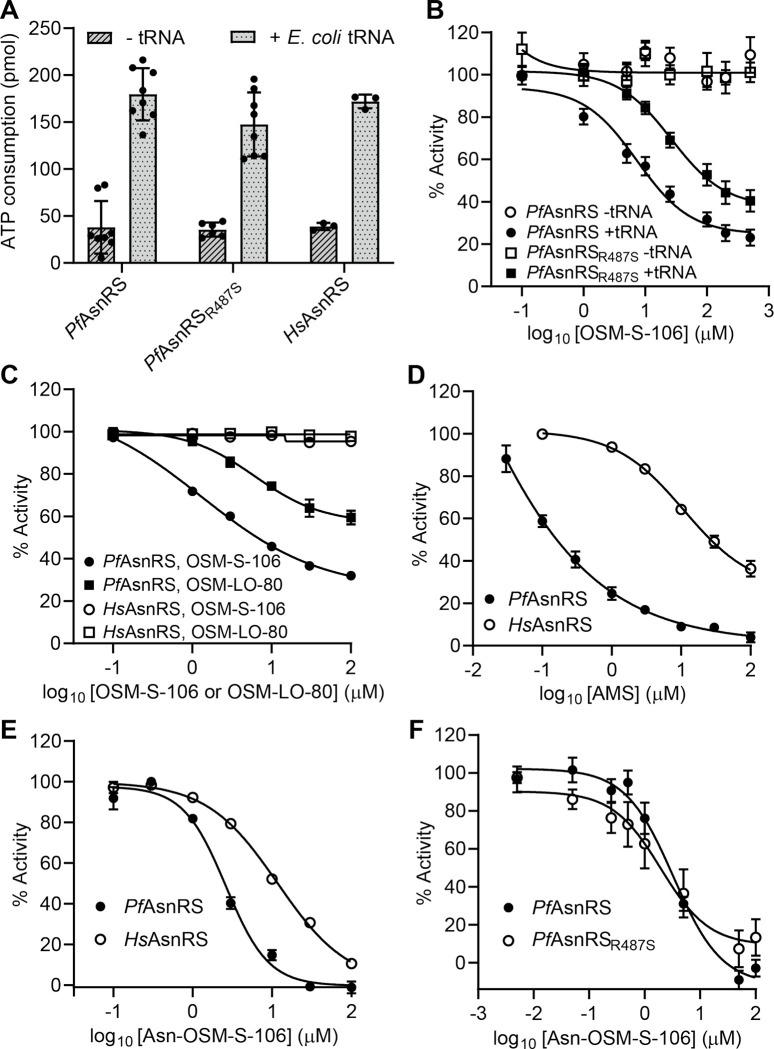
OSM-S-106 hijacks *Pf*AsnRS enzyme activity but is less effective against *Pf*AsnRS_R487_ and *Hs*AsnRS. (**A**) ATP consumption by wildtype *Pf*AsnRS, *Pf*AsnRS_R487S_ and full-length *Hs*AsnRS in the presence and absence of *E. coli* tRNA. ATP is consumed during the formation (and release) of AMP-Asn in the initial phase of the aminoacylation reaction. Reactions were incubated at 37°C for 1 h. *Pf*AsnRS and *Pf*AsnRS_R487S_: 0.5 μM; *Hs*AsnRS: 0.2 μM. Data represent the average of at least three independent assays and error bars correspond to SD. (**B**) Effects of increasing concentrations of OSM-S-106 on ATP consumption at 37°C, over a period of 2.5 h, by wildtype *Pf*AsnRS and *Pf*AsnRS_R487S_ in the presence or absence of *E. coli* tRNA. Enzyme concentration = 0.5 μM. IC_50_ values: Plus *E. coli* tRNA = 7.3 μM for *Pf*AsnRS and 26.3 μM for *Pf*AsnRS_R487S_; minus *E. coli* tRNA > 500 μM. Data represent the average of at least three independent assays. Error bars correspond to SEM. (**C**) Effects of increasing concentrations of OSM-S-106 and OSM-LO-80 on ATP consumption by *Pf*AsnRS and *Hs*AsnRS. Reactions were incubated at 37°C for 1 h with 0.05 μM *Pf*AsnRS or 0.2 μM *Hs*AsnRS in the presence of *E. coli* tRNA. IC_50_ values for OSM-S-106: 3.6 μM for *Pf*AsnRS and >100 μM for *Hs*AsnRS. IC_50_ values for OSM-LO-80: >100 μM for *Pf*AsnRS and *Hs*AsnRS. Data are the average of at least three independent experiments. Error bars represent SEM. (**D**) Effects of AMS on ATP consumption by *Pf*AsnRS and *Hs*AsnRS. Reactions were incubated at 37°C for 1 h with increasing concentrations of AMS and 0.05 μM *Pf*AsnRS or 0.2 μM *Hs*AsnRS. IC_50_ values: 3.7 nM for *Pf*AsnRS; 25 μM for *Hs*AsnRS. Data represent the average of three independent experiments and error bars correspond to SEM. (**E,F**) Effects of synthetic Asn-OSM-S-106 on ATP consumption by *Pf*AsnRS and *Hs*AsnRS (**E**) and *Pf*AsnRS and *Pf*AsnRS_R487S_ (**F**). Reactions were incubated at 37°C for 1 or 2.5 h with increasing concentrations of Asn-OSM-S-106, and 0.5 μM of enzymes without tRNA. IC_50_ values: 2.6 / 3.3 μM for *Pf*AsnRS; 12 μM for *Hs*AsnRS, 1.9 μM for *Pf*AsnRS_R487S_. Data points represent the average of at least three independent experiments and error bars correspond to SEM. The component concentrations for all above reactions are ATP (10 μM), asparagine (200 μM), pyrophosphatase (1 unit/mL) and *E. coli* tRNA (2.5 mg/mL), if present.

We assessed the ability of the recombinant aaRSs to consume ATP in the initial phase of the aminoacylation reaction, *i.e.*, via the formation and release of AMP. For these studies, we used recombinant versions of *Pf*AsnRS and *Pf*AsnRS_R487S_, the mutant selected during evolution of resistance to OSM-S-106, as well as *Hs*AsnRS. In the absence of tRNA, *Pf*AsnRS, *Pf*AsnRS_R487S_ and *Hs*AsnRS consume low levels of ATP (Fig. 3A). Addition of *E. coli* tRNA substantively increases the level of ATP consumption (Fig. 3A), consistent with productive aminoacylation.

OSM-S-106 inhibits consumption of ATP by wildtype *Pf*AsnRS when added in the presence of tRNA, but not in its absence (Fig. 3B). This is consistent with a reaction hijacking mechanism whereby enzyme-bound amino acid-conjugated tRNA is the target of nucleophilic attack by OSM-S-106 (Fig. 2C). The *Pf*AsnRS_R487S_ mutant is inhibited less efficiently than wildtype *Pf*AsnRS (Fig. 3B), consistent with the decreased sensitivity of cultures of *Pf*AsnRS_R487S_ mutants to OSM-S-106 (Supplementary Table 6). OSM-LO-80, which exhibits weaker antimalarial potency (Table 1) also shows weaker inhibition of the consumption of ATP by *Pf*AsnRS (Fig. 3C). OSM-S-106 and OSM-LO-80 do not inhibit consumption of ATP by *Hs*AsnRS (Fig. 3C).

We previously showed that AMS (Fig. 1I) is a broadly reactive pro-inhibitor that hijacks a range of aaRSs in both *Plasmodium* and human cell lines^6^. Here we used AMS, generated as previously described (Mujumdar et al., 2018), and kindly provided by Dr Steven Langston, Takeda Pharmaceuticals, as a positive control for reaction-hijacking inhibition of aaRSs. When added in the presence of all substrates, AMS inhibits ATP consumption by both *Pf*AsnRS and *Hs*AsnRS; although *Pf*AsnRS appears to be more susceptible (Fig. 3D). These data suggest that *Hs*AsnRS is intrinsically less susceptible to reaction hijacking; and that OSM-S-106 has structural features that exploit that difference in susceptibility, providing selectivity.

Synthetically generated Asn-OSM-S-106 strongly inhibits the activity of *Pf*AsnRS, *Pf*AsnRS_R487S_ and, to a lesser extent, *Hs*AsnRS (Fig. 3 E,F), suggesting that the susceptibility to reaction hijacking depends largely on the ability of the enzyme to generate the Asn-OSM-S-106 adduct, rather than the ability to bind the preformed conjugate, as previously observed for ML901 hijacking of *Pf*TyrRS^6^.

### *Structures of Hs*AsnRS *reveal loop stabilisation when Asn-AMP is generated in the enzyme active site*

Type II aaRSs typically comprise an N-terminal β-barrel anticodon-binding domain connected via a hinge region to a larger C-terminal catalytic domain that adopts a α–β fold, with three motifs (I-III) involved in ATP binding and dimerization, and a linker domain between motifs II and III ^21^ (Supplementary Fig. 6). Members of our team previously published a structure of the apo form of the N-terminally truncated *Hs*AsnRS, known as the canonical domain (CD) (PDB: 6A0E; ^22^). Here, we generated recombinant CD*Hs*AsnRS and verified that it forms a dimer in solution (Supplementary Fig. 4D,H). We solved the apo structure at a resolution of 1.9 Å, confirming the expected conformation (Supplementary Fig. 7).

**Fig. 4.**
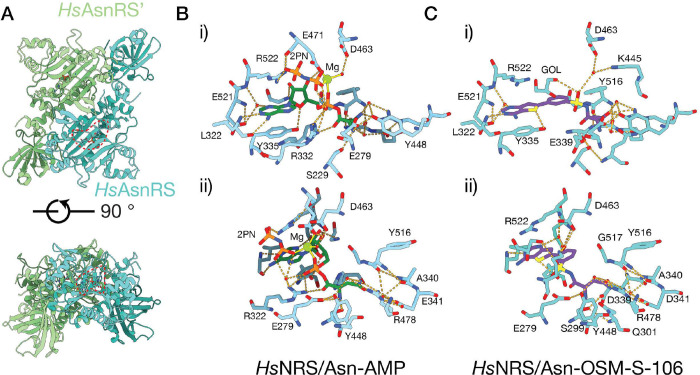
Structures of the Asn-AMP/CD*Hs*AsnRS and OSM-S-106/CD*Hs*AsnRS complexes. (**A**) Structure of the CD*Hs*AsnRS dimer in complex with Asn-AMP. The bound Asn-AMP is circled (dotted red lines) and the two chains of the dimer are coloured differently. (**B**) Key inhibitor contact residues in the Asn-AMP/CD*Hs*AsnRS complex. Hydrogen bonds are indicated by yellow dashed lines. (**C**) Key inhibitor contact residues in the Asn-OSM-S-106/CD*Hs*AsnRS complex. Hydrogen bonds are indicated by yellow dashed lines. Abbreviations: 2PN – imidodiphosphoric acid, GOL – glycerol.

Following initial unsuccessful attempts to generate crystals in the presence of ATP and Asn, CD*Hs*AsnRS was incubated in the presence of Asn and the ATP analogue, AMPPNP. Diffraction quality crystals were obtained; and we solved the structure (refined at 2.2 Å resolution), revealing the presence of Asn-AMP in the active site (Figure 4A,B; Supplementary Fig. 8). Interactions with the adenylate and asparagine moieties stabilize the activated adenylate in the characteristic bent conformation, with the plane of the ribose angled ~90° relative to the adenine ring system, as previously observed in other AsnRSs and indeed other class II synthetases^23, 24^.

Comparison of the Asn-AMP-bound CD*Hs*AsnRS and our apo CD*Hs*AsnRS structures reveals local changes in and around the active site (Supplementary Fig. 7B,8B). Of particular interest is residue E279, which lies just N-terminal of the beta hairpin (K286 – F295), within a loop that is not well defined in the apo CD*Hs*AsnRS electron density, indicating flexibility. Upon binding of Asn-AMP, the side chain of E279 interacts with the Asn-AMP asparagine moiety, leading to stabilisation of the loop. Similarly, upon binding of Asn-AMP, R322 in Motif II interacts with the Asn-AMP phosphate and E224 interacts with the adenylate part of the ligand, leading to stabilisation of residues Q325 - R329 within a larger loop (Y321 – E334) that lies between the two beta strands of Motif II (Figure 4B; Supplementary Fig. 8B,C). Stabilization of these loops may increase the affinity of binding of the activated intermediate, allowing sufficient residence time for reaction with the cognate tRNA. The conserved “flipping” loop (E279 - T283; Supplementary Fig. 6) has previously been shown to undergo dynamic motions that facilitate tRNA binding^25^.

### *Structures of Hs*AsnRS *in complex with Asn-OSM-S-106 and Asn-AMS*

As described above, *Hs*AsnRS is very inefficient in catalysing the formation of Asn-OSM-S-106. However, we were successful in crystalising CD*Hs*AsnRS in complex with synthetic Asn-OSM-S-106, refined to 2.0 Å resolution (Fig 4C; Supplementary Fig. 9). OSM-S-106 is located in the adenylate binding pocket; however, in contrast to the ribose of the adenylate-containing structures, the substituted benzene ring of OSM-S-106 is planar with respect to the thienopyrimidine ring system, which positions the amino acid moiety in the correct pose to bind in the same pocket occupied by the asparagine of Asn-AMP (Fig. 4B). Stabilisation of the flexible loop structures adjacent to the active site is mediated by side chain interactions of E279 with the asparagine of Asn-OSM-S-106, and interactions of R322 and E224 with the sulfonamide and thienopyrimidine group, respectively (Supplementary Figs. 9B,C).

We also solved the structure of CD*Hs*AsnRS in complex with synthetic Asn-AMS, refined to 1.9 Å (Supplementary Fig. 10), revealing sodium in the position occupied by magnesium in our Asn-AMP bound structure. Asn-AMS makes similar interactions with the active site to those observed in the Asn-AMP complex, including the nature of the interactions stabilising the loops around the active site (Supplementary Fig. 10B,C).

### *Sequence alignment and an AlphaFold model of the Pf*AsnRS *structure reveal a Plasmodium-specific insert*

Alignment of the *Pf*AsnRS sequence with sequences from a range of species reveals moderate to good conservation (Supplementary Fig. 6). One *Plasmodium*-specific feature of interest is a low complexity insert, adjacent to the flipping loop (Supplementary Fig. 6). In *P. falciparum*, the inset has a length of 76 amino acids^26^.

**Fig. 5.**
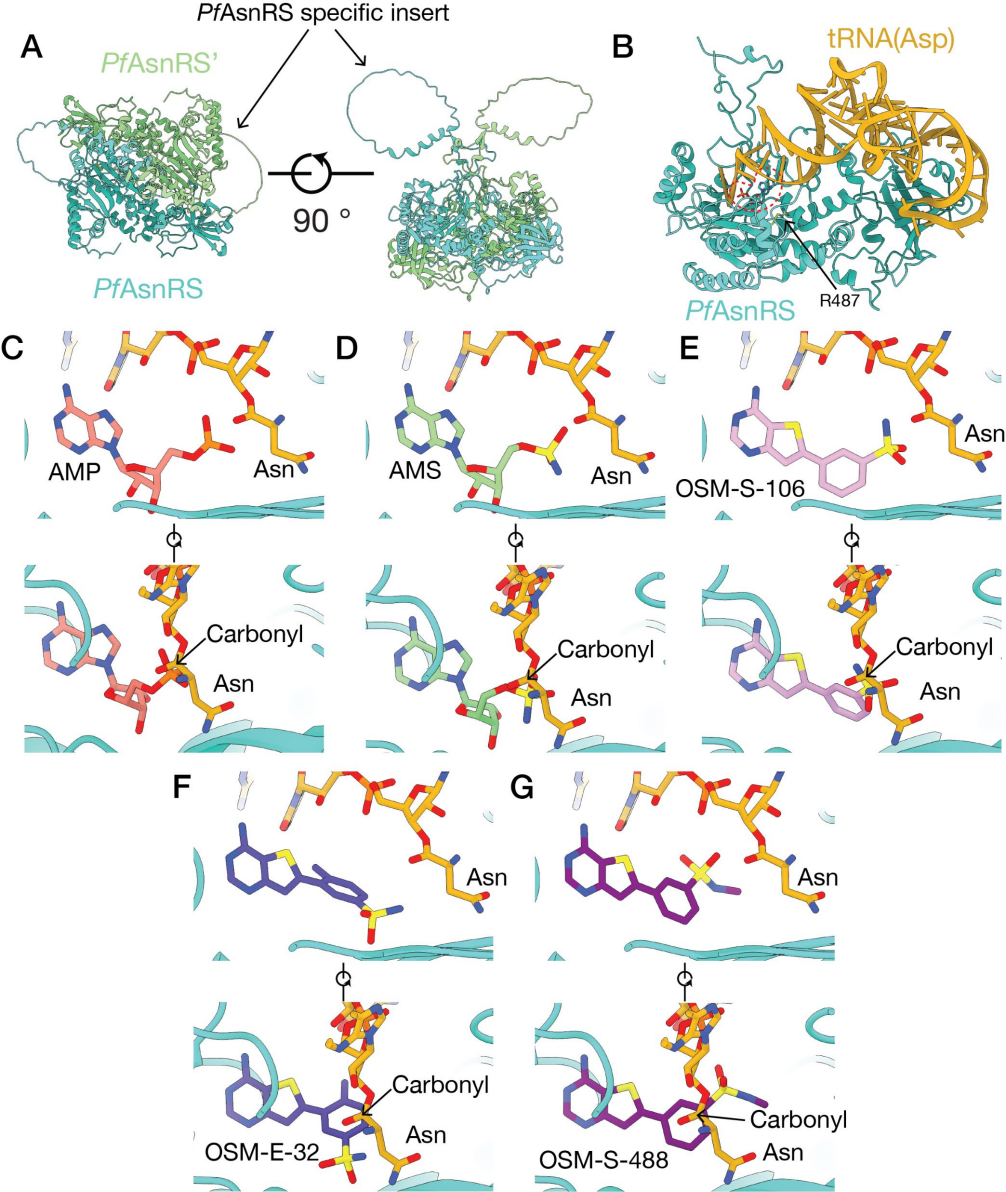
A model of the *Pf*AsnRS-Asn-tRNA complex and compound docking reveal mechanisms for differential compound activity. (**A**) AlphaFold-Multimer model of the *Pf*AsnRS dimer. Each chain of the dimer, and the long, disordered *Pf*AsnRS-specific insert are depicted. (**B**) Model of the *Pf*AsnRS-Asn-tRNA complex, generated by overlay of the *Pf*AsnRS model with the *E. coli* AspRS/Asp-tRNA complex (PDB ID 1C0A, ^28^). The position of the bound ligand is highlighted with a dotted red line. Residue R487 (arrowed) lies close to the tRNA binding site. (**C**-**G**) Representative *in silico* docks of compounds to the *Pf*AsnRS/Asn-tRNA model for (**C**) AMP, (**D**) AMS, (**E**) OSM-S-106 (see also Supplementary Fig. 11C), (**F**) OSM-E-32, and (**G**) OSM-S-488. Two orientations of each docked compound are shown to illustrate alignment of the reactive groups with the tRNA-Asn carbonyl carbon. The binding poses of AMP, AMS and OSM-S-106 are similar to the corresponding parts of our experimentally determined structures of the Asn-AMP/CD*Hs*AsnRS, Asn-AMS/CD*Hs*AsnRS, and Asn-OSM-S-106/CD*Hs*AsnRS complexes. The AMS sulfamate and the OSM-S-106 sulfonamide are in a suitable position to attack the carbonyl carbon of Asn-tRNA.

Our attempts to generate a high-resolution crystal structure of *Pf*AsnRS were not successful. We therefore generated a molecular model of the *Pf*AsnRS dimer (Fig. 5A) using AlphaFold Multimer ^27^. The model exhibits the anticipated N-terminal β-barrel anticodon-binding domain connected to a larger C-terminal catalytic domain that adopts an α–β fold. The large loop insert is modelled as a partly structured domain that extends from a beta hairpin turn. An overlay of the *Pf*AsnRS model with the *Hs*AsnRS structure shows that the long insert interrupts the beta hairpin turn in *Hs*AsnRS (Supplementary Figure 11A). While the insert domain structure is not well-defined, it appears to occupy an area that extends over the active site cavity (Fig. 5A), in a position that could influence the dynamics of the aminoacylation reaction.

### *Generation of a Pf*AsnRS*-tRNA(Asn) structural model*

Our previous studies provided evidence that reaction hijacking involves nucleophilic attack of an aromatic sulfate/sulfonamide on the amino acid charged tRNA product in the enzyme active site. For this reaction to occur, the pro-inhibitor needs to bind into the AMP vacated site. We therefore generated a model of the Asn-tRNA-bound *Pf*AsnRS complex (Fig. 5B) to enable docking of different OSM-S-106 derivatives into the AMP binding site, in the context of the bound Asn-tRNA product. PDB entries for different class II RS enzymes complexed with tRNA were inspected and the *E. coli* Asp-RS enzyme complexed with aspartyl-AMP and the cognate *E. coli* tRNA (PDB entry 1C0A)^28^, was chosen as a suitable template.

Superimposition of 1C0A onto the AlphaFold model for the *Pf*AsnRS shows that the residues around the active site pocket are closely aligned, except that in the *Pf*AsnRS model the terminal CAA of the tRNA acceptor stem clashes with residues in the flipping loop, a region of the protein known to reposition to allow acceptor stem access on tRNA binding ^28^. Close alignment of residues lining the active site of *Hs*AsnRS and our model provides further confidence in the active site structure of our *Pf*AsnRS model (Supplementary Figure 11B). The flipping loop residues from 1C0A, which are in the open, acceptor stem-binding conformation were copied into the *Pf*AsnRS model and then manually modified to the correct *Pf*AsnRS sequence. The tRNA from 1C0A was copied into the *Pf*AsnRS model without modification. The AMP-Asn bond was broken and Asn was connected manually to the 3’OH oxygen of the tRNA A76 (Fig. 5B) with AMP remaining in the binding pocket. The modelled complex was then minimised to correct geometry and remove any steric clashes generated during modelling using SybylX2.1. Of interest, the residue (R487S) that is modified in *P. falciparum* upon selection for resistance, lies in a helix that partially caps the active site (Fig. 5B) and may interact with the tRNA backbone to stabilise the complex.

### *Docking pro-inhibitors into the Pf*AsnRS*-tRNA-Asn model provides a basis for understanding SAR*

Susceptibility to reaction hijacking depends on the ability of the enzyme to generate the Asn adduct, rather than the ability to bind the preformed conjugate. Understanding the series SAR therefore requires assessment of suitable, low-energy poses of bound pro-inhibitors for reaction with the tRNA-Asn carbonyl carbon, i.e. a suitable distance and angle^29^ between the reacting centres.

We first docked AMP and the high potency AMS pro-inhibitor into the AMP binding site using Surflex in SybylX2.1 (Figures 5C,D). AMS adopts a very similar position to AMP. Given that there is free rotation around the carbon-sulfur bond, the sulfamate nitrogen can be well positioned to attack the target carbonyl. Importantly, the docking poses of these compounds are similar to the positions of the corresponding components of ligands observed in our structures of the Asn-AMP/*Hs*AsnRS, Asn-AMS/*Hs*AsnRS complexes. While the AMS pro-inhibitor is a useful positive control compound, its efficient targeting of *Hs*AsnRS makes it a poor starting point for antimalarial drug development. Thus, OSM-S-106 remains of greater interest because of that compound’s selectivity.

OSM-S-106 was docked into the AMP pocket of the *Pf*AsnRS-tRNA-Asn complex and adopts a similar conformation in the *Pf*AsnRS model to that observed for the Asn-OSM-S-106/CD*Hs*AsnRS crystal structure, with the aryl ring twisted toward co-planarity with the thienopyrimidine ring system (Fig. 5E). The model reveals that the sulfonamide nitrogen of OSM-S-106 overlaps with the AMP phosphate and AMS sulfamate, while the thienopyrimidine of OSM-S-106 occupies a similar position to the adenine groups of AMP and AMS. The top scoring docks show rotation of the sulfonamide around the carbon-sulfur bond (Supplementary Figure 11C), allowing positioning of the OSM-S-106 sulfonamide nitrogen in a good orientation for attack on the tRNA-Asn carbonyl carbon, leading to inhibitor formation.

We compared the docking poses for key OSM-S-106 derivatives. OSM-E-32 has a methyl substitution on the aryl ring, introduced to increase the dihedral angle between the aromatic rings for the purpose of improving solubility. In the lowest energy docked conformation for this compound (Fig. 5F), this ring is twisted relative to the thienopyrimidine ring. Rotation to coplanarity, and adoption of better geometry for the hijacking reaction, would introduce a steric clash between the methyl group and an arginine residue (R584). This inability to adopt a suitable geometry may underpin the decreased antimalarial potency. OSM-S-488, which has a methyl substituent on the sulfonamide, docks with a pose where the sulfonamide nitrogen is positioned well away from the target carbonyl because the extra methyl group cannot easily be accommodated (Fig. 5G); this poor positioning may underlie its poor activity.

OSM-LO-81 and OSM-LO-80 are sulfamate derivatives of OSM-S-106, adopting the reactive moiety of ML901, with and without an additional carbon in the alkyl linker. In both cases, small changes in distances and geometry arising from steric clashes or inferior low-energy poses appear to equate to large changes in reaction rate (Supplementary Figures 11D,E).

Similarly, the hydroxyquinazolinyl benzene sulfonamide, OSM-LO-87, exhibited no antimalarial potency. The lowest energy docked conformation for the lowest energy tautomer of OSM-LO-87 shows a substantive shift of the hydroxyquinazolinyl moiety compared with the position for the corresponding thienopyrimidine group of OSM-S-106 (Supplementary Fig. 11F), while for the best docked conformations of the other tautomers, the sulfonamide nitrogen is positioned well away from the target carbonyl. OSM-LO-88 also exhibited very low activity. In many of the lowest energy docks, the oxo-thienopyrimidine adopts a very different position to the aminothienopyrimidine, with the oxy group pointing in the opposite direction to the amine of OSM-S-106 (Supplementary Fig. 11G). Again, these results suggest that the reactive pose of the pro-inhibitor is important for antimalarial potency, which in these two cases is governed by correct positioning of the thienopyrimidine ring.

Of note is OSM-S-137, a compound with some antimalarial potency and possessing a large substituent on the thienopyrimidine ring. The substituent cannot be accommodated within the AMP pocket and in the lowest energy docked conformation, OSM-S-137 is positioned in the reverse orientation at the active site, relative to OSM-S-106 (Supplementary Fig. 11H). This is consistent with our finding that OSM-S-137 did not induce eIF2α phosphorylation (Fig. 2B); and suggests that the low-level anti-plasmodial potency of this compound may be off-target.

The modelling described here will be valuable in the design of future OSM-S-106 variants, and potentially in the design of pro-inhibitors of other aaRSs, where suitable distances and geometries are essential in addition to appropriate docking scores.

## Discussion

Nucleoside sulfamates are an exciting class of enzyme pro-inhibitors. They have been shown to induce certain ubiquitin activating (E1) enzymes to synthesise potent inhibitory adducts of the nucleoside sulfamate with enzyme-bound ubiquitin-like proteins. This reaction mechanism is powerful and has resulted in multiple new clinical candidates that target E1 enzymes (*e.*g., Pevonedostat, TAK-243 and TAK-981 ^30, 31, 32^). More recently, some aaRSs were found to be susceptible to reaction hijacking by nucleoside sulfamates, in this case, attacking the enzyme bound charged tRNA and forming an inhibitory adduct with the amino acid. The identification of the *Plasmodium*-specific pyrazolopyrimidine sulfamate pro-inhibitor, ML901, opened the possibility of developing bespoke pro-inhibitors that target different *Pf*aaRSs.

ML901 exhibits excellent potency and selectivity, and effects single-dose cure in a mouse model of *P. falciparum* malaria. However, the nucleoside scaffold exhibits low lipophilicity (AlogP of ML901 is 0.069) ^6^, which may limit its oral bioavailability. Here, we explored an aminothienopyrimidine-based sulfonamide, OSM-S-106, which was first identified in a screen of GSK compounds and has since been explored as part of an Open Source Malaria initiative. OSM-S-106 exhibits drug-like properties with a synthetically accessible scaffold and favourable lipophilicity characteristics (AlogP 1.65). OSM-S-106 exhibits good activity against cultures of *P. falciparum.* Importantly, OSM-S-106 also prevents development of liver stage parasites, suggesting that this class of compound could be used for prophylaxis as well as treatment. One important characteristic of new antimalarial compounds is that they should exhibit a low propensity for resistance. We found that no resistant parasites emerged from an inoculum of 2.4 × 10^8^ exposed parasites, which compares well with other compounds selected for development^33^. While OSM-S-106 is stable during incubation with human microsomes and rat hepatocytes, it shows rapid intrinsic clearance in mouse microsomes. This complicates studies of pharmacokinetic properties in mice. Here we focused on *in vitro* analyses.

We used a gradual ramp-up method to evolve resistant parasites with a view to obtaining insights into the target of OSM-S-106. Following two months of selection we retrieved parasites exhibiting four-fold resistance that harboured an R487S mutation or a Copy Number Variant (CNV) in cytoplasmic *Pf*AsnRS. We showed that transfectants harbouring the *Pf*AsnRS_R487S_ mutation have decreased sensitivity to OSM-S-106, while down-regulation of *Pf*AsnRS enhanced sensitivity, validating *Pf*AsnRS as a target.

Interestingly, some of the parasite clones also exhibited mutations in the *P. falciparum* nucleoside transporter 4 (*Pf*NT4). Moreover, down-regulation of *Pf*NT4 enhanced sensitivity to OSM-S-106. Indeed, enhanced sensitivity was observed even in the presence of anhydrotetracycline (aTC), potentially due to low level down-regulation. A similar base level sensitisation has been observed for a *Pf*Hsp70 inhibitor in an aptamer-regulated *Pf*Hsp70 line^34^. *Pf*NT4 is a putative purine transporter that has been shown to be dispensable for blood stage growth but required for sporozoite colonization of salivary glands^19, 35, 36^. It is possible that *Pf*NT4 transports OSM-S-106 away from its primary site of action and that mutations in *Pf*NT4 enhance the accumulation of OSM-S-106. Further work is needed to test this possibility.

We showed that treatment of cultures with OSM-S-106 inhibits protein translation and triggers eIF2α phosphorylation, which is diagnostic of the presence of uncharged tRNA^16, 17^, providing further evidence that OSM-S-106 exerts its activity by inhibiting tRNA charging. If OSM-S-106 indeed inhibits *Pf*AsnRS via a reaction hijacking mechanism, Asn-OSM-S-106 adducts would be generated in the active site. Using targeted mass spectrometry, we detected a strong signal for Asn-OSM-S-106. Of interest, minor MS peaks were also detected for the adducts of glycine and alanine, when cultures were treated with a high concentration of OSM-S-106 (10 μM). This suggests that *Pf*GlyRS and *Pf*AlaRS, both of which are also class II aaRSs, are susceptible to hijacking by OSM-S-106. Our findings that down-regulation of *Pf*GlyRS and *Pf*AlaRS did not enhance susceptibility to OSM-S-106, and that glycine and alanine adducts were not detected in the extracts with 1 M OSM-S-106 treatment suggest that *Pf*AsnRS is the main target. However, even partial inhibition of *Pf*GlyRS and *Pf*AlaRS may enhance the action of OSM-S-106 and underpin the difficulty of evolving resistance.

We generated recombinant *Pf*AsnRS, *Pf*AsnRS_R487S_ and *Hs*AsnRS. ATP consumption by the three enzymes is greatly enhanced by addition of tRNA, consistent with productive aminoacylation. We found that commercially available *E. coli* tRNA was effective as a substrate for all three enzyme preparations, which facilitated the comparison. These biochemical data suggest that the *Pf*AsnRS_R487S_ mutation does not affect enzyme activity, consistent with the lack of any obvious growth phenotype.

OSM-S-106 inhibited ATP consumption by *Pf*AsnRS, but only in the presence of tRNA. This is consistent with the reaction hijacking mechanism. The concentration of OSM-S-106 (~1 μM) needed to induce 50% inhibition of ATP consumption is much higher than the amount needed to kill parasite cultures (~60 nM). This may be due to the fact that, in our biochemical assay, the enzyme first needs to generate the charged tRNA product, which is then attacked by the pro-inhibitor to generate the Asn-OSM-S-106 adduct. Tight binding of the adduct prevents the enzyme from undergoing further catalytic cycles. By contrast, in cells, tRNAs are generally fully loaded^37^; and may be able to rebind onto the aaRS, which may promote adduct formation. In addition, sulfonamide- and sulfamate-containing compounds are known to bind to red blood cell carbonic anhydrase^18, 38^ which may facilitate accumulation of OSM-S-106 into parasitised red blood cells.

Recombinant *Pf*AsnRS_R487S_ is less susceptible to inhibition by OSM-S-106 than wildtype *Pf*AsnRS. It is interesting to consider how this mutation might decrease the sensitivity of the enzyme to hijacking by OSM-S-106. Residue R487 lies in a helix that partially caps the active site; and is close to the tRNA binding site. Of interest, analysis of a hybrid structural mode of tRNA-bound AsnRS from the filarial nematode *Brugia malayi*^24^ revealed an important role for the equivalent residue, R425; this residue is involved in a salt-bridge interaction that needs to be broken to allow access of the 3’ end of tRNA to the active site. Thus, the R487S mutation may affect the stability of the complex of *Pf*AsnRS with the Asn-tRNA product, which may in turn affect residence time of the bound Asn-tRNA and therefore susceptibility to reaction hijacking.

OSM-S-106 does not inhibit ATP consumption by *Hs*AsnRS, and targeted mass spectrometry revealed that *Hs*AsnRS produces very little Asn-OSM-S-106 adduct. A major structural difference between *Pf*AsnRS and *Hs*AsnRS is the presence of a large *Plasmodium*-specific insert, adjacent to the flipping loop. This flipping loop is known to lock the activated Asn-AMP intermediate in place but to ‘flip’ out of the way to allow the tRNA acceptor stem to insert adjacent to the active site^39^. The presence of the large insert in *Pf*AsnRS may increase the time the Asn-tRNA product remains bound to the enzyme. This may enable AMP to vacate the active site and OSM-S-106 to bind to the site and mount a nucleophilic attack on the susceptible carbonyl group in Asn-tRNA. By contrast, the Asn-tRNA product may be released more rapidly from *Hs*AsnRS, thus limiting the opportunity for reaction hijacking.

Initial attempts to crystallize CD*Hs*AsnRS in the presence of ATP and Asn, with a view to capturing the complex with the activated Asn-AMP intermediate were not successful. Therefore, we employed the more slowly hydrolysable ATP analogue, AMPPNP. Diffraction quality crystals were obtained under these conditions. A comparison of the apo and Asn-AMP bound structures reveals stabilisation of two flexible regions. A four amino acid stretch between the beta hairpin and Motif I is stabilised by the formation of a contact between E279 and the Asn part of the ligand. Motif II, which lies further towards the C-terminus, is intersected by a second flexible loop. In the presence of bound Asn-AMP, R322 and E324 in Motif II are stabilised by an interaction with the adenylate part of the ligand, further contributing to binding the activated Asn-AMP intermediate.

While *Hs*AsnRS is unable to generate the Asn-OSM-S-106 complex, it is able to bind the synthetic adduct, as evidenced by inhibition of the consumption of ATP in the presence of Asn-OSM-S-106. We generated a high-resolution structure of *Hs*AsnRS in complex with synthetic Asn-OSM-S-106. The OSM-S-106 is positioned in the adenylate binding pocket with the sulfonamide-carbonyl bond overlaying the position of the phosphate-carbonyl bond of Asn-AMP and the sulfamate-carbonyl bond of Asn-AMS. Interestingly, however, the benzine ring of OSM-S-106 lies in the same plane as the thienopyrimidine ring. By contrast, the ribose of the adenylate containing structures is twisted with respect to the nucleoside.

Our attempts to solve the crystal structure of *Pf*AsnRS at high resolution were not successful, possibly due to the presence of the large insert and an extended N-terminal domain. Previous work has shown that these domains are needed for correct folding of *Pf*AsnRS^26^. We therefore generated a molecular model of *Pf*AsnRS bound to Asn-tRNA, building on structural information for *Hs*AsnRS and *Ec*AspRS/tRNA. The model represents the product-bound form of the enzyme primed for binding of OSM-S-106 or other potential AMP mimics. OSM-S-106 docks into *Pf*AsnRS with the benzene ring twisted toward planarity with the thienopyrimidine ring, in a pose similar to that observed in the crystal structure of *Hs*AsnRS/Asn-OSM-S-106 complex. The position of the sulfonamide nitrogen overlaps with that of the AMP phosphate and the AMS sulfamate nitrogen. Similarly, the amino groups on the pyrimidine rings of OSM-S-106, AMP and AMS all align closely. These docking studies illustrate that potent pro-inhibitors must bind in a manner that precisely positions the reactive sulfonamide to attack the carbonyl carbon of Asn-tRNA.

Interrogation of the lower energy docked conformations of the lower potency OSM-S-106 derivatives reveals a failure to position correctly either the sulfonamide nitrogen or the pyrimidine nitrogen. The work provides insights into the very subtle positioning requirements for reaction hijacking to occur; and provides a basis for the design of new compounds with improved activity.

In summary, this work identifies *Pf*AsnRS as a *P. falciparum* aaRS that can be specifically targeted by reaction hijacking; and identifies OSM-S-106 as an exemplar of a new chemical class of species-specific reaction hijacking inhibitor. The ability to selectively hijack particular aaRSs provides a new way to inhibit a class of enzymes that are considered good drug targets in *Plasmodium* and other infectious organisms^23, 40, 41^. Our biochemical, structural, and modelling studies reveal the molecular correlates of potent antimalarial activity. This work will help in the development of new, much needed, antimalarial therapies.

## Supplementary Material

Supplement 1

## Figures and Tables

**Table 1.** Cytotoxicity of OSM-S-106 and derivatives against *P. falciparum* (3D7) and the HepG2 mammalian cell line. n = Number of biological repeats. Where available, data are expressed as mean ± STD DEV.

Compound	OSM-S-106	OSM-E-32	OSM-S-137	OSM-S-488	OSM-LO-80	OSM-LO-81	OSM-LO-87	OSM-LO-88
*Pf*3D7 IC_50(72h)_ (μm)	0.058 ± 0.017 (n = 8)	8.3/8.3 (n = 2)	4.4 ± 2.6 (n = 4)	12.7/13.8 (n = 2)	2.1 ± 0.4 (n = 4)	0.93 ± 0.22 (n = 6)	>25 (n =6)	18.7 ± 1.8 (n = 6)
HepG2 IC_50(72h)_ (μM)	49.6/47.3 (n = 2)	N/A	N/A	N/A	17.2/18.2 (n = 2)	43.4/43.9 (n = 2)	N/A	N/A
*P. berghei* liver stage IC_50(72h)_ (μm)	0.25/0.42 (n=2)	N/A	N/A	N/A	N/A	N/A	N/A	N/A

**Table 2.** Mutations identified in Dd2 parasites selected with OSM-S-106 and quality metrics for each sequenced parasite line. Six clones from two independent OSM-S-106-pressured cultures were sequenced and analysed to identify potential resistance-conferring variants. Variants with ≥90% alleles mapping to the alternate allele are shown.

Parasite Name	Gene	Gene	Gene	CNV	IC_50_ (μm)	IC_50_ (fold increase)
Dd2-B2–2	NRS parent	NT4 Parent	GDH Parent	none	0.079 ± 0.012 (n = 4)	1.0
Dd2-OSM-2A6	NRS R487S	NT4 H320L	GDH Parent	none	0.70	8.8
Dd2-OSM-2B2	NRS R487S	NT4 H320L	GDH Parent	none	0.70	8.8
Dd2-OSM-2A9	NRS R487S	NT4 Parent	GDH Parent	NRS	0.32	4.1
Dd2-OSM-2D6	NRS R487S	NT4 H320L	GDH Parent	none	0.78	9.9

Dd2-B2–3	NRS parent	NT4 Parent	GDH Parent	none	0.08	1.0
Dd2-OSM-3E5	NRS parent	NT4 S22C	GDH D200Y	NRS	0.16	2
Dd2-OSM-3H7	NRS parent	NT4 S22C	GDH D200Y	NRS	0.22	2.8

## Data Availability

Additional data are available in Supplementary Information. Source data are provided with this paper. The datasets generated and analysed during the current study are available from the corresponding author on reasonable request. Additional data are available in the SI Appendix. The following structures have been deposited in the PDB: *Hs*AsnRS/Asn-AMP - PDB 8H53; *Hs*AsnRS (apo) – PDB 8TC7; *Hs*AsnRS/Asn-AMS – PDB 8TC8; *Hs*AsnRS/Asn-OSM-S-106 – PDB 8TC9.
