# Supplementary material for: Reaction hijacking inhibition of Plasmodium falciparum asparagine tRNA synthetase
Source: Res Sq. 2023 Jul 27:rs.3.rs-3198291. Preprint. [Version 1] doi: 10.21203/rs.3.rs-3198291/v1 (PMC10402266; doi:10.21203/rs.3.rs-3198291/v1)
Supplement: Supplement 1 [file NIHPPrs3198291v1-supplement-1.pdf]

Supplementary Materials for
Reaction hijacking inhibition of *Plasmodium falciparum* asparagine tRNA synthetase

Stanley C. Xie, Yinuo Wang, Craig J. Morton, Riley Metcalfe *et al*.
Corresponding authors: Elizabeth Winzeler (ewinzeler@health.ucsd.edu), Michael Griffin (mgriffin@unimelb.edu.au), Matthew Todd (matthew.todd@ucl.ac.uk), Leann Tilley (ltilley@unimelb.edu.au)

The PDF file includes:

Figures S1 to S11
Tables S1 to S9
Biological Materials and Methods
Chemistry Materials and Methods
Supplementary References

Other Supplementary Material for this manuscript includes the following:
Dataset 1: Synthetic chemistry characterisation dataset
Dataset 2: CNV analysis for DSM265 selection
Dataset 3: SNVs and INDEL analysis for OSM-S-106 selection
Dataset 4: CNV analysis for OSM-S-106 selection

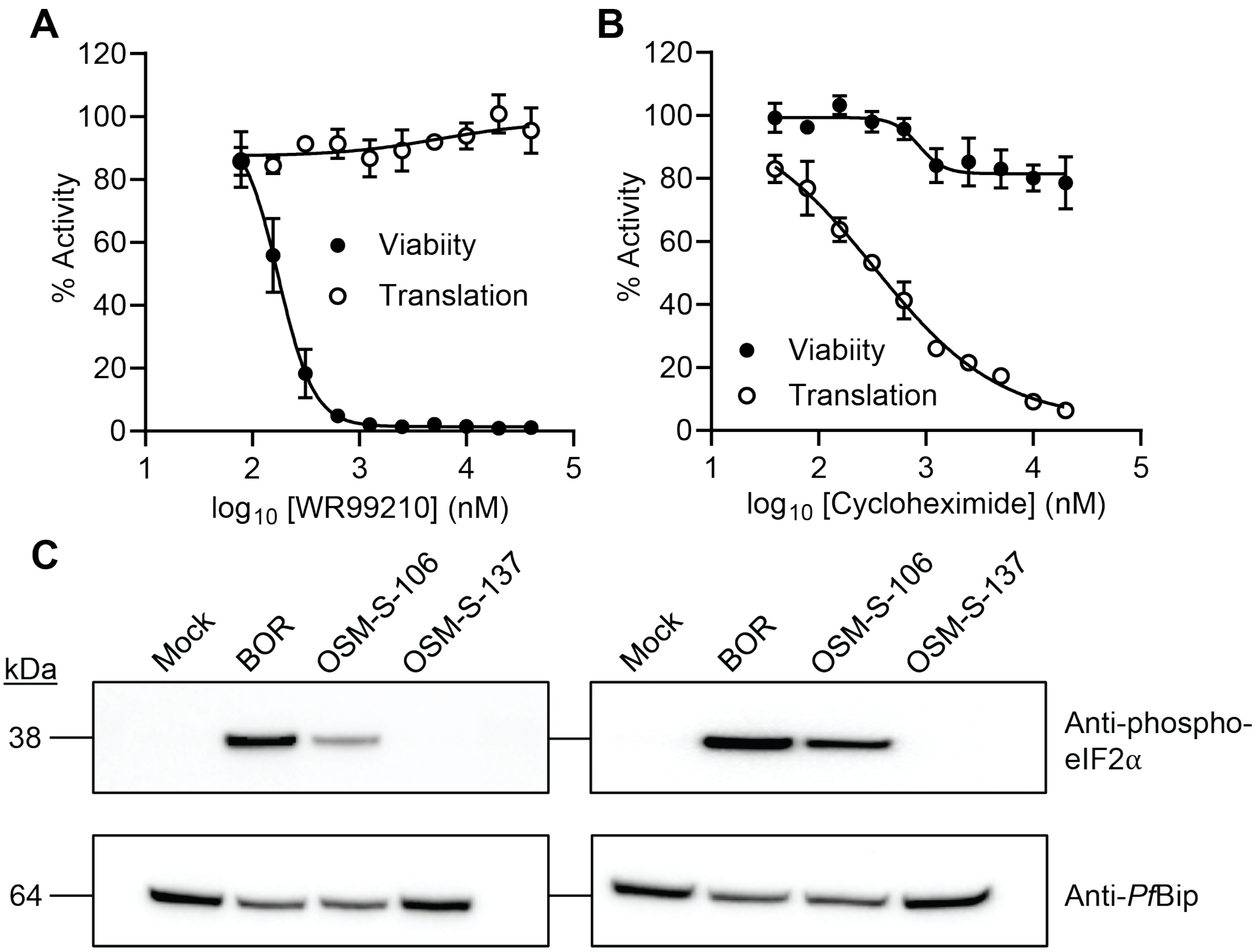

Fig. S1. Effects of compounds on protein translation inhibition and eIF2α phosphorylation.
(**A, B**). *P. falciparum* cultures (Cam3.II-rev; trophozoite stage; 30-35 h p.i.) were exposed to *Pf*DHFR inhibitor, WR99210 (**A**) or protein translation inhibitor, cycloheximide (**B**) for 6 h. Protein translation was assessed in the last two hours of the incubation, via the incorporation of OPP. Aliquots of inhibitor-exposed cultures were washed and returned to cultures, and viability was estimated at the trophozoite stage of the next cycle. WR99210: IC_{50} (Translation) > 1 μM, IC_{50} (Viability) = 0.9 nM. Cycloheximide: IC_{50} (Translation) = 331 nM, IC_{50} (Viability) > 1 μM. Error bars correspond to SEM of three independent experiments. (**C**) Trophozoite stage Cam3.II_rev parasites (30-35 h p.i.) were incubated with 0.05% DMSO (Mock), 50 nM borrelidin (BOR) or 2.5 μM OSM-S-106 or 2.5 μM OSM-S-137 for 3 h. Western blots of lysates were probed for phosphorylated-eIF2α with *Pf*BiP as a loading control. The data represent additional blots related to data presented in Fig. 2B.

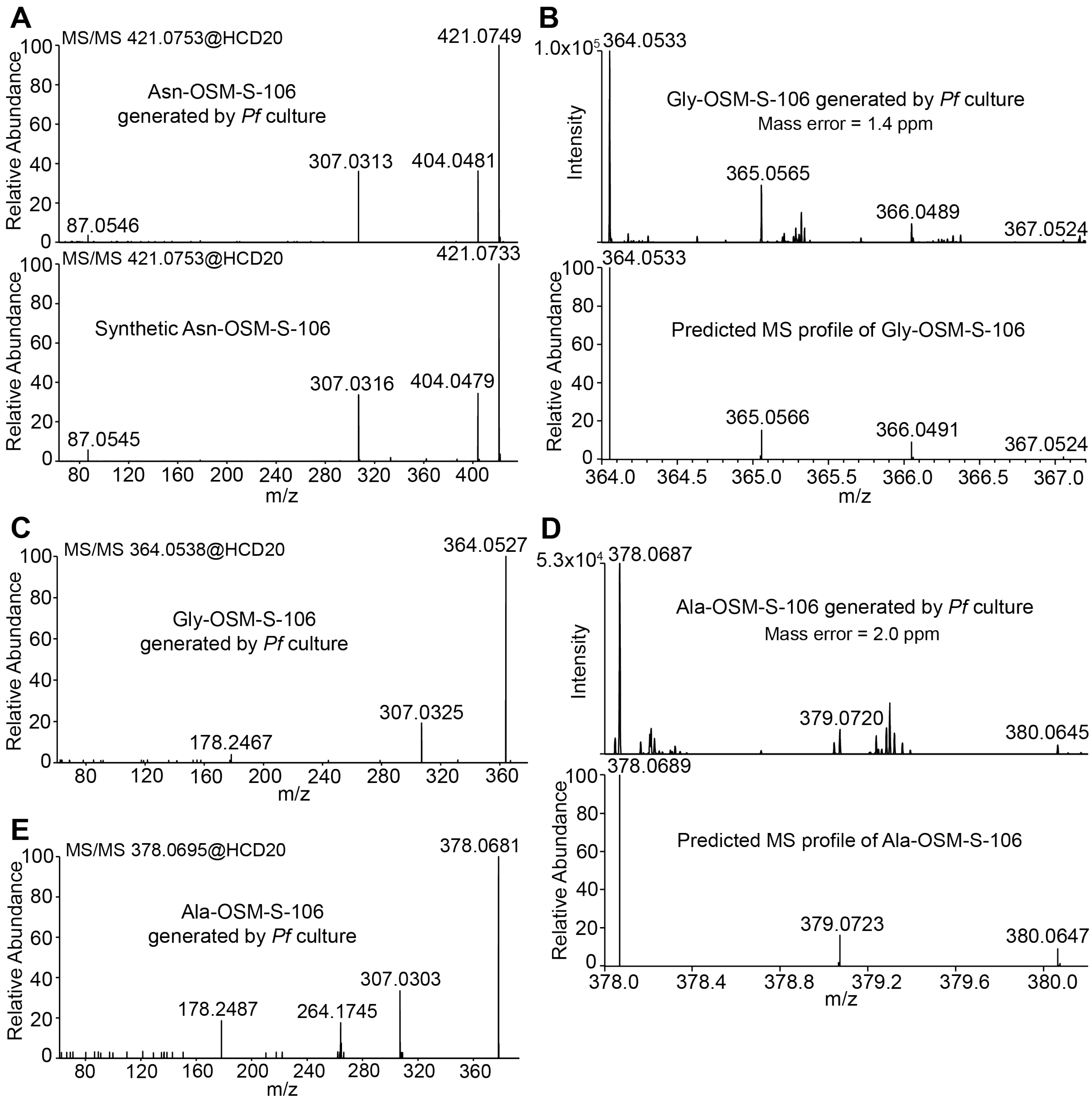

Fig. S2. **Production of Asn-OSM-S-106, Gly-OSM-S-106 and Ala-OSM-S-106 adducts by *P. falciparum* cultures.**

P. falciparum cultures were treated with 10 µM OSM-S-106 for 3 h. Extracts were subjected to LCMS. (**A**) MS/MS analysis of detected Asn-OSM-S-106 adduct compared with the synthetic conjugate. MS (**B, D**) and MS/MS (**C, E**) analysis of detected Gly-OSM-S-106 (**B, C**) and Ala-OSM-S-106 (**D, E**) from cell extracts. (**B, D**) Upper panels show the protonated adducts made by the parasites and lower panels show the predicted mass spectra.

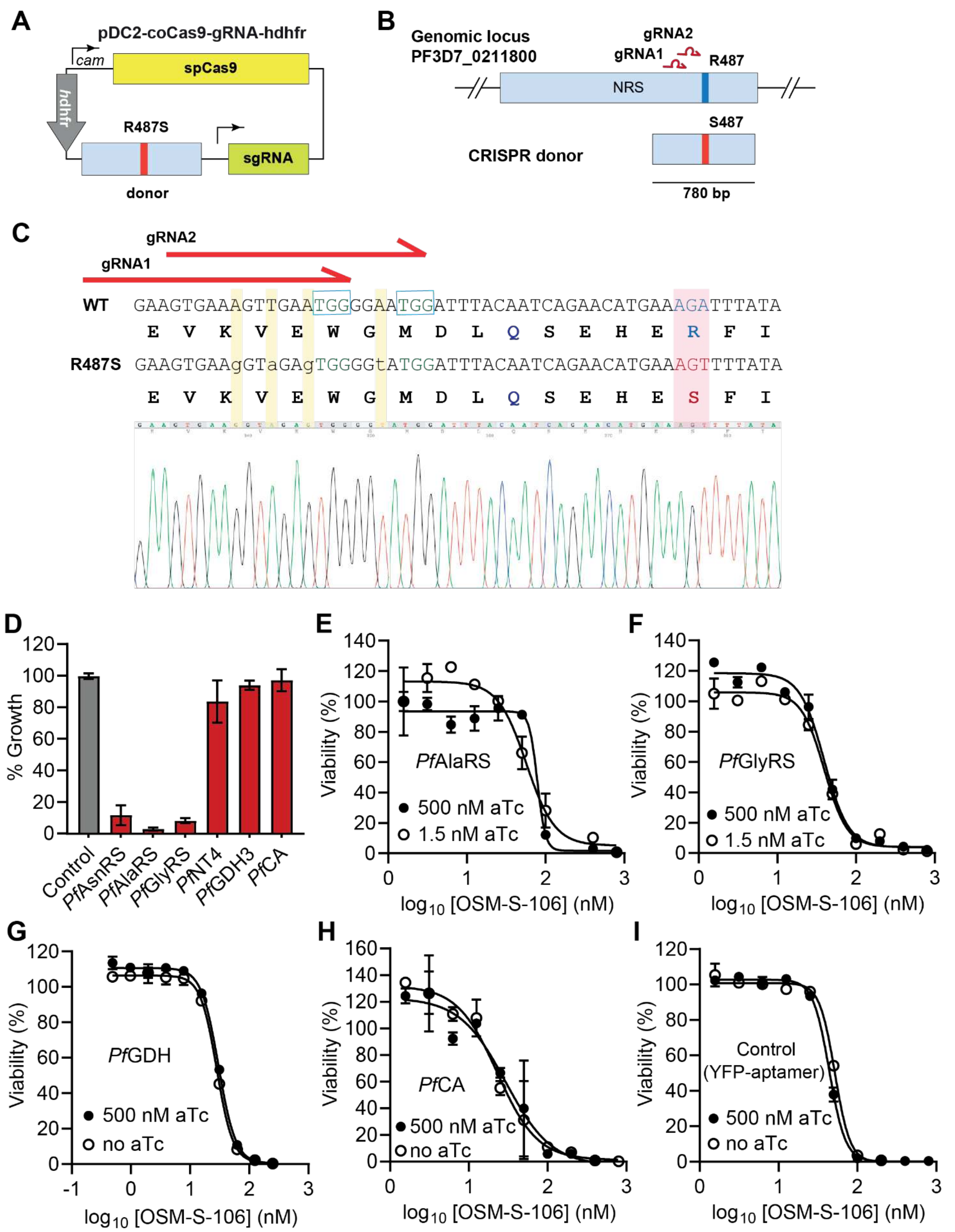

Fig. S3. Generation of CRISPR-edited *P. falciparum* transfectants harbouring the AsnRS R487S mutation, and aptamer-regulatable lines for several genes identified as potential targets.

(**A**) Plasmid map illustrating the all-in-one Cas9-gRNA-donor plasmid used for editing the R487S mutation in *PfAsnRS* (Dd2 background). (**B**) Genomic locus of the *PfAsnRS* gene (PF3D7_0211800). The R487S mutation was edited using a 780 bp donor template. (**C**) Sequence of the edited region, indicating the binding site of the two gRNAs and their corresponding PAM motifs (blue boxes), the R487S codon (highlighted in red) as well as the silent shield mutations (yellow) that were introduced to prevent gRNA binding. (**D**) Growth of aptamer-regulatable *Pf*AlaRS, *Pf*GlyRS, *Pf*GDH3, *Pf*CA lines and a control YFP line was assessed over 72 h, relative to aTc-treated controls. Error bars correspond to SEM or range of 2-4 independent experiments. (**E-I**) Sensitivity to OSM-S-106 exposure (72-h) of aptamer-regulatable *Pf*AlaRS, *Pf*GlyRS, *Pf*GDH3, *Pf*CA lines and a control YFP line, upon addition of aTc, with data normalized to a no drug control. See Supplementary Table 7 for data values.

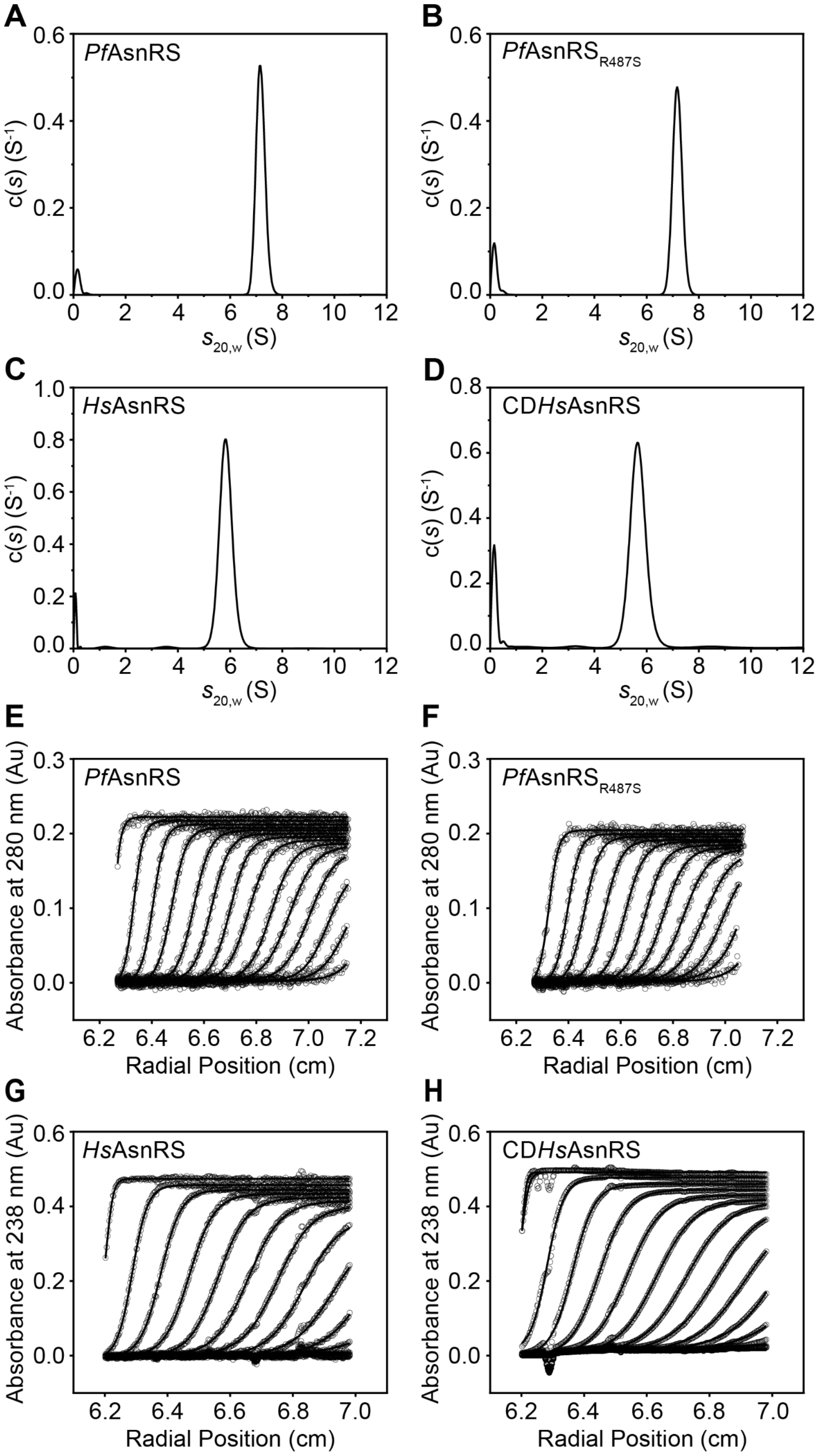

Fig. S4. Physical characterization of recombinant *Pf*AsnRS, *Pf*AsnRS$_{R478S}$, full-length *Hs*NRS and CD*Hs*AsnRS. Continuous sedimentation coefficient (c(s)) distributions derived from sedimentation velocity analytical ultracentrifugation for *Pf*AsnRS (**A**), *Pf*AsnRS$_{R478S}$ (**B**), *Hs*AsnRS (**C**), and CD*Hs*AsnRS (**D**). The measured sedimentation coefficients (s20,w) at 2.8 μM are 7.1 S for *Pf*AsnRS, 7.1 S for *Pf*AsnRS$_{R478S}$, 5.8 S for *Hs*AsnRS and 5.6 for CD*Hs*AsnRS, corresponding to masses of 141.1 kDa for *Pf*AsnRS, 141.0 kDa for *Pf*AsnRS$_{R478S}$, 126.0 kDa for *Hs*AsnRS and 103.7 kDa for CD*Hs*AsnRS, all consistent with a dimeric state in solution. Raw analytical ultracentrifugation-sedimentation velocity data for (**E**) *Pf*AsnRS, (**F**) *Pf*AsnRS$_{R478S}$, (**G**) *Hs*AsnRS, and (**H**) CD*Hs*AsnRS, overlaid with fits to a continuous sedimentation coefficient (c(s)) model, at 2.8 μM. For clarity, every second (**E,F**) or sixth (**G,H**) scan is shown.

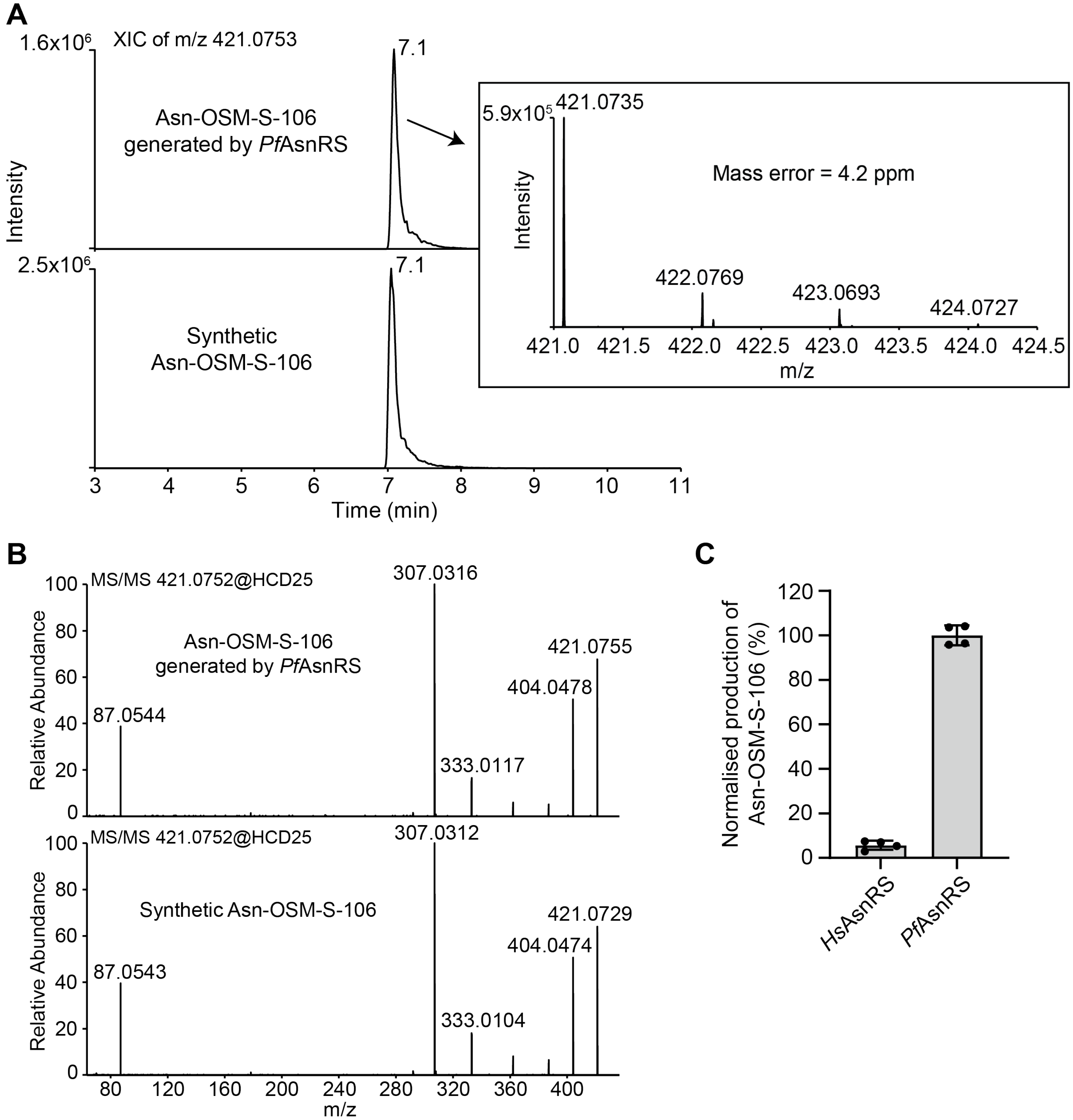

Fig. S5. **Recombinant *Pf*AsnRS is markedly more efficient in generating Asn-OSM-S-106 than *Hs*AsnRS.** *Pf*AsnRS or *Hs*AsnRS (1 µM) was incubated with OSM-S-106 (10 µM), ATP (10 µM), asparagine (20 µM) and *E. coli* tRNA (2.5 mg/mL) for 1 h at 37°C. Following protein denaturation and precipitation, the supernatant was subjected to LCMS analysis. (**A**) The extracted ion chromatograms showing Asn-OSM-S-106 generated by *Pf*AsnRS (upper panel), and the synthetic conjugate (3 µM, lower panel), at m/z 421.0753. The inset shows MS analysis of the enzyme-generated Asn-OSM-S-106. (**B**) MS/MS analysis of *Pf*AsnRS-generated Asn-OSM-S-106 (upper panels) compared with the synthetic conjugate (lower panels). (**C**) The amount of Asn-OSM-S-106 produced by *Pf*AsnRS and *Hs*AsnRS is quantified based on the intensity of the peaks at m/z 421.0752 from the extracted ion chromatograms. Results are normalised using Asn-OSM-S-106 made by *Pf*AsnRS as 100%. Data represent mean ± SD from four independent experiments.

```
Pv  --------------------------------MGATFSRLALSTTLLGLLARGVHRGEGRAEASALALQLCGQRGRRRRAGRRAEAYGGRTTRRTATSGG  68
Pf  ----------------------------------------------------------------------------------------------------
Hs  ----------------------------------------------------------------------------------------------------
Sc  ----------------------------------------------------------------------------------------------------
Bm  ----------------------------------------------------------------------------------------------------
Eh  ----------------------------------------------------------------------------------------------------


Pv  TTRRTTPGGTPLMRGAFIFKGNAHHSWCISRGTPNALGWSTPPVRRRFFAKVRGGSGSQGGGHDGAHPGGAPNESASVAPHPGGAPHESASVAPHPGGAS 168
Pf  -----------------------------------------------------------MCEKDDITVNEEILQKAQEFQVENEKDIKMKKLKPITEGLL  41
Hs  ----------------MVLAELYVSDREGSDATGDGTKEKPFKTGLKALMTVGKEPFPTIYVDSQKENERWNVISKSQLKNIKKMWHREQMKSESREKKE  84
Sc  -------------MSSLYIKEATGVDELTTAGSQDHPFKTPAYALFASQQKSDATEPKLFVFKTEDNEYQEISASALKKARKGCDGLKKKAVKQKEQELK  87
Bm  ------------------MTVYICPETGDDGNDGSELKPLRTLYQAMIITKSSKGDFLIRTKKDGKQIWEAASKTALKKSWKHYEQEMLKNEKVAAKMLE  82
Eh  ----------------------------------------------------------------------------------------------------


Pv  NEGTSSELLPGEASNERTGRTKIKDILR--------------AQGTHSEGETCTICGWVKSVRTVGKNRFSFIDVNDGSHVKNLQVVVDAGIPNYGEVSR 254
Pf  KPE--VDLLQ-ISERGSRGRVKICNVLNVPRSEKEYNNNNSDKVENKYIGKIITVCGWSKAIRKQGGGRFCFVNLNDGSCHLNLQIVVNQCIENYEKLLK 138
Hs  -AEDSLR---REKNLEEAKKITIKNDPSLP-----EPKCVKIGALEGYRGQRVKVFGWVHRLRRQGKN-LMFLVLRDGTGYLQCVLAD--ELCQCYNGVL 172
Sc  KQQKEAE---NAAKQLSALNITIKEDESLP-----AAIKTRIYDSYSKVGQRVKVSGWIHRLRSN-KK-VIFVVLRDGSGFIQCVLSG--DLALAQQTLD 175
Bm  KDATEVG---VKAALEEAKKVQIELDTSLS-----YITGVKIRDLVKHRNERVCIKGWIHRMRRQGKS-LMFFILRDGTGFLQVLLMD--KLCQTYDALT 171
Eh  --------------------MTEATTTPVE-----TPIVCNIRDAAGLEGKLVTFKGWAYHIRKARKT-LIFVELRDGSGYCQCVIFG-KELCEPEKVKL  73
                                                     .:   . **   :*      . *. :.**:   :  :     :    .

Pv  VLTDDAVECSGELKCSLGKKQRVELCVRD--AARAHYVRLLGRGEGARSGGGSEGGSADGEGEKRDDGEGDRRDDGEGDRRDDGEGDRRGKHYAIAKKYH 352
Pf  CGAGCCFRFTGELIISPVQND----------NN--------------K------------KGLLKENVELALNNN-DIHNFEIYGENLDPQKYPLSKKNH 201
Hs  LSTESSVAVYGMLNLTPKGKQAPG-----GHELSCDFWELIGLAP---------AGGA--DN--------------------------------LINEES 224
Sc  LTLESTVTLYGTIVKLPEGKTAPG-----GVELNVDYYEVVGLAP---------GGEDSFTN--------------------------------KIAEGS 229
Bm  VNTECTVEIYGAIKEVPEGKEAPN-----GHELIADFWKIIGNAP---------PGGI--DN--------------------------------VLNEEA 223
Eh  LTRECSLEITGRLNAYAGKNHPPEIADILNLEMQVTEWKVIGESP---------I---DLEN--------------------------------IINKDS 129
          .   * :      :                                         .                                   :
                                                 Mobile residues
                          Motif I                Flipping Loop                 LCR
Pv  SKEHLRSFPHLRARTKLYSCVFRLKSDVVAETFKFWRERNCTYINTPVLTSNDCEGAGELFHATTLMSRRGSGGGGEA------------------NGSD 434
Pf  GKEFLREVAHLRPRSYFISSVIRIRNSLSIATHLFFQSRGFLYIHTPLITTSDCEGGGEMFTVTTLLNENGDIRSIPRINLKNKKKEKREDILNEKNGKK 301
Hs  DVDVQLNNRHMMIRGENMSKILKARSMVTRCFRDHFFDRGYYEVTPPTLVQTQVEGGATLF--------------------------------------- 285
Sc  DPSLLLDQRHLALRGDALSAVMKVRAALLKSVRRVYDEEHLTEVTPPCMVQTQVEGGSTLF--------------------------------------- 290
Bm  SVDKMLDNRHLVIRGENAAALLRLRAAATRAMREHFYNAGYLEVAPPTLVQTQVEGGSTLF--------------------------------------- 284
Eh  SIPQKMQNRHIVIRSEHTQQVLQLRSEIQWYFRKYYHDNHFTEIQPPTIVKTQCEGGSTLF--------------------------------------- 190
    .     .  *:  *      ::: :          : .     :  * :. .: **.. :*
                   LCR                                                     Mobile residues   Motif II
Pv  ---------------GSGGASGIDQRDERPGEPVKGDPPFCRDFFKKPCYLNVSSQLALECLCCSMGDVFTLNQSFRAEQSNTVRHLSEFLMMEVELAFS 519
Pf  DHMNDSLNNNTCNNNNNNGNSSSSNIVSSPQYEDNYLIDYKKDFFSKQAFLTVSGQLSLENLCSSMGDVYTFGPTFRAENSHTSRHLAEFWMIEPEIAFA 401
Hs  ----------------------------------------KLDYFGEEAFLTQSSQLYLETCLPALGDVFCIAQSYRAEQSRTRRHLAEYTHVEAECPFL 345
Sc  ----------------------------------------KMNYYGEEAYLTQSSQLYLETCLASLGDVYTIQESFRAEKSHTRRHLSEYTHIEAELAFL 350
Bm  ----------------------------------------NLDYFGEQSFLTQSSQLYLETCIPTLGDVFCIAQSYRAEKSRTRRHLAEYAHVEAECPFI 344
Eh  ----------------------------------------KLQYFNEPAYLTQSSQLYLESVIASLGKSFCMLSSYRAEQSRTVRHLAEYLHLEAELPFI 250
                                              ::: : .:*. *.** **    ::*. : :  ::***:*.* ***:*:  :* *  *

Pv  NLTSIISLAEEYIKTMVKFALHQS-EDVDYIHEHHDRTLKEKLQA---VLQKPFAVVTYDEAMQIVKRHVGRVGVAVDPHVAARTDVSQRGGAPPPRADL 615
Pf  DLYDNMELAEAYIKYCIDYVLNNNFHDIYYFEENVETNLIKRLKN---ILNEDFAKITYTNAIEILQNYSDSFEVKVE-----------------WGMDL 481
Hs  TFDDLLNRLEDLVCDVVDRILKSP-AGS----------IVHELNPNFQPPKRPFKRMNYSDAIVWLKEHDVKKE---------------DGTFYEFGEDI 419
Sc  TFDDLLQHIETLIVKSVQYVLEDPIAGP----------LVKQLNPNFKAPKAPFMRLQYKDAITWLNEHDIKNE---------------EGEDFKFGDDI 425
Bm  TLDDLMEKIEELVCDTVDRLLADEEAKK----------LLEHINPKFQPPERPFLRMEYKDAIKWLQEHNVENE---------------FGNTFTYGEDI 419
Eh  SFEDLLNHLEDLVCTVIDNVMAV--HGD----------KIRKMNPHLKLPTRPFKRMTYADAIKYCNDHGILNK---------------D-KPFEYGEDI 322
     : . :.  *  :   :.  :                   ..::         *  : *:*:   : :                              *:
    Mutant PfAsnRS R487S                                                                     Motif III
Pv  TFEEQRFLTEVHFRSPVVVVNYPQEIKPFYMALNAD-GKTVACMDVLLPHVGEVVGGSEREIRIHTLERRMKEKRLDLRLYEPYLQLRRQGNVPHAGFGL 714
Pf  QSEHERFIAEKIFKKPVIVYNYPKDLKAFYMKLNED-NKTVAAMDVLVPKIGEVIGGSQREDNLERLDKMIKEKKLNIDSYWWYRQLRQYGSHPHAGFGL 580
Hs  PEAPERLMTDTI-NEPILLCRFPVEIKSFYMQRCPEDSRLTESVDVLMPNVGEIVGGSMRIFDSEEILAGYKREGIDPTPYYWYTDQRKYGTCPHGGYGL 518
Sc  AEAAERKMTDTI-GVPIFLTRFPVEIKSFYMKRCSDDPRVTESVDVLMPNVGEITGGSMRIDDMDELMAGFKREGIDTDAYYWFIDQRKYGTCPHGGYGI 524
Bm  AEAAERFMTDTI-NKPILLNRFPSEIKAFYMQRDAQDNTLTESVDLLMPGVGEIVGGSMRIWKFDELSKAFKNVEIDPKPYYWYLDQRLYGTCPHGGYGL 518
Eh  SEKPERQMTDEI-GCPIFMIHFPSKMKAFYMSKVPGHPDLTESVDLLMPGVGEIVGGSMRIWNYDELMGAYKANGLNPDPYYWYTQQRKYGSCPHGGYGL 421
        :*  :::    *:.:.:* .:* ***         . .:*:*:* :**: *** *    . :    *   ::   *  : : *  *. **.*:*:
    Motif III
Pv  GVDRLIMFLTSMSNIRDVVPFPRAPGSLFM 744
Pf  GFERLIMLVTGVDNIKDTIPFPRYPGHAEF 610
Hs  GLERFLTWILNRYHIRDVCLYPRFVQRCTP 548
Sc  GTERILAWLCDRFTVRDCSLYPRFSGRCKP 554
Bm  GLERFICWLTNTNHIRDVCLYPRFVGRCVP 548
Eh  GVERLVMWLLGEDHIRKVCLYPRYLERCEP 451
    * :*::  : .   ::.   :**
```

Figure S6. Sequence alignment of AsnRS sequences from a range of species.

Type II aaRSs exhibit three motifs (I-III, grey shading) involved in ATP binding and dimerization. Alignment of AsnRS sequences from *Homo sapiens* (Hs), *Saccharomyces cerevisiae* (Sc), *Brugia malayi* (Bm), *Entamoeba histolytica* (Eh), *Plasmodium vivax* (Pv), and *Plasmodium falciparum* (Pf) reveals a high level of conservation

of these motifs. One *Plasmodium*-specific feature of interest is the presence of a large insert (highlighted in black, white font). R487 (Pf) and the equivalent residue in other species are indicated (bold, underline type). The flipping loop as defined by (Schmitt et al., 1998) is highlighted in yellow. The mobile residues that are stabilised upon ligand binding in the active site are boxed. The flipping loop that has previously been shown to undergo dynamic motions that facilitate tRNA binding (Schmitt et al., 1998) is highlighted in khaki green.

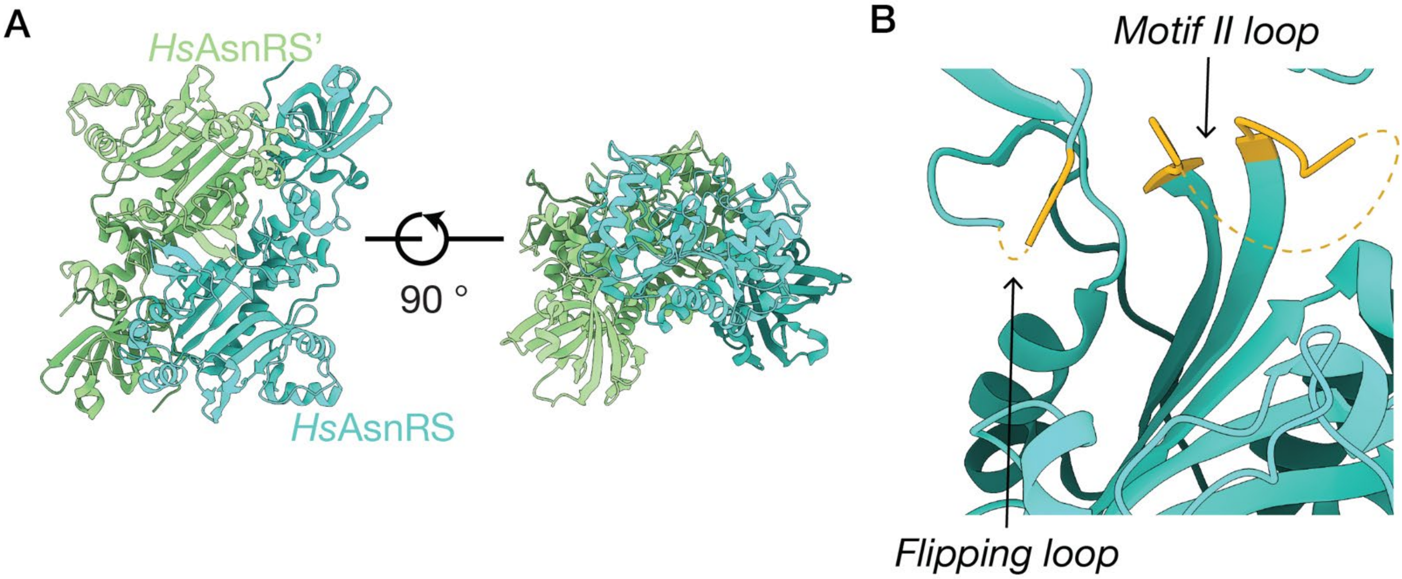

Figure S7. Crystal structure of apo *Hs*AsnRS.
(**A**) Ribbon diagram of the structure of dimeric apo *Hs*AsnRS showing monomers in light green and teal. (**B**) Ribbon diagram of the active site of apo *Hs*AsnRS showing the positions of the flipping loop and motif II loop (yellow). Density for some residues in these loops was insufficient to allow modeling, and missing residues are represented as dashed yellow lines.

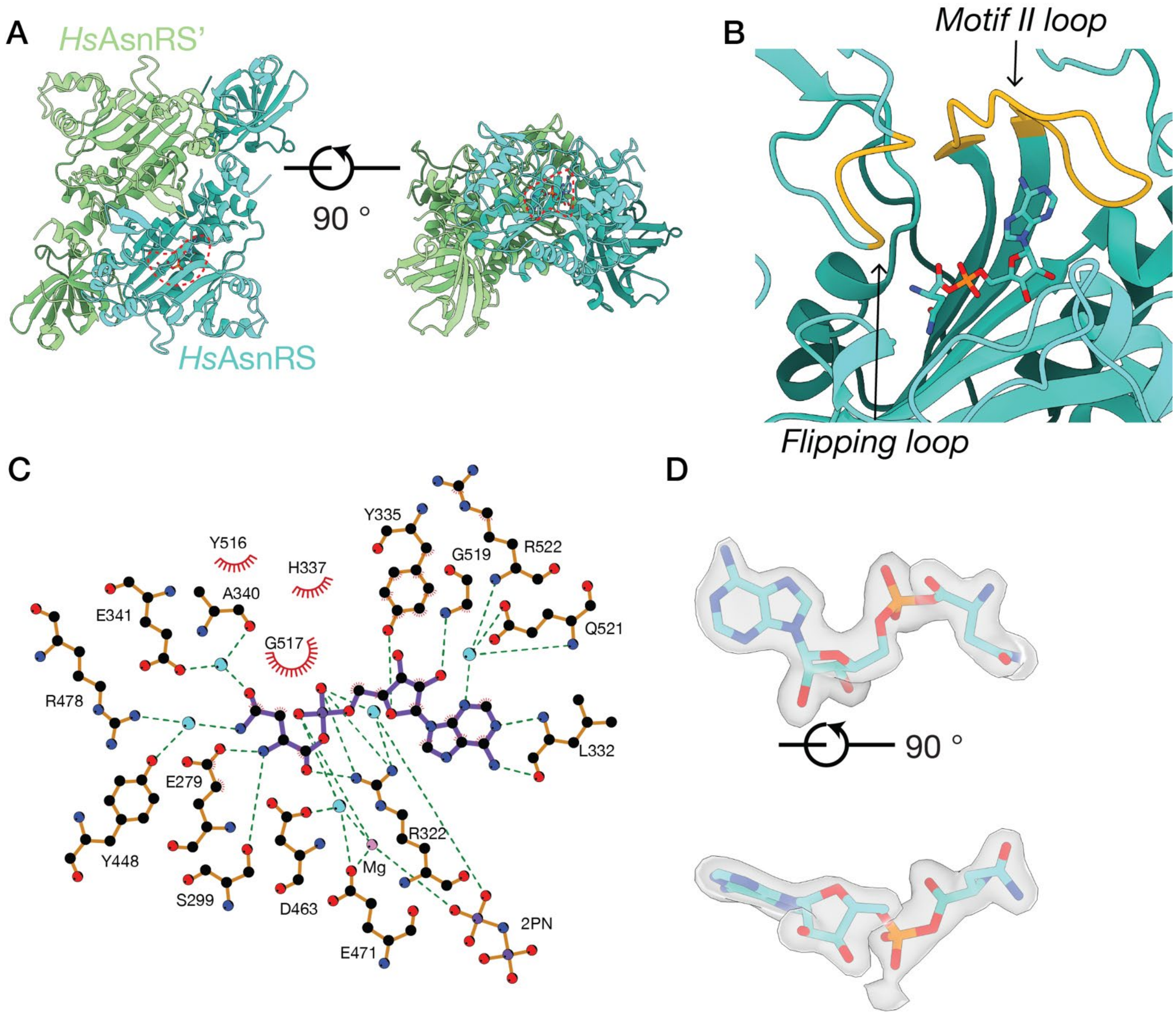

Figure S8. Crystal structure of *Hs*AsnRS in complex with Asn-AMP.

(**A**) Ribbon diagram of the structure of the dimeric Asn-AMP/*Hs*AsnRS complex showing monomers in light green and teal. Asn-AMP is shown in stick representation (circled with dashed red line). (**B**) Ribbon diagram of the active site of the Asn-AMP/*Hs*AsnRS complex showing the positions of the flipping loop and motif II loop (yellow). Asn-AMP is shown in stick representation. 2PN = imidodiphosphoric acid. (**C**) LigPlot map of interacting residues for the Asn-AMP/*Hs*AsnRS complex. (**D**) 2F_o-F_c maps contoured at 1.5 σ (grey surface) showing electron density supporting the position of Asn-AMP.

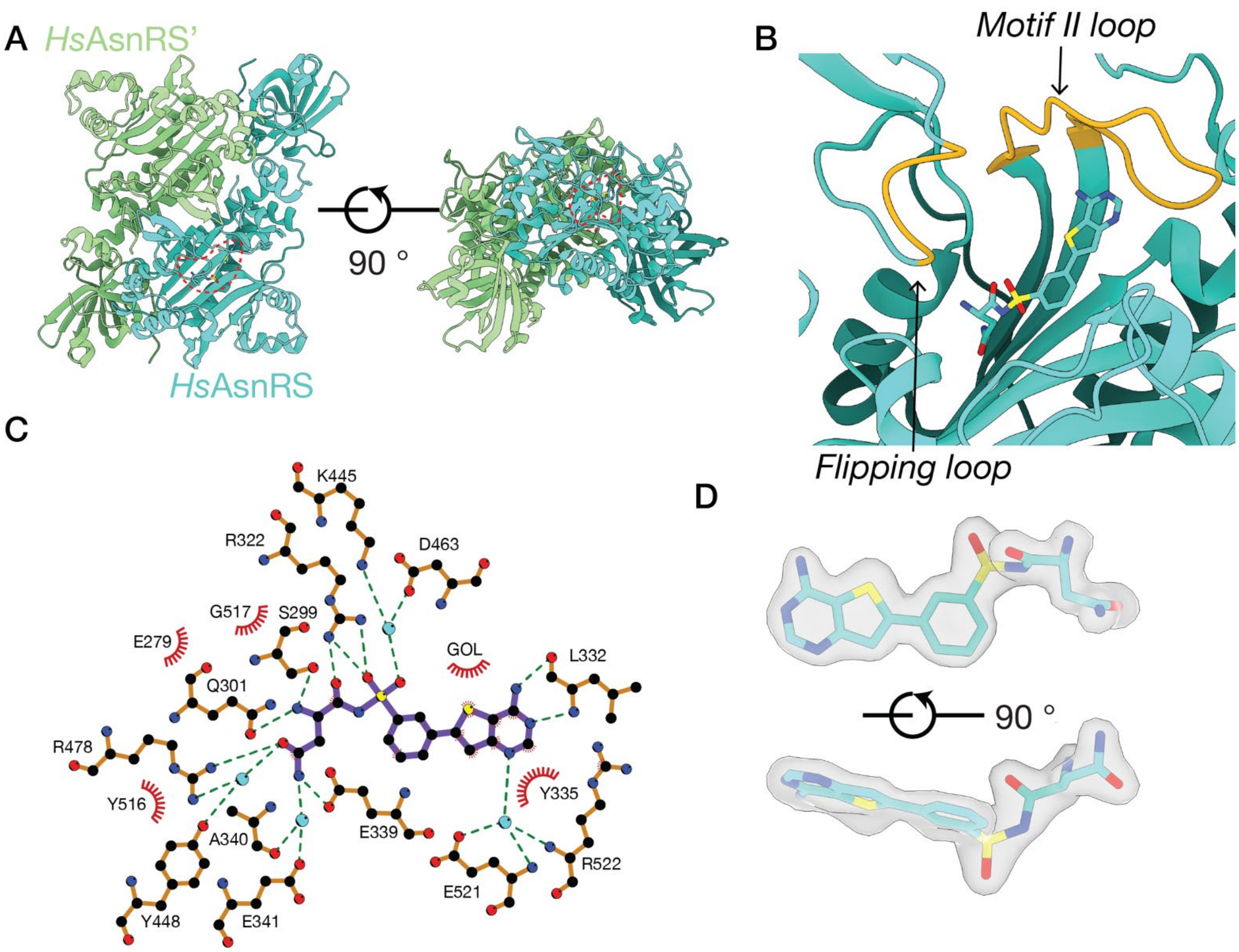

Figure S9. Crystal structure of *Hs*AsnRS in complex with Asn-OSM-S-106.
(**A**) Ribbon diagram of the structure of the dimeric Asn-OSM-S-106/*Hs*AsnRS complex showing monomers in light green and teal. Asn-OSM-S-106 is shown in stick representation (circled with dashed red line). (**B**) Ribbon diagram of the active site of the Asn-OSM-S-106 /*Hs*AsnRS complex showing the positions of the flipping loop and motif II loop (yellow). Asn-OSM-S-106 is shown in stick representation. (**C**) LigPlot map of interacting residues for the Asn-OSM-S-106/*Hs*AsnRS complex. GOL = glycerol (**D**) 2F_o-F_c maps contoured at 1.5 σ (grey surface) showing electron density supporting the position of Asn-OSM-S-106.

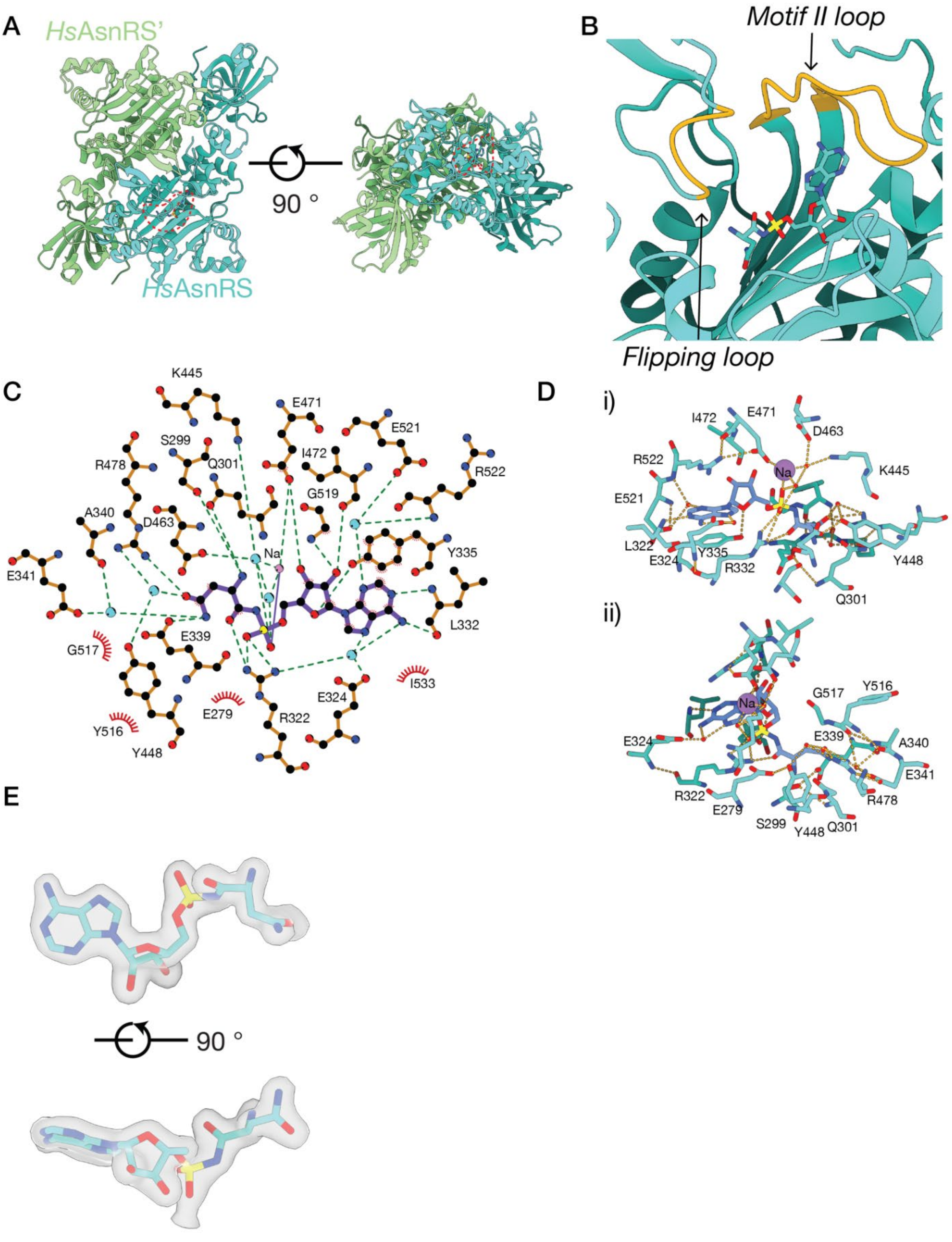

Figure S10. Crystal structure of *Hs*AsnRS in complex with Asn-AMS.

(**A**) Ribbon diagram of the structure of the dimeric Asn-AMS/*Hs*AsnRS complex showing monomers in light green and teal. Asn-AMS is shown in stick representation (circled with dashed red line). (**B**) Ribbon diagram of the active site of the Asn-AMS/*Hs*AsnRS complex showing the positions of the flipping loop and motif II loop (yellow). Asn-AMS is shown in stick representation. (**C**) LigPlot map of interacting residues for the Asn-AMS/*Hs*AsnRS complex. (**D**) Key inhibitor contact residues in the Asn-AMS/*Hs*AsnRS complex. Hydrogen bonds are indicated by yellow dashed lines. (**E**) $2F_o$-F_c maps contoured at 1 σ (grey surface) showing electron density supporting the position of Asn-AMS in chain A.

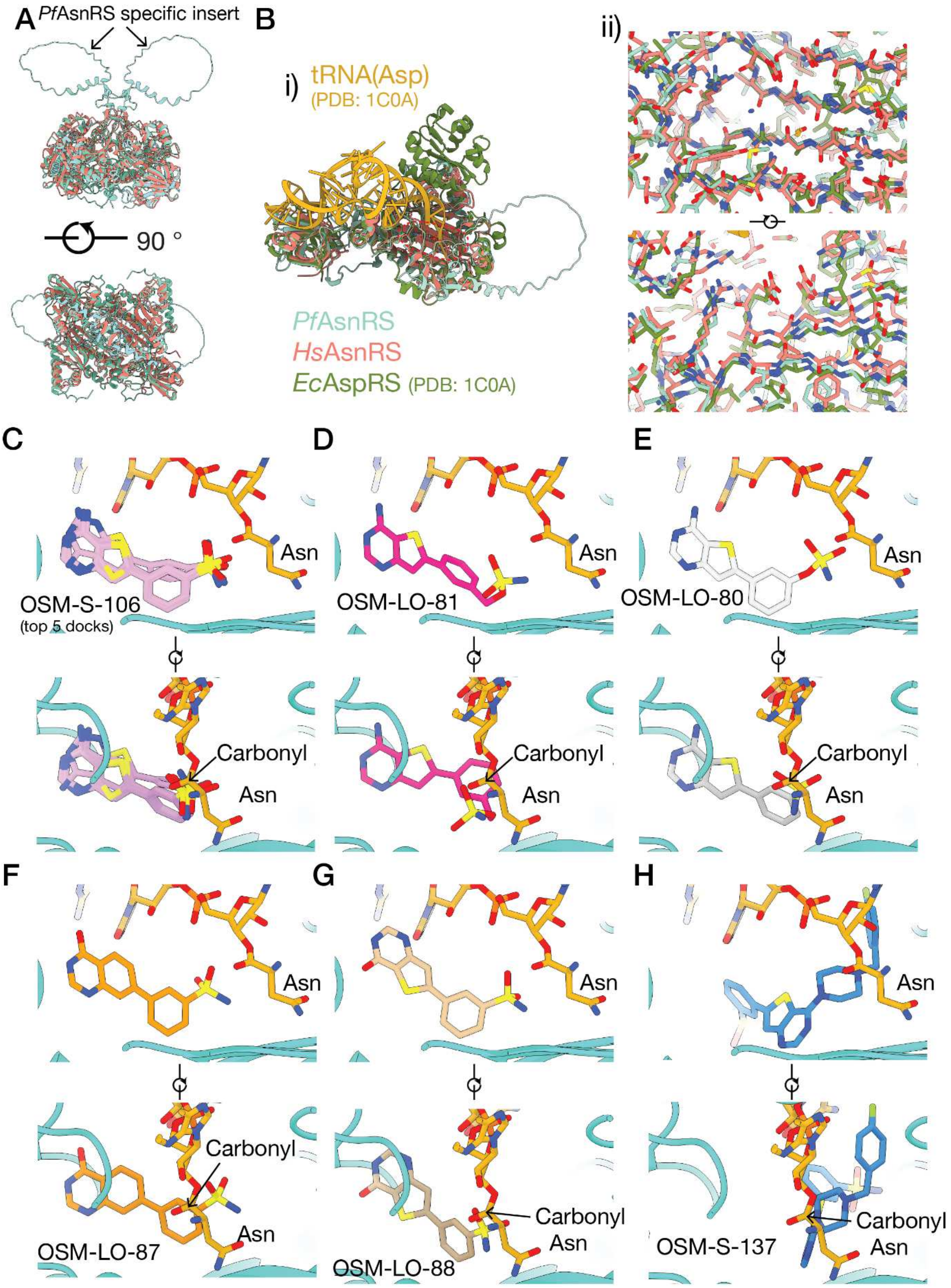

Figure S11. The *Pf*AsnRS-tRNA(Asn) complex and compound docking

(**A**) Alignment of the crystal structure of the *Hs*AsnRS dimer (salmon) with the predicted model of the *Pf*AsnRS dimer (teal). (**B**) (i) Alignment of the crystal structure of the *Pf*AsnRS/tRNA(Asn) model monomer (teal) with the crystal structure of the *Hs*AsnRS dimer (salmon) and the *E. coli* AspRS/tRNA(Asp) complex (PDB ID 1C0A, (Eiler et al., 1999)). (ii) Zoom of the active site of the alignment show close superposition of the active site structure. (**C**) Overlay of the top five *in silico* docks of OSM-S-106 to the *Pf*AsnRS/tRNA(Asn) model illustrating rotation of the sulfonamide. (**D**)-(**H**) Representative *in silico* docks of compounds to the *Pf*AsnRS/tRNA(Asn) model for, (**D**) OSM-LO-81 (**E**) OSM-LO-80, (**F**) OSM-LO-87, (**G**) OSM-LO-88, and (**H**) OSM-S-137. Two orientations of each docked compound are shown to illustrate alignment of the reactive groups with the tRNA-Asn carbonyl carbon.

Supplementary Tables

Table S1. Physicochemical properties of OSM-S-106

Mass	306 Da
Solubility	pH 2: 50 – 100 μg/mL; pH 6.5: 12.5 – 25 μg/mL
Calculated logp/ ALogP	1.49/ 1.65
Number of rotatable bonds	2
Polar Surface Area/ TPSA	149/ 112 Å^2
Molecular Species	Neutral
Hydrogen bond acceptors/ donors	5/ 2
Ro5 Violations	0

Table S2. ADME properties of OSM-S-106

System	Degradation half-life (min)	Intrinsic clearance (CL) (μL/min/mg protein)
Human microsomes	395 / 619	3.5 / 2.2
Mouse microsomes	19.7 / 20.4	70.5 / 68.1

Table S3. Changes in bulk recrudescent culture sensitivity to DSM265 following evolution of resistance. Two recrudescent wells (G7 and F3) showed 3.1 × IC_{50}, and 2.5 × IC_{50} shifts, respectively.

	**Dd2-B2 (parent)**	**DSM265_G7**	**DSM265_F3**
**Mean IC_{50} (nM) $\pm$ SEM (N)**	22.6 $\pm$ 0.9 (3)	71.0 $\pm$ 1.8 (3)	56.6 $\pm$ 3.8 (3)
**Fold Shift**	N/A	3.1	2.5

Table S4. Sequencing statistics for DSM265-selected parasites subjected to whole-genome sequencing. Whole-genome sequencing revealed a CNV amplification of ~2× within a 438 kb segment on chromosome 6 in G7, and a CNV amplification of ~1.4× within a 36 kb segment, also on chromosome 6, in one clone (Supp Dataset 2). Both segments contain *Pf*DHODH, the amplification of which is known to mediate low-level resistance to DSM265.

		**DSM265 treated clones**		**Parent**
**Sample names**		**F3**	**G7**	**Dd2_B2_Parent**
**Total reads**		4,542,517	2,914,074	4,680,824
**# Mapped reads**		3,803,690	2,703,481	4,342,594
**Duplication rate**		28.05%	20.01%	31.51%
**General error rate**		1.99%	2.08%	1.71%
**Mean mapping quality (Phred)**		56.47	56.33	56.74
**Depth of coverage**	**mean**	28.60	21.45	40.85
	**SD**	33.49	37.34	37.53
**% of PF genome with > x no. reads**	**1X**	96.15%	96.01%	96.07%
	**5X**	93.94%	92.65%	94.20%
	**10X**	90.83%	85.51%	91.81%

	**20X**	75.98%	55.19%	
	**30X**	46.59%	17.49%	71.42%

Table S5. Sequencing statistics for ramp-up OSM-106-selected parasites subjected to whole genome sequencing.

**Sample Name**	**Mean Whole Genome Coverage (x)**	**Number of Reads Aligned to 3D7 Reference**	**Proportion of Total Reads Aligned to 3D7 Reference**
Dd2-B2-Parent-11-2018	98.94	27645387	0.937
Dd2-OSM-S-106-3-E5	107.19	30413089	0.984
Dd2-OSM-S-106-3-H7	95.07	26571029	0.984
Dd2-B2-Parent-12-2020	136.19	47589603	0.978
OSM-S-106-Dd2-2A6	146.06	56424362	0.981
OSM-S-106-Dd2-2A9	153.35	52393477	0.978
OSM-S-106-Dd2-2B2	168.67	58309916	0.977
OSM-S-106-Dd2-2D6	122.4	45467320	0.983

Table S6. Activity of OSM-S-106 and control compounds against CRISPR-edited *Pf*AsnRS$_{R487S}$ mutant lines. The data points represent the mean ± S.D. from five independent experiments.

**CRISPR-edited *Pf*AsnRS$_{R487S}$ mutant lines**		
**Parasite line**	**Dd2 parent**	***Pf*AsnRS$_{R487S}$ mutant lines**
IC_{50} (μM)	0.071 ± 0.008	0.165 ± 0.016

Table S7. Activity of OSM-S-106 and against knockdown lines. n = Number of biological repeats, each of which has three technical repeats. The data points represent the mean ± SEM (where available).

**Knockdown line**	**Mean IC_{50} (μM) with 500 nM aTc**	**Mean IC_{50} (μM) with low (1 or 1.5 nM)/ no aTc**	**Average fold-shift**
*Pf*AsnRS	0.14 ± 0.02 (n = 3)	0.026 ± 0.006 (n = 3)	6.6
*Pf*AlaRS	0.078/ 0.100 (n = 2)	0.057/ 0.046 (n = 2)	1.8
*Pf*GlyRS	0.039/ 0.037 (n = 2)	0.040/ 0.041 (n = 2)	1.0
*Pf*NT4	0.021 ± 0.001 (n = 3)	0.007 ± 0.001 (n = 3)	3.0
*Pf*GDH3	0.040 ± 0.010 (n = 4)	0.046 ± 0.015 (n = 4)	1.0
*Pf*CA	0.033 ± 0.003 (n = 3)	0.026 ± 0.002 (n = 3)	1.2

Table S8. Crystallographic data collection and refinement statistics.

	***Hs*AsnRS-Apo**	***Hs*AsnRS/AMP-Asn**	***Hs*AsnRS-AMS**	***Hs*AsnRS-OSM106**
**Data collection**				
Space group	$P2_12_12_1$	$P2_12_12_1$	$P2_12_12_1$	$P2_12_12_1$
Wavelength (Å)	0.95373	0.9796	0.95373	0.95373
Number of images	1800	180	1800	1800
Oscillation range per image (˚)	0.1	1.0	0.1	0.1
Detector	Eiger 16M	Q315r (PAL BL5C)	Eiger 16M	Eiger 16M
Cell dimensions				
a, *b*, *c* (Å)	113.49, 127.19, 163.16	115.42, 125.84, 161.13	115.85, 126.29, 160.60	115.09, 126.81, 161.14
α, β, γ (˚)	90, 90, 90	90, 90, 90	90, 90, 90	90, 90, 90
Resolution range for data processing (Å)	49.05-1.90 (1.93-1.90)	50.00-2.16 (2.20-2.16)	49.29-1.90 (1.93-1.90)	49.46-2.00 (2.03-2.00)
R_{sym}†	0.108 (2.021)	0.160 (1.069)	0.176 (2.916)	0.195 (2.522)
R_{meas}§	0.117 (2.426)	0.172 (1.149)	0.191 (3.152)	0.212 (2.718)
R_{pim}‡	0.044 (0.921)	0.063 (0.420)	0.072 (1.186)	0.080 (1.035)
$CC_{1/2}$	0.99 (0.513)	0.993 (0.868)	0.998 (0.502)	0.997 (0.471)
$I/\sigma(I)$	9.5 (0.8)	12.3 (2.1)	7.5 (0.7)	6.4 (0.8)
Total observations	1279483 (60343)	931105	1277523 (62399)	1093766 (52694)
Unique reflections	185343 (8986)	126122 (6260)	185028 (9037)	159099 (7783)
Completeness (%)	99.8 (98.7)	100.0 (100.0)	99.9 (99.3)	99.9 (99.7)
Multiplicity	6.9 (6.7)	7.4 (7.4)	6.9 (6.9)	6.9 (6.8)
Wilson *B* factor (Å^2)	32.195	30.8	27.52	32.58
**Refinement**				
Resolution (Å)	46.58-1.90 (1.968-1.90)	49.88-2.16 (2.24-2.16)	49.29-1.90 (1.968-1.90)	49.46-2.0 (2.071-2.0)
Reflections used in refinement	185022 (18240)	125294 (12332)	184540 (18148)	158766 (15664)
R_{free} reflections	9064 (873)	6229 (646)	9326 (914)	7948 (778)
R_{work}	0.1858 (0.3803)	0.203 (0.278)	0.1809 (0.3426)	0.1818 (0.3149)
R_{free}	0.2211 (0.4071)	0.237 (0.321)	0.2172 (0.3730)	0.2208 (0.3352)
Protein molecules in asymmetric unit	4	4	4	4
Total nonhydrogen atoms	15123	14571	15820	15453
Protein	13884	13783	14007	14076
Ligand/ion	134	42	304	410
Solvent	1105	746	1589	1030
Mean B *factor (Å^2)*	44.91	39.86	37.02	41.33
Protein	44.72	39.86	36.37	41.05
Ligand/ion	54.98	42.58	35.50	48.66
RMS deviations				
Bond lengths (Å) (outliers >4σ)	0.013 (1)	0.003 (0)	0.007 (0)	0.007 (0)
Bond angles (°) (outliers >4σ)	1.172 (2)	0.58 (0)	0.861 (0)	0.873 (0)
Rotamer outliers	0.72	0.63	0.46	0.65
Clashscore	2.81	3.51	3.70	3.45

Cβ outliers	0	0	0	0
Molprobity score	1.09	1.34	1.41	1.35
Ramachandran Plot				
Favoured (%)	97.92	96.82	96.28	96.63
Allowed (%)	1.78	3.18	3.72	3.31
Outliers (%)	0.30	0	0	0.06
PDB code	8TC7	8H52	8TC8	8TC9

† $R_{\mathrm{sym}} = \Sigma_{hkl}\Sigma_i \, |I_i\,(hkl) \quad \langle I(hkl)\rangle \quad | / \Sigma_{hkl}\Sigma_i I_i\,(hkl)$

§ $R_{\mathrm{meas}} = \Sigma_{hkl}\,[N/(N\ 1)]^{½}\,\Sigma_i\,|I_i\,(hkl) \quad \langle I\,(hkl)\rangle \quad | / \Sigma_{hkl}\,\Sigma_i\,I_i\,(hkl)$

‡ $R_{\mathrm{pim}} = \Sigma_{hkl}\,[1/(N\ 1)]^{½}\,\Sigma_i\,|I_i\,(hkl) \quad \langle I\,(hkl)\rangle \quad | / \Sigma_{hkl}\,\Sigma_i\,I_i\,(hkl)$

$CC_{1/2}$ = Pearson correlation coefficient between independently merged half datasets

Table S9. List of oligonucleotides for gene knockdown donor vector construction.

Description	Nucleotide sequence
AsnRS cKD RHR forward	gtacggtacaaacccggaattcgagctcggATGTGTATGCCCTTATAATTTACAT
AsnRS cKD RHR reverse	aagacgagagattgggtattagacctagggataacagggtaatGACAACATGCAAGACAAAGAACTCTTTG
AsnRS sgRNA target site	CCACATGCAGGATTTGGTTT
AlaRS cKD RHR forward	gtacggtacaaacccggaattcgagctcggGCCATATACATTGTTTAGCTAATTAAC
AlaRS cKD RHR reverse	aagacgagagattgggtattagacctagggataacagggtaatCACATGAACAACGATCGAATA
AlaRS sgRNA target site	GTCACTAATGTGATAGGTGT
GlyRS cKD RHR forward	gtacggtacaaacccggaattcgagctcggCCATGTATGTATATTTTTACGTATG
GlyRS cKD RHR reverse	aagacgagagattgggtattagacctagggataacagggtaatTTCATCACAAGAACAAGTCTGATCA
GlyRS sgRNA target site	AAAGGGTATACCGATTTCAT
NT4 cKD RHR forward	gtacggtacaaacccggaattcgagctcggGGTTTTGTAATGCAATAATTCATTTGTAATTTGAAC
NT4 cKD RHR reverse	aagacgagagattgggtattagacctagggataacagggtaatGAAAAATATACCCTGTACCTTTTATATTCCTTC
NT4 sgRNA target site	GAAATATAAGATCACTGAGT
GDH3 cKD RHR forward	gtacggtacaaacccggaattcgagctcggCATTATATATTGCAGTACTCCCATATGTTG
GDH3 cKD RHR reverse	aagacgagagattgggtattagacctagggataacagggtaatCTGGATACACATCATCAAGTTACTC
GDH3 sgRNA target site	ATGATGATGACTATTATCCT
CA cKD RHR forward	gtacggtacaaacccggaattcgagctcggGATAAATACGATTAATTAGGTTTG
CA cKD RHR reverse	aagacgagagattgggtattagacctagggataacagggtaatTGGGTCTACAAAAAAAACTG
CA sgRNA target site	GTATCAAGTGCAGTTCACGT

(RHR, right homology region; cKD, conditional knockdown

Biological Methods

Activity against P. falciparum cultures.

Antimalarial activity against *P. falciparum* 3D7 was tested by TCGLS, Kolkata, India, using the lactate dehydrogenase (*Pf*LDH) growth inhibition assay as previously described (Gamo et al., 2010). Briefly, 70 µL of freshly prepared reaction mix containing 143 mM sodium L-lactate, 143 µM 3-acetyl pyridine adenine dinucleotide (APAD), 178.75 µM Nitro Blue tetrazolium chloride (NBT), diaphorase (2.83U/ml), 0.7% Tween 20, 100mM Tris-HCl pH 8.0 was added into each well of the incubation plate. Plates were shaken to ensure mixing and were placed in the dark at 21°C for 20 min. Data were normalized to percent growth inhibition

with respect to positive (0.2% DMSO, 0% inhibition) and negative (mixture of 100 μM chloroquine and 100 μM atovaquone, 100% inhibition) controls. *P. falciparum* strain (3D7) was obtained from BEI Resources.

Alternatively, sorbitol-synchronsied parasites (3D7 strain, ring stage) (Lawrence et al., 2000) were incubated with OSM-S-106 and other inhibitors for 72 h. Viability was assessed in the second cycle by flow cytometry, following labelling with 2 μM Syto-61 (Thermo Fisher Scientific) (Dogovski et al., 2015; Xie et al., 2014). Viability represents the parasitemia normalized to untreated and "kill" controls that were treated with 2 μM dihydroartemisinin (DHA; Sigma-Aldrich) for 48-72 h. For drug pulse assays, tightly synchronized Cam3.II-rev (Straimer et al., 2015) parasites (1-1.5% parasitemia, 0.2% final hematocrit) were added to the plates and incubated for 6 h. Drugs were removed and the parasitemia assessed in the trophozoite stage of the next cycle.

Activity against HepG2 and P. berghei
Hepatic human transformed cells (12 x 10^3; HepG2-A16-CD81-EGFP), stably transformed to express a GFP-CD81 fusion), were pretreated for 18 h with decreasing concentrations of the compounds of interest, over the range 50 μM to 0.85 nM. The cells were then infected with freshly dissected luciferase-expressing *P. berghei (PbLuc*) (4 x 10^3) sporozoites, as previously described (Swann et al., 2016). After 48 h of incubation with the compound, the viability of *P. berghei* exoerythrocytic forms (EEF) was measured by bioluminescence using Bright Glow reagent (Promega). HepG2 cytotoxicity was assessed by adding CellTiterGlo reagent (Promega). The plates were read in a PHERAstar FSX reader (BMG LABTECH).

Metabolic stability study using human liver microsomes
A solution of the test compounds in phosphate buffer solution (1 μM) was incubated in pooled human liver microsomes (0.5 mg/mL) for 0, 5, 20, 30, 45 and 60 minutes at 37°C in the presence and absence of an NADPH regeneration system (NRS). The reaction was terminated with the addition of ice-cold acetonitrile, containing a system suitability standard, at designated time points. The sample was centrifuged (4200 rpm) for 20 minutes at 20°C and the supernatant was diluted by half in water and then analysed by LC-MS/MS. The % parent compound remaining, half-life ($T_{1/2}$) and clearance ($CL_{int,app}$) were calculated using standard methodology. The experiment was carried out in duplicate. Verapamil, diltiazem, phenacetin and imipramine were used as reference standards.

Minimum inoculum of resistance
Minimum Inoculum of Resistance (MIR) studies were conducted for OSM-S-106 using a modified "Gate Keeper assay" (Duffey et al., 2021). The IC_{50} was determined to be 88.9 nM (N,n=3,2), and the IC_{90} was determined to be 169.2 nM (N,n=3,2) in the *P. falciparum* Dd2-B2 clone. A single-step selection was set up by exposing *P. falciparum* cultures (Dd2-B2, 3% hematocrit; 1E7 Dd2-B2 parasites in each well of a 24-well plate) to 3 x IC_{90} (507.7 nM) of OSM-S-106 over 60 days. Wells were monitored daily by smear during the first seven days to ensure parasite clearance, during which media was changed daily. Thereafter, cultures were screened three times weekly by flow cytometry and smearing, and the selection maintained a consistent drug pressure of 3 x IC_{90} over 60 days. No recrudescence was observed over the course of this selection. Control selections with DSM265 (at 58 nM, corresponding to $5\times IC_{50}$), yielded 14/96 recrudescent wells, consistent with earlier reports (Phillips et al., 2015) (Duffey et al., 2021). Whole-genome sequencing analysis employed MiSeq data from libraries of 2 x 300 bp paired end reads (Murithi et al., 2021).

In vitro evolution of P. falciparum with reduced sensitivity to OSM-S-106.
P. falciparum Dd2 was selected for resistance to OSM-106 over a period of two months, starting at the IC_{50} and increasing to 4x IC_{50}. Two independent selections were performed, and two or four clones were isolated from each of the selection flasks by limiting dilution, yielding a total of 6 resistant Dd2 clones. Whole-genome sequencing was applied to an average coverage of 117. Reads were mapped to the 3D7 reference genome. Mutations that were present in both the resistant clones and their isogenic parent were removed. In

addition, the genomes were analysed for potential copy number variation with the GATK4 CNV pipeline using panels of controls developed for the Dd2 genetic background (McKenna et al., 2010) (Miles et al., 2016).

Whole genome sequencing and analysis of OSM-S-106-resistant parasites

The sequencing library for parasite genomic DNA was prepared with the Nextera XT kit (Cat. No. FC-131-1024, Illumina) following the standard dual index protocol. The library was sequenced at the UC San Diego IGM Genomics Center on the Illumina HiSeq 2500 in RapidRun mode to generate 100bp paired-end reads. Fastq files were aligned to the *P. falciparum* 3D7 reference genome (PlasmoDB v13.0) using the previously described Platypus pipeline (Manary et al., 2014). The seven clones generated in the study (one parent clone and six OSM-S-106-resistant clones) were sequenced to an average depth of 132x.

SNVs and INDELs were called against the 3D7 reference genome using GATK HaplotypeCaller and filtered according to GATK recommendations (McKenna et al., 2010). Briefly, SNVs were retained if they met the following filter criteria: ReadPosRankSum >8.0 or <-8.0, QUAL<500, Quality by Depth (QD) <2.0, Mapping Quality Rank Sum <-12.5, and filtered depth (DP) <7. INDELs were retained if they passed ReadPosRankSum <-20, QUAL<500, QD<2, and DP<7. SnpEff was used to annotate variants in the resulting VCF file (Cingolani et al., 2012). Variants with passing quality metrics and ≥90% allele frequency were further filtered to remove mutations that were also present in the Dd2 parent clone, as these would not have evolved over the course of OSM-S-106 selection. Each resistant clone contained 3-6 SNVs or INDELs that met all filtering criteria. CNVs were identified by differential Log2 copy ratio as previously described (Summers et al., 2022).

*Generation of Pf*AsnRS$_{R487S}$ *transfectant cell line*

A single CRISPR/Cas9 plasmid was used to generate parasites encoding the R487S mutation in *Pf*AsnRS, as shown in Suppl Figure 3. Two guide RNAs were designed using Benchling (benchling.com). The gRNA1 (5'-CATTCGAAGTGAAAGTTGAA-3') and gRNA2 (AGTGAAAGTTGAATGGGGAA) were located near the mutation site. Both gRNAs and their complementary sequences were synthesized as primers by IDT. Each gRNA was cloned into the pDC2-coCas9-gRNA plasmid essentially as described previously (Adjalley and Lee, 2022). A donor template of 780 bp, encompassing coding nucleotide sequences 1045-1824, was synthesized (Thermo Fisher Scientific) and assembled at the *Aat*II and *Eco*RI sites using NEBuilder HiFi DNA Assembly. In addition to the R487S mutation, additional silent shield mutations that prevent Cas9 binding were introduced, as shown in Suppl Figure 3. Transfections were performed on ring-stage Dd2 parasites using a BioRad Gene Pulser II as described (Adjalley and Lee, 2022), with 5 nM WR99210 drug pressure applied for 8 days. Edited clones were isolated by limiting dilution and validated by Sanger sequencing.

Generation of conditional knockdown parasite lines

Conditional knockdown (cKD) *P. falciparum* lines were generated for the cytosolic AsnRS (PF3D7_0211800), cytosolic AlaRS (PF3D7_1367700), cytosolic GlyRS (GlyRS; PF3D7_1420400), *Pf*NT4 (PF3D7_0103200), *P. falciparum* glutamate dehydrogenase (GDH3; PF3D7_0802000), and *P. falciparum* carbonic anhydrase (CA, PF3D7_1140000) by fusing the coding sequences and non-coding RNA aptamer sequences in the 3'-UTR, permitting translation regulation using the TetR-DOZI system (Ganesan et al., 2016; Nasamu et al., 2021). Gene editing was achieved by CRISPR/*Sp*Cas9 using the linear pSN054 vector that contains cloning sites for the left homology region (LHR) and the right homology region (RHR) as well a target-specific guide RNA under control of the *T7* promoter. Cloning into the pSN054 donor vector was carried out following previously described procedures (Ganesan et al., 2016; Nasamu et al., 2021). The vector includes V5-2xHA epitope tags, a 10x tandem array of TetR aptamers upstream of an *Hsp86* 3'UTR, and a multicistronic cassette for expression of TetR-DOZI (translation regulation), *blasticidin S-deaminase* (selection marker) and a *Renilla luciferase* (*RLuc*) reporter. All primer and synthetic fragment sequences that were generated using the BioXP™ system and IDT gBlocks™ are included in Supplementary Table S9. The final constructs were sequence-verified and further confirmed by restriction digests.

Transfection into Cas9- and T7 RNA polymerase-expressing NF54 parasites was carried out by pre-loading erythrocytes with the donor vector as previously described (Deitsch et al., 2001). Parasite cultures were maintained continuously in 500 nM anhydrotetracycline (aTc, Sigma-Aldrich 37919) and drug selection with 2.5 µg/mL of Blasticidin S (RPI Corp B12150-0.1) was initiated four days after transfection. Cultures were monitored by Giemsa smears and RLuc measurements.

Growth assay for knockdown parasite lines

Assessment of parasite viability during target protein perturbations were carried out using luminescence as a readout of growth. Synchronous ring-stage parasites, cultured in the presence (50 nM) and absence of aTc, were set up in triplicate in a 96-well U-bottom plates (Corning® 62406-121). Luminescence signals were taken at 0 and 72 h post-invasion using the Renilla-Glo(R) Luciferase Assay System (Promega E2750) and the GloMax® Discover Multimode Microplate Reader (Promega). The luminescence values in the knockdown conditions were normalized to aTc-treated (100% growth) and dihydroartemisinin-treated (500 nM, no growth) samples and results were visualized using GraphPad Prism (version 9; GraphPad Software).

OSM-S-106 susceptibility assays for knockdown parasite lines

The stock solution of OSM-S-106 was dispensed into 96-well (BD Falcon™ 62406-121) and 384-well (Corning® MPA-3656) U-bottom microplates and serially diluted in complete medium to yield a final concentration in the assay ranging from 0.8-0.003 μM. Synchronous ring-stage *Pf*AsnRS, *Pf*AlaRS, *Pf*GlyRS, *Pf*NT4, *Pf*GDH3, and *Pf*CA cKD parasites, as well as a control line expressing a fluorescent protein under the control of the TetR/DOZI module (Ganesan et al., 2016), were maintained in 0.5 μM aTc to achieve wild-type protein levels, and 0.001 or 0.0015 µM aTc for knockdown of *Pf*AsnRS, *Pf*AlaRS and *Pf*GlyRS, and no aTc for knockdown of *Pf*NT4, *Pf*GDH3 and *Pf*CA. DMSO- and dihydroartemisinin-treatment (0.5 μM) served as reference controls. Luminescence was measured after 72 h as described above and IC_{50} values were obtained from corrected dose-response curves using GraphPad Prism.

Protein translation assay.

Highly synchronous *P. falciparum* Cam3.II^{rev} (Straimer et al., 2015) infected RBCs (30-35 h post-invasion) were exposed to OSM-S-106, cycloheximide, and WR99210 for 4 h. O-propargyl-puromycin (OPP) (Abcam) was added to the culture and incubated for a further 2 hr. Parasites were washed three times in 1x PBS (Gibco™) and fixed with 4% formaldehyde (Polysciences) and 0.02% glutaraldehyde (Sigma) in 1x PBS for 20 min at room temperature (RT). Cells were washed two times with buffer A (3% human serum in 1x PBS). Pellets were permeabilized in buffer A containing 0.05% Triton® X-100 and washed two times with buffer A. Fixed-permeabilized cells were subjected to copper-catalyzed azide–alkyne cycloaddition (CuAAC) at 37°C for 1 h in the presence of 0.1 mM $CuSO_4$, 0.5 mM THPTA, 5 mM sodium ascorbate, and 0.1 µM Alexa Fluor 488 azide in buffer A. Pellets were washed four times in buffer A and resuspended in buffer A containing 25 µg/ml propidium iodide (Invitrogen ™). Cells were interrogated by flow cytometry (FACS Canto II; BD Biosciences, San Jose, CA) using FITC and Cy™5.5 channels.

Western blotting analysis of eIF2α phosphorylation

Highly synchronous *P. falciparum* Cam3.II^{rev} infected RBCs (30-35 h post-invasion; 2.5% hematocrit, 5-6% parasitemia) were exposed to OSM-S-106, OSM-S-137, borrelidin (Sigma) or 0.05% DMSO (mock) for 3 h. Infected RBCs were pelleted, washed with ice-cold 1x PBS + cOmplete™ EDTA-free protease inhibitor cocktail (Roche), and lysed with 0.03% saponin in 1x PBS on ice. Parasite pellets were washed three times with 1x PBS + cOmplete™ EDTA-free protease inhibitor (Roche) cocktail and centrifuged at 13,000 *x g* for 10 min. The pellets were solubilized in Bolt ™ LDS sample buffer containing reducing agent (Invitrogen™), vortexed at RT for 5 min, and boiled at 95°C for 5 min. Samples were resolved by SDS-PAGE on Nupage™ 4-12% Bis-Tris acrylamide gel at 150 V for 50 min and transferred to nitrocellulose membranes using iBlot ™ 2 (Life Technologies). Membranes were blocked in PBST (5% (w/v) skim milk in PBS) for 1 h at RT, probed with primary antibodies at 4°C overnight, and with secondary antibodies at RT for 1 h. Primary antibodies: rabbit

anti-phospho-eIF2α (Cell Signaling Technology-119A11; 1:1000); polyclonal mouse anti-*Pf*BiP (WEHI; 1:1000). Secondary antibodies: goat anti-rabbit IgG-HRP (Chemicon-AP132P;1:20,000); goat anti-mouse IgG-HRP (Chemicon-AP181P; 1:50,000). The membranes were washed and incubated with Clarity Western ECL Substrate (Bio-Rad) and imaged using the ChemiDoc ™ MP imaging system (Bio-Rad).

Mass spectrometry to identify and quantify the OSM-S-106-aspargine conjugate
In vitro AsnRS reactions were set up with the following components: 1 µM *Pf*AsnRS, 20 µM L-asparagine, 10 µM ATP, 10 µM OSM-S-106 and 2.5 mg/mL *E. coli* tRNA (Merck). The reaction buffer consists of 100 mM HEPES pH 7.5 (KOH), 160 mM KCl, 3.5 mM $MgCl_2$, 1 mM DTT. The mixture was incubated at 37°C for 1 h. After that, an equal volume of 8 M urea was added to the mixture. Finally, trifluoroacetic acid was added to a final concentration of 1%. The sample was centrifuged at 15,000 g for 10 min and the supernatant was used for LCMS analysis. Synthetic Asn-OSM-S-106 standards were processed in the same way.

For identification of conjugates in cell cultures, a late trophozoite stage *P. falciparum* (3D7 strain) culture was exposed to 1 µM or 10 µM OSM-S-106 for 3 h. Following drug treatment, parasite-infected RBCs were lysed with 0.1% saponin in PBS and the parasite pellet was washed 3 times with ice-cold PBS. Cell pellets were kept on ice and resuspended in water as one volume, followed by the addition of five volumes of cold chloroform-methanol (2:1 [vol/vol]) solution. Samples were incubated on ice for 5 min, subjected to vortex mixing for 1 min and centrifuged at 12,000 rpm for 10 min at 4°C to form 2 phases. The top aqueous layer was transferred to a new tube and subjected to LCMS analysis.

High-performance liquid chromatography (HPLC) and mass spectrometric (MS) analyses
Samples were analysed by reversed-phase ultra-high performance liquid chromatography (UHPLC) coupled to tandem mass spectrometry (MS/MS) employing a Vanquish UHPLC linked to an Orbitrap Fusion Lumos mass spectrometer (Thermo Fisher Scientific, San Jose, CA, USA) operated in positive ion mode. Solvent A was 0.1% formic acid/10 mM ammonium acetate in water and solvent B was 0.1% formic acid/10 mM ammonium acetate in acetonitrile. 10 µL of each sample was injected into an RRHD Eclipse Plus C18 column (2.1 × 1000 mm, 1.8 µm; Agilent Technologies, USA) at 50 °C at a flow rate of 350 µL/min for 3 min using 0% solvent B. During separation, the percentage of solvent B was increased from 0% to 25% in 7 min. Subsequently, the percentage of solvent B was increased to 99% in 0.1 min and then maintained at 99% for 0.9 min. Finally, the percentage of solvent B was decreased to 0% in 0.1 min and maintained for 3.9 min.
MS experiments were performed using a Heated Electrospray Ionization (HESI) source. The spray voltage, flow rate of sheath, auxiliary and sweep gases were 3.5kV, 20, 6, and 1 'arbitrary' unit(s), respectively. The ion transfer tube and vaporizer temperatures were maintained at 350°C and 400°C, respectively, and the S-Lens RF level was set at 50%. A full-scan MS spectrum and targeted MS/MS for proton adduct of Asn-OSM-S-106 or 20 possible common amino acid-containing inhibitor adducts were acquired in cycles throughout the run. The full-scan MS-spectra were acquired in the Orbitrap at a mass resolving power of 120,000 (at m/z 200) across an m/z range of 200–1500 using quadrupole isolation and the targeted MS/MS were acquired using higher-energy collisional dissociation (HCD)-MS/MS in the Orbitrap at a mass resolving power of 7500 (at m/z 200), a normalized collision energy (NCE) of 20% and an m/z isolation window of 1.6.

Analytical Ultracentrifugation
*Pf*AsnRS, *Pf*AsnRS$_{R478S}$, CD*Hs*AsnRS and *Hs*AsnRS samples were diluted to 2.8 µM in 25 mM Tris-HCl, pH 7.4, 150 mM NaCl and 0.5 mM TCEP. 400 µL aliquots were loaded into double-channel quartz window cells (Beckman Coulter), with the above buffer in the reference compartment. Cells were centrifuged at 50,000 rpm or 40,000 rpm at 20°C using an XL-I analytical ultracentrifuge (Beckman Coulter) or an Optima analytical ultracentrifuge (Beckman Coulter). Radial absorbance data were acquired at a wavelength of 238 or 280 nm (as indicated), with radial increments of 0.003 cm, in continuous scanning mode. The sedimenting boundaries were fitted to a model that describes the sedimentation of a distribution of sedimentation coefficients with no assumption of heterogeneity (c(s)) using the program SEDFIT (Schuck and Rossmanith,

2000). Data were fitted using a regularization parameter of p = 0.95, floating frictional ratios, and 250 sedimentation coefficient increments.

ATP consumption assay
The consumption of ATP by wildtype *Pf*AsnRS, *Pf*AsnRS$_{R487S}$ and *Hs*AsnRS was determined using a luciferase-based assay as per the manufacturer's instructions (Kinase-Glo Luminescent Kinase Assay, Promega). Reactions were conducted in 100 mM HEPES pH 7.5, 160 mM KCl, 3.5 mM $MgCl_2$, 0.1 mg/mL BSA, 1 mM DTT, with 200 μM L-asparagine, 10 μM ATP, 1 unit/mL inorganic pyrophosphatase and 2.5 mg/mL *E.coli* tRNA if present. Enzyme concentration and incubation time for each experiment are described in the figure legends. Reactions were incubated at 37°C, followed by addition of the Kinase Glo reagent and incubation for 10 minutes at room temperature. Luminescence output was measured using a Clariostar plate reader, and the concentration of ATP quantified by linear regression using an ATP standard curve (Microsoft Excel). Data are normalised to the ATP consumption by DMSO (0.5%) treated AsnRSs as a positive control (100% activity). Samples with no enzyme served as negative controls. Dose-response curves and IC50 values were obtained using GraphPad Prism.

Expression and purification of His tagged *human* AsnRS *catalytic domain* (His-CD*Hs*AsnRS).
The expression and purification of His tagged human AsnRS catalytic domain, residues A98–P548 (His-CD*Hs*AsnRS) has been described previously (Park et al., 2018). Briefly, the amino acid sequence comprising residues A98-P548 with N-terminal His$_6$-tag was expressed via pET-28a in *E. coli* strain Solu_BL21 (Genlantis). Cells were cultivated in 1 L LB media supplemented with 50 μg/mL ampicillin in a shaker-incubator at 37°C to OD_{600} 0.5. Recombinant protein expression was induced by addition of 0.5 mM isopropyl β-D-1-thiogalactopyranoside (IPTG). Cultures was further incubated for 4 h at 37°C and cells harvested by centrifugation (6,000 g). Pelleted cells were resuspended in lysis buffer containing 0.5 M NaCl, 20 mM Tris-HCl (pH 7.5), 35 mM imidazole, and 1 mM β-mercaptoethanol, lysed with an ultrasonic processor (Cole-Parmer), and centrifuged at 35,000 ×g for 30 min. The supernatant was filtered with 0.45-μm syringe filter device (Sartorius) and loaded onto a HisTrap chelating 5-mL HP column (Cytiva). The loaded column was washed with lysis buffer, and retained His-CD*Hs*AsnRS was eluted with an increasing gradient of lysis buffer containing 1 M imidazole. Prior to ion-exchange chromatography, fractions containing CD*Hs*AsnRS were buffer-exchanged with binding buffer; 100 mM NaCl, 20 mM Tris-HCl pH 7.5, and 5 mM dithiothreitol using a HiPrep desalting 26/10 column (Cytiva) and loaded onto a HiTrap Q 5-mL HP column (Cytiva). CD*Hs*AsnRS was eluted with an increasing gradient of binding buffer containing 1 M NaCl and finally subjected to a HiLoad 16/600 Superdex 200 pg column (Cytiva) equilibrated with the buffer containing 200 mM NaCl, 10 mM HEPES-NaOH (pH 7.0).

Expression and purification of native PfAsnRS, PfAsnRS$_{R478S}$, HsAsnRS and CDHsAsnRS recombinant proteins.
Plasmid vectors were designed to express recombinant *Pf*AsnRS, *Pf*AsnRS$_{R478S}$, *Hs*AsnRS (residues M1–P548) and CD*Hs*AsnRS (residues A98–P548) comprising a hexa-histidine tag at the N-terminus, an intervening TEV cleavage sequence and C-terminal AsnRS sequence (His-TEV-AsnRS). Open reading frames were codon optimized for expression in *E. coli*, synthesized and cloned into the pET11a expression vector (GeneScript). *E. coli* BL21(DE3) containing expression vector was cultivated in 2 L LB media containing 100 μg/mL ampicillin in a shaker-incubator at 37°C. The culture was transferred to a 16°C shaker-incubator when the cell density approached mid log phase (OD_{600}~0.6). Recombinant His-TEV-AsnRS expression was induced by addition of 0.1 mM IPTG to culture media and cells incubated for an additional 16 hours. Cells were harvested by centrifugation (6,000 g) and resuspended in 40 mL lysis buffer containing 50 mM Tris-HCl, pH 7.4, 350 mM NaCl, 40 mM imidazole, 0.5 mM TCEP, 1 mg/mL lysozyme and 1x protease inhibitor cocktail (Roche). Cells were lysed by sonication (Microtip, QSonica) and the lysate clarified by centrifugation at 30,000 g for 25 min at 4 °C and passage through 0.8/0.2 μm (Pall) syringe filter. The supernatant was applied to a 5 mL HisTrap HP column (GE Healthcare) and washed with 50 mL binding buffer containing 50 mM Tris-HCl, pH 7.4, 350 mM NaCl, 40 mM imidazole, and 0.5 mM TCEP. His-TEV-AsnRS enzyme was eluted using a 0-500 mM

imidazole gradient in binding buffer over 100 mL. His-tagged TEV protease (L56V/S135G/S219V triple-mutant (Cabrita et al., 2007) was added to His-TEV-AsnRS (mass ratio 1:100, His-TEV-AsnRS:His-TEV protease) and dialyzed overnight at 4°C against 50 mM Tris-HCl, pH 7.4, 350 mM NaCl, 40 mM imidazole, 0.5 mM TCEP. The resultant native AsnRS enzyme was isolated from cleaved His tag and His-TEV protease by passage of sample through a 5 mL HisTrap HP column and collection of flow-through material. Native AsnRS enzyme was further purified by gel filtration using a HiLoad 16/600 Superdex 200 column (GE Healthcare), pre-equilibrated in 25 mM Tris, pH 7.4, 150 mM NaCl and 0.5 mM TCEP.

Crystallisation and X-ray diffraction data collection
For crystallization of Asn-AMP-bound CD*Hs*AsnRS, purified apo His-CD*Hs*AsnRS was concentrated to 10 mg/mL. Crystals of His-CD*Hs*AsnRS were first obtained with a solution containing 20% (v/v) glycerol, 40 mM potassium phosphate, and 16% (w/v) polyethylene glycol 8,000 using the hanging drop vapor diffusion method at 295 K. The drops containing crystals were mixed with their reservoir solutions supplemented with 10 mM adenylyl imidodiphosphate lithium salt hydrate, 10 mM L-asparagine, and 20 mM $MgCl_2$ with a 1:1 molar ratio. The crystals were further incubated for 8 h at 295 K, flash-cooled in a 100 K nitrogen stream, and subjected to X-ray diffraction. The collected data were processed with HKL2000. Initial phase estimates were obtained by molecular replacement with *PHASER* using the previous apo-His-CD*Hs*AsnRS structure (PDB ID: 5XIX) as a template. Automated structure refinement using phenix.refine (Adams et al., 2010) was followed iteratively by manual model building in *COOT* (Emsley et al., 2010). The statistics for the His-CD*Hs*AsnRS structure are shown in Supplementary Table 8.

For Asn-OSM-S-106 bound, Asn-AMS bound, and apo CD*Hs*AsnRS, purified apo CD*Hs*AsnRS (native sequence) was concentrated to 10 mg/mL. Crystals were obtained in a solution containing 20% (v/v) glycerol, 40 mM potassium phosphate and 14% polyethylene glycol 8,000, and 100-mM Tris pH 7.6 using the sitting drop vapour diffusion method at 295 K. Drops containing crystals were mixed with their reservoir solutions supplemented with 350 μM Asn-OSM-S-106 or 350 μM Asn-AMS. The crystals were further incubated for 24 h at 295K. Crystals were flash-cooled in liquid nitrogen directly from the crystallization drop, and X-ray diffraction data were collected at 100 K and a wavelength of 0.9537 Å using the Eiger 16M detector at the MX2 beamline of the Australian Synchrotron (Aragão et al., 2018). Diffraction data were indexed and integrated using XDS (Kabsch, 2010) and analysed using POINTLESS (Evans, 2011), prior to merging by *AIMLESS* (Evans and Murshudov, 2013) from the *CCP4* software suite (Winn et al., 2011). Initial phase estimates were obtained by molecular replacement in PHASER (Mccoy et al., 2007) using modified coordinates of our Asn-AMP-bound CD*Hs*AsnRS as the search model. Automated structure refinement using phenix.refine (Adams et al., 2010) was followed iteratively by manual model building in *COOT* (Emsley et al., 2010). Structure refinement was performed using translation/libration screw (TLS) refinement with each chain comprising a single TLS group. Restraints for Asn-OSM-S-106 and Asn-AMS were generated using phenix.elbow (Moriarty et al., 2009). Final data collection and refinement statistics are shown in Supplementary Table 8.

Modelling of the P. falciparum AsnRS/ Asn-tRNA complex
A model of the *Pf*AsnRS/ Asn-tRNA complex was generated by combining a modified version of the AlphaFold model for *Pf*AsnRS bound to Asn-AMP with the tRNA from the structure of the *E. coli* aspartyl-tRNA synthase/ tRNA complex, 1C0A (Eiler et al., 1999). The catalytic domain of the *Pf*AsnRS model was aligned to the equivalent region of 1C0A using PyMOL (Schrödinger, 2022) and visual inspection showed an extremely good match for the local structure, with the tRNA from 1C0A positioned appropriately across both the active site and onto the anticodon domain. The only significant clash was of the acceptor stem with residues of the flipping loop adjacent to the active site, due to the *Pf*AsnRS model having these in the closed conformation seen in the tRNA-free structures of class II tRNA synthase enzymes (Eiler et al., 1999). The conformation of the flipping loop in the *Pf*AsnRS model was manually corrected to the open position using Coot (Emsley et al., 2010), and the *Pf*AsnRS/ Asn-AMP / tRNA complex model was minimised to remove any minor steric

overlaps using SybylX2.1 (Certara, NJ, USA). To generate the *Pf*AsnRS/ AMP/ Asn- tRNA complex, the bond between the asparagine residue and AMP was manually broken and a new bond to the 3’OH oxygen of the acceptor stem terminal adenine was added using SybylX2.1. The modified complex was minimised to correct any errors in bond lengths or angles.

Chemical Methods

Chemical abbreviations

Asn: Asparagine amino acid
AMS: Acyl sulfonyladenosines
Boc = *tert*-Butoxycarbonyl
DCM: Dichloromethane
DIPEA = *N,N*-Diisopropylethylamine
DMA = *N,N*-Dimethylacetamide
DMAP: 4-Dimethylaminopyridine
DMF = *N,N*-Dimethylformamide
EDCl: 1-Ethyl-3-(3-dimethylaminopropyl)carbodiimide
Et: Ethyl group
HATU = 1-[bis(dimethylamino)methylene]-1H-1,2,3-triazolo[4,5-*b*]pyridinium 3-oxide hexafluorophosphate
LDA: Lithium diisopropylamide
TBAF = Tetrabutylammonium fluoride
TBSCl = *tert*-Butyldimethylsilyl chloride
TFA = Trifluoroacetic acid
THF = Tetrahydrofuran

General information 1

Reagents were purchased from Sigma-Aldrich, Merck, Fisher Scientific, Apollo Scientific, and Fluorochem and were used without further purification unless specified. Anhydrous conditions: glassware was dried at >130 °C for >12 h, assembled hot, and allowed to cool under a high vacuum or purged with inert gas where suitable. Nitrogen (N_2) and argon (Ar) gas were used as obtained, from a cylinder. On a Schlenk line, the phrase *in vacuo* equates to 10 mbar. Reduced pressure means 900 to 50 mbar under rotary evaporation at 40 °C. Davisil Grace Davison 40 – 63 µm (230 – 400 mesh) silica gel and a Biotage Isolera One or Biotage Selekt were used for automated flash chromatography. Analytical thin-layer chromatography (TLC) was carried out on Merck Silica Gel 60 F_{254}-precoated aluminum plates (0.2 mm) and observed using UV irradiation (254 nm and 280 nm) and staining with potassium permanganate, anisaldehyde, or ninhydrin. High-temperature reactions were carried out in either temperature-controlled silicone oil baths or DrySyn heating blocks.

Melting points (M.P.) were measured using a Stanford Research Systems OptiMelt instrument at 1 °C min^{-1} (capillaries = 1.5 – 1.6 mm, 90 mm). Without atmospheric adjustment, infrared spectroscopy was performed using a Bruker Alpha-E (attenuated total reflectance) and analysed with Microlab PC software. The samples were examined neat. Bruker spectrometers were used for nuclear magnetic resonance spectroscopy at 300 K: AVANCE III 400 (^{1}H at 400 MHz, ^{13}C at 101 MHz), or AVANCE III 500 (^{1}H at 500 MHz, ^{13}C at 126 MHz). Spectra were analysed with Mestrelab Research Mnova. Deuterated solvents ($CDCl_3$ and DMSO-d_6) were obtained from Sigma-Aldrich. ^{1}H and ^{13}C chemical shifts are reported in parts per million (ppm). Spectroscopic chemical shifts were calibrated to residual solvent peaks (^{1}H: $CHCl_3$ 7.26 ppm, dimethyl sulfoxide (DMSO) 2.50 ppm; ^{13}C: $CHCl_3$ 77.16 ppm, DMSO 39.52 ppm). The multiplicities are described as either a singlet (s), doublet (d), triplet (t), quartet (q), quintet (qn) or multiplet (m).

High resolution mass spectrometry was carried out on Agilent 6545XT AdvanceBio LC/Q-TOF with ESI ionisation. The charge of the ion specifies whether the detection is positive or negative; for instance, $[M+H]^+$ denotes positive-ion detection. Analytical liquid chromatography-mass spectrometry (LCMS) was performed on an Agilent Infinity 1290 II system consisting of a quaternary pump (G7111A) and a diode array detector WR (G7115A) coupled to a InfinityLab LC/MSD (G6125B) using ESI. An Agilent Poroshell 120 EC-C18 column (2.7 µm, 4.6 x 50 mm) was eluted at a flow rate of 1.5 mL/min with a mobile phase of 0.05 % formic acid in H_2O and 0.05 % formic acid in MeCN. Preparative LCMS was performed on a combined Agilent Infinity 1260 II and Infinity 1290 II system consisting of a preparative binary pump (G7161A) and a multiple wavelength detector (G7165A) coupled to a InfinityLab LC/MSD (G6125B) using ESI and a preparative open-bed fraction collector (G7159B). An Agilent Eclipse XDB-C18 column (5 µm, 9.4 x 250 mm) was eluted at a flow rate of 5 mL/min with a mobile phase of 0.05% formic acid in H_2O and 0.05% formic acid in MeCN. As determined by NMR spectroscopy, the purity of all substances exceeded 95%.

General information 2 (for Asn-AMS synthesis)

Reagents: Reagents were obtained from Aldrich Chemical (www.sigma-aldrich.com) or Acros Organics (www.fishersci.com) and used without further purification. Optima or HPLC grade solvents were obtained from Fisher Scientific (www.fishersci.com), degassed with Ar, and purified on a solvent drying system unless otherwise indicated.

Reactions: All reactions were performed in flame-dried glassware under positive Ar pressure with magnetic stirring unless otherwise noted. Liquid reagents and solutions were transferred through rubber septa via syringes flushed with Ar prior to use.Cold baths were generated as follows: 0 °C, wet ice/water; –10 °C, wet ice/brine; –20 °C, dry ice/isopropanol monitored with a thermometer; –44 °C, dry ice/CH_3CN; –63 °C, dry ice/chloroform; –78 °C, dry ice/acetone; –100 °C, dry ice/Et_2O.

Chromatography: TLC was performed on 0.25 mm E. Merck silica gel 60 F254 plates and visualized under UV light (254 nm) or by staining with potassium permanganate ($KMnO_4$), cerium ammonium molybdate (CAM), phosphomolybdic acid (PMA), iodine (I_2), or *p*-anisaldehyde. Silica flash chromatography was performed on E. Merck 230–400 mesh silica gel 60. Analytical to semi-preparative HPLC was carried out on a Waters Alliance 2695 HPLC with 2996 diode array detector with a Higgins Analytical Targa C18 reverse phase column (5 cm × 4.6 mm, 3 um, 120 Å), using a flow rate of 1.0 mL/min and a gradient of 5 – 95% CH_3CN in 0.1% aq TFA over 5 min with UV detection at 254 nm and ELSD detection.

Analytical instrument: NMR spectra were recorded on a Bruker UltraShield Plus 500 MHz Avance III NMR or UltraShield Plus 600 MHz Avance III NMR with DCH CryoProbe at 24 °C in $CDCl_3$ unless otherwise indicated. Chemical shifts are expressed in ppm relative to TMS (^1H, 0 ppm) or solvent signals: $CDCl_3$ (^{13}C, 77.0 ppm), C_6D_6 (^1H, 7.16 ppm; ^{13}C, 128.0 ppm) or acetone-d_6 (^{13}C, 206.2 ppm); coupling constants are expressed in Hz. NMR spectra were processed using Bruker TopSpin, Mnova (www.mestrelab.com/software/mnova-nmr), or nucleomatica iNMR (www.inmr.net) software. Mass spectra were obtained at the MSK Analytical Core Facility on a Waters Acuity SQD LC-MS or PE SCIEX API 100 by electrospray (ESI) ionization. High resolution mass spectra were obtained on a Waters Acuity Premiere XE TOF LC-MS by electrospray ionization (ESI).

Nomenclature: N.B.: Atom numbers in chemical structures herein refer to the standard nucleoside numbering system used in the text of the article and Supporting Information and not to IUPAC nomenclature, which was used solely to name each compound. Compounds not cited in the paper are numbered herein from S1.

General Synthetic Procedure

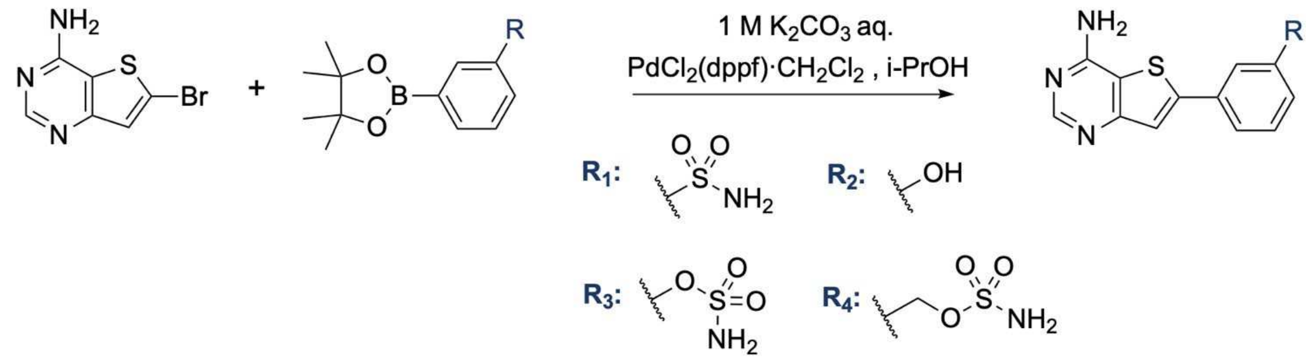

General procedure 1: Suzuki reaction between thienopyrimidine core and functionalised aryl halides.

Organohalide (1 equiv.), pinacol boronate (1.1 equiv.) and $PdCl_2$(dppf)·DCM (0.1 equiv.) were combined. *i*-PrOH and 1 M aq. K_2CO_3 (1 – 3 equiv. for the stated condition) were added and the reaction mixture was heated using conventional heating or microwave irradiation for the stated time after being degassed with Ar. The reaction mixture was diluted with MeOH, filtered through celite, and concentrated under reduced pressure to give a residue that was purified by automated flash chromatography on silica to give the coupled product.

Synthesis and Characterisation of Compounds

Synthesis of OSM-S-106

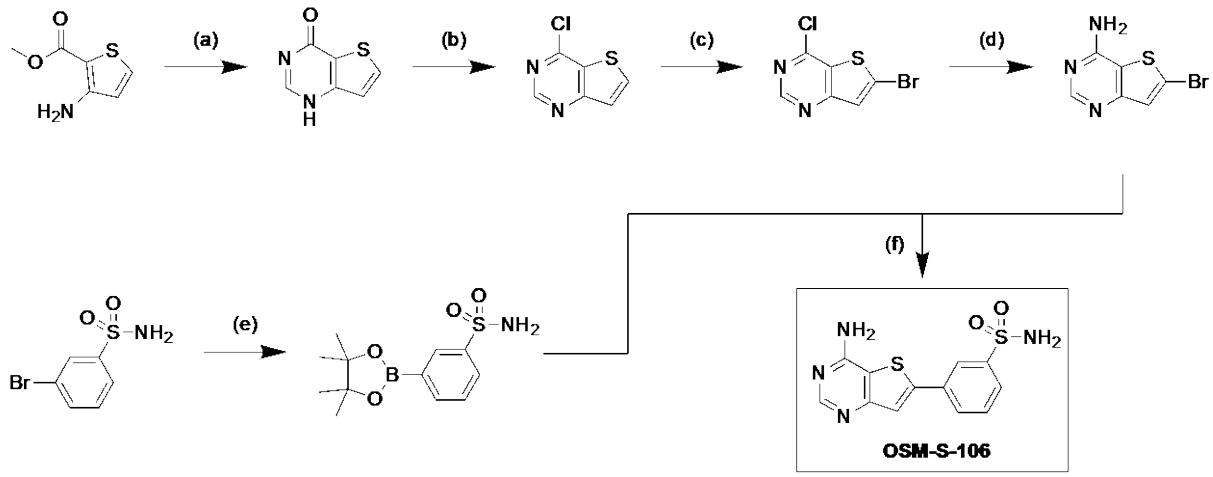

Scheme 1: Synthetic route to OSM-S-106. Reagents and conditions: (a) ammonium formate, formic acid, formamide, 140 °C, 23 h; (b) $POCl_3$, reflux, 2.5 h; (c) THF, –78 °C, 10 min *then* *n*-BuLi, –78 °C, 40 min *then* Br_2, –78 °C, rt, 1 h ; (d) 28% NH_4OH solution, isopropanol, 120 °C, 18 h; (e) bis(pinacolatodiboron), potassium acetate, dioxane, $PdCl_2$(dppf)·DCM, microwave, 140 °C, 60 min; (f) General procedure 1: $PdCl_2$(dppf)·DCM, 1 M aq. K_2CO_3, *i*-PrOH, microwave, 90 °C, 90 min

Thieno[3,2-*d*]pyrimidin-4(1*H*)-one (1)

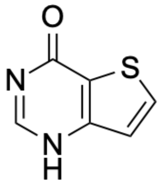

1

Methyl-3-aminothiophene-2-carboxylate (2.79 g, 17.8 mmol, 1 equiv.), ammonium formate (1.19 g, 18.8 mmol, 1 equiv.) and formic acid (0.73 mL, 19 mmol, 1.1 equiv.) were combined to form a slurry. Formamide (4.30 mL, 100 mmol, 5 equiv.) was added, and the reaction mixture was stirred at 140 °C for 23 h. The solution was allowed to cool to rt, and the solvent was removed under reduced pressure. The resulting solid was washed with H_2O and then filtered and dried *in vacuo* to give *the title compound* as a cream needle-like solid (1.78 g, 67%); R_F = 0.28 (100% EtOAc); M.P. 219 – 220 °C (lit. (Song, 2007): 222 – 223 °C); ^{1}H NMR (500 MHz, $CDCl_3$) δ 8.16 (s, 1 H), 7.88 (d, *J* = 5.4 Hz, 1 H), 7.42 (d, *J* = 5.4 Hz, 1 H), 7.26 (s, 1 H) ppm; ^{13}C NMR (101 MHz, $CDCl_3$) δ 157.8, 159.2, 146.5, 135.9, 135.2, 112.8 ppm. The NMR data matched those in the literature (Wang et al., 2017) (Woodring et al., 2015).

4-Chlorothieno [3,2-*d*]pyrimidine (2)

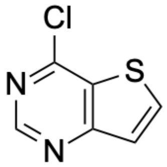

2

Phosphoryl chloride (13.7 mL, 147 mmol, 9.9 equiv.) was added to compound **1** (2.28 g, 15.0 mmol, 1 equiv.) under Ar. The reaction was heated at 110 °C until completion as indicated by TLC. The reaction mixture was allowed to cool to rt and neutralised to pH 7 with 4 M NaOH solution in an ice bath whilst stirring. The aqueous mixture was extracted with EtOAc (3 ×) and the combined organic layers were washed with brine, dried ($MgSO_4$), filtered and concentrated under reduced pressure to give the crude product which was purified by automated flash chromatography on silica (Biotage Isolera, 12 – 100% EtOAc in hexane) to give *the title compound* as a cream solid (0.87 g, 40%). R_F = 0.41 (20% EtOAc in hexane); M.P. 122.1 – 123.0 °C (lit. (Song, 2007) 123 – 124 °C); ^{1}H NMR (500 MHz, $CDCl_3$) δ 8.94 (s, 1 H), 8.00 (d, *J* = 5.5 Hz, 1 H), 7.55 (d, *J* = 5.5 Hz, 1 H); ^{13}C NMR (126 MHz, $CDCl_3$) δ 161.9, 155.0, 154.3, 137.0, 130.7, 125.1. The NMR data matched those in the literature (Ham et al., 2010) (Woodring et al., 2015).

6-Bromo-4-chlorothieno[3,2-*d*]pyrimidine (3)

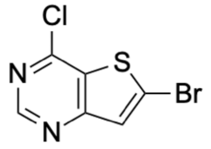

3

Compound **2** (4.55 g, 26.7 mmol, 1 equiv.) was dissolved in dry THF (100 mL) and stirred at –78 °C under an Ar atmosphere for 20 min. *n*-Butyllithium (1.09 M in hexane (Burchat et al., 1997), 26.0 mL, 28.4 mmol, 1.1 equiv.) was added to the solution at –78 °C. The stirring was continued at –78 °C for 40 min before bromine (1.50 mL, 29.3 mmol, 1.1 equiv.) was added. The reaction was stirred at rt for a further 1 h and quenched with sat. aq. $Na_2S_2O_3$ solution (100 mL). The solvent was removed under reduced pressure. Sat. aq. $Na_2S_2O_3$ solution (20 mL) and EtOAc (20 mL) were added to the residue, and the organic layer was separated. The aqueous phase was extracted with EtOAc (3 ×) and the combined organic layers were washed with H_2O (2 ×), brine (2 ×), dried ($MgSO_4$), filtered and concentrated under reduced pressure to give the crude product which was purified by automated flash chromatography on silica (Biotage Isolera, 0 – 15% EtOAc in hexane) to give *the title compound* as a light-yellow solid (4.07 g, 61%). R_F = 0.48 (10% EtOAc in hexane); M.P. 135.0 –135.5 °C (no lit. M.P.); ^{1}H NMR (500 MHz, $CDCl_3$) δ 8.94 (s, 1 H), 7.62 (s, 1 H) ppm; ^{13}C NMR (126 MHz, $CDCl_3$) δ 160.5, 153.8, 152.2, 131.5, 127.9, 126.8. The NMR data matched those in the literature (Woodring et al., 2015).

6-Bromothieno[3,2-*d*]pyrimidin-4-amine (4)

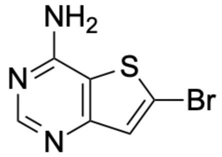

4

Compound **3** (35.2 mg, 140 mmol, 1.00 equiv.) was dissolved in isopropanol (4 mL). Ammonium hydroxide solution (28%, 900 mM, 0.550 mL, 140 mmol, 100 equiv.) was added and the reaction was heated in an oil bath set at 120 °C for 18 h. A 1:1 solution of EtOAc and H_2O (20 mL) was added, and the aqueous phase was extracted with EtOAc (3 ×). The combined organic layers were washed with brine (2 ×), dried ($MgSO_4$), filtered and concentrated under reduced pressure to give the crude product which was purified by automated flash chromatography on silica (Biotage Isolera, 15 – 100% EtOAc in hexane) to give *the title*

compound as a white solid (28.0 mg, 87%). R_F = 0.11 (50% EtOAc in hexane); M.P. 241 – 242 °C (no lit. M.P.); ^{1}H NMR (400 MHz, $CDCl_3$) δ 8.33 (s, 1 H), 7.55 (s, 1 H), 7.50 (s, 2 H, NH_2) ppm; ^{13}C NMR (101 MHz, $CDCl_3$) δ 159.6, 157.0, 155.4, 127.6, 122.4, 115.4; *m/z* (ESI+) 231 ($[M+H]^+$, 100%); HRMS (ESI+) found 231.9369 ($[M+H]^+$), $C_6H_4BrN_3S^+$ requires 231.9309.

3-(4,4,5,5-Tetramethyl-1,3,2-dioxaborolan-2-yl)benzenesulfonamide (5)

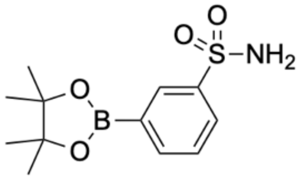

5

3-Bromobenzenesulfonamide (500 mg, 2.12 mmol, 1 equiv.), *bis*(pinacolatodiboron) (645 mg, 2.54 mmol, 1.2 equiv.), potassium acetate (626 mg, 6.37 mmol, 3 equiv.) and $PdCl_2$(dppf)·DCM (34.6 mg, 42.4 mmol, 0.02 equiv.) were combined in a microwave vial that was filled with Ar and evacuated three times. Dioxane (20 mL, 0.10 M) was added, and the reaction mixture was degassed before heating under microwave irradiation at 140 °C for 1 h. The reaction mixture was diluted with MeOH (10 mL) and filtered through celite. The filtrate was concentrated and the residue was purified by automated flash chromatography on silica (Biotage Isolera, 10 – 100% EtOAc in hexane) to give *the title compound* as a white solid (570 mg, 95%). R_F = 0.12 (20% EtOAc in hexane); M.P. 215 – 216 °C (no lit. M.P.); ^{1}H NMR (400 MHz, DMSO-d_6) δ 8.14 (s, 1 H), 7.91 – 7.94 (m, 1 H), 7.84 – 7.86 (m, 1 H), 7.57 – 7.61 (m, 1 H), 7.36 (s, 2 H, NH_2), 1.32 (s, 12 H); *m/z* (ESI+) 589 ($[2M+Na]^+$, 100%). The spectroscopic data matched those in the literature (Abdelsamie et al., 2017).

3-(4-Aminothieno[3,2-*d*]pyrimidin-6-yl)benzenesulfonamide (OSM-S-106)

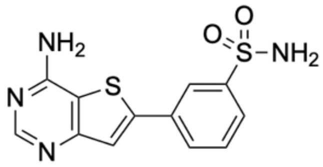

OSM-S-106

Prepared according to General Procedure 1 from: compound **4** (195 mg, 0.847 mmol, 1 equiv.), compound **5** (265 mg, 0.936 mmol, 1.1 equiv.) and $PdCl_2$(dppf)·DCM (69 mg, 0.085 mmol, 0.10 equiv.), *i*-PrOH (17 mL, 0.5 M) and 1 M aq. K_2CO_3 (2.50 mL, 0.25 mmol, 3 equiv.) under microwave irradiation at 90 °C for 1.5 h to give the crude title compound as a brown solid; purified by automated flash chromatography on silica (Biotage Isolera, 5 – 20% MeOH (with 10% MeOH): DCM) to give *the title compound* as a light brown solid (181 mg, 69%). R_F = 0.12 (10% MeOH (with 10% NH_4OH) in DCM); M.P. decomposes at 278 °C; ^{1}H NMR (400 MHz, DMSO-d_6) δ 8.39 (s, 1 H), 8.21 (s, 1 H), 8.11 (apparent dt, *J* = 8.1, 1.3 Hz, 1 H), 7.89 – 7.87 (m, 2 H), 7.72 (apparent t, *J* = 7.8 Hz, 1 H), 7.52 (s, 2 H, NH_2), 7.50 (s, 2 H, NH_2) ppm; ^{13}C NMR (101 MHz, DMSO-d_6) δ 160.4, 158.0, 155.4, 147.0, 145.2, 133.5, 130.3, 129.4, 126.3, 123.0, 121.7, 113.8; *m/z* (ESI+) 307 ($[M+H]^+$, 100%); HRMS (ESI+) found 307.0322 ($[M+H]^+$), $C_{12}H_{11}N_4O_2S_2^+$ requires 307.0318.

Synthesis of OSM-E-32

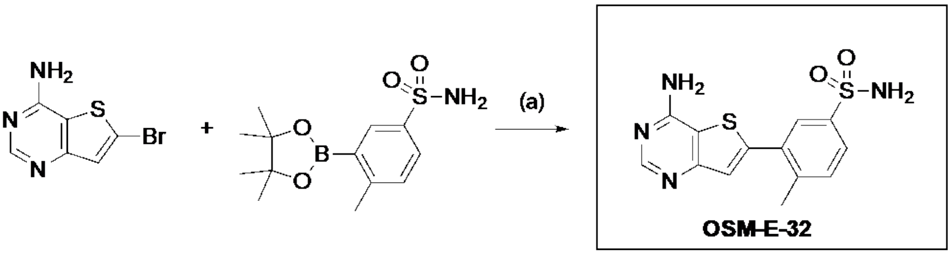

Scheme 2: Synthetic route to OSM-E-32. Reagents and conditions: (a) General procedure 1: $PdCl_2$(dppf)·DCM, 1 M aq. K_2CO_3, *i*-PrOH, microwave, 90 °C, 30 min.

3-(4-Aminothieno[3,2-*d*]pyrimidin-6-yl)-4-methylbenzenesulfonamide (OSM-E-32)

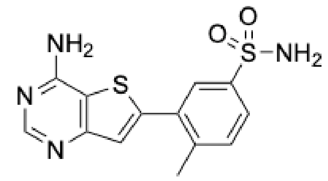

OSM-E-32

Prepared according to General Procedure 1 from: Compound **4** (28 mg, 0.10 mmol, 1 equiv.), 4-methyl-3 (4,4,5,5-tetramethyl-1,3,2-dioxaborolan-2-yl)benzenesulfonamide (47 mg, 0.12 mmol, 1.1 equiv.), and $PdCl_2$(dppf)·DCM (16 mg, 20 µmol, 20 mol%, 0.1 equiv.) were dissolved in *i*-PrOH (2 mL) and 1 M aq. K_2CO_3 (0.2 mL, 0.2 mmol, 2 equiv.) under microwave irradiation at 90 °C for 30 min to give the crude title compound; purified using column chromatography on silica (0 – 10% MeOH in DCM) to give *the title compound* as a tan solid (16.8 mg, 52%). M.P. decomposes at 293 °C; ^{1}H NMR (600 MHz, methanol-d_4) δ 8.41 – 8.42 (m, 1 H), 8.00 (s, 1 H), 7.87 – 7.89 (m, 1 H), 7.55 (d, *J* = 6.0 Hz, 1 H), 7.37 (s, 1H), 2.52 (s, 3 H) ppm (both amine and sulfonamide NH_2 signals not observed); ^{13}C NMR (151 MHz, methanol-d_4) δ 160.8, 160.3, 156.3, 150.4, 143.7, 142.8, 135.5, 133.2, 139.2, 126.3, 125.5, 116.9, 21.4; *m/z* (ESI+) 321 ($[M+H]^+$, 100%); HRMS (ESI+) found 321.0474 ($[M+H]^+$), $C_{13}H_{13}N_4O_2S_2^+$ requires 321.0480.

Synthesis of OSM-S-488

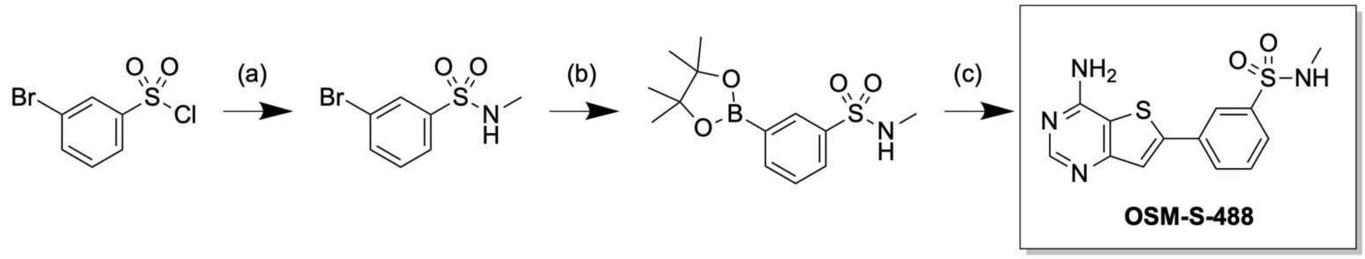

Scheme 3: Synthetic route to OSM-S-488. Reagents and conditions: (a) methylamine, Et_3N, THF, 0 °C then rt, 90 min; (b) bis(pinacolatodiboron), potassium acetate, dioxane, $PdCl_2$(dppf)·DCM, 100 °C, 18 h; (c) General procedure 1: $PdCl_2$(dppf)·DCM, 1 M aq. K_2CO_3, *i*-PrOH, microwave, 90 °C, 30 min.

3-Bromo-*N*-methylbenzenesulfonamide (9)

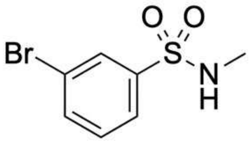

9

Methylamine (1.3 mL, 15 mmol, 5.0 equiv) and Et_3N (2.0 mL, 15 mmol, 5.0 equiv) were dissolved in 6 mL THF and the mixture was cooled to 0 °C. 3-Bromobenzenesulfonylchloride (750 mg, 2.90 mmol, 1.0 equiv) was added and the reaction was allowed to warm to rt for 90 min. The aqueous phase was extracted with EtOAc (3 ×). The combined organic layers were washed with brine (2 ×), dried ($MgSO_4$), filtered and concentrated under reduced pressure to give *the title compound* as a white solid (607 mg, 91%). ^{1}H NMR (500 MHz, $CDCl_3$) δ 8.04 (apparent t, *J* = 1.8, 1 H), 7.86 – 7.80 (m, 1 H), 7.75 (ddd, *J* = 8.0, 1.8, 1.0, 1 H), 7.44 (apparent t, *J* = 7.9, 1 H), 2.73 (d, *J* = 5.5, 3 H) ppm; ^{13}C NMR (126 MHz, $CDCl_3$) δ 140.87, 135.80, 130.66, 130.16, 125.76, 123.18, 29.39; *m/z* (ESI+) 363.3 ($[M+H]^+$, 100%). The spectroscopic data matched those in the literature (Liang et al., 2017).

N-Methyl-3-(4,4,5,5-tetramethyl-1,3,2-dioxaborolan-2-yl)benzenesulfonamide (10)

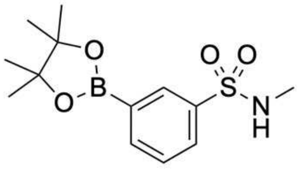

10

Compound **9** (93 mg, 0.37 mmol, 1 equiv.), *bis*(pinacolatodiboron) (284 mg, 1.12 mmol, 4.5 equiv.), potassium acetate (294 mg, 3.00 mmol, 12 equiv.) and $PdCl_2$(dppf)·DCM (30 mg, 0.070 mmol, 0.2 equiv.) were dissolved in 1,4-dioxane (4 mL) and heated at 100 °C in a sealed tube for 18 h. The mixture was concentrated under vacuum and the residue was purified by flash column chromatography (0–10% methanol in chloroform) to give *the title compound as* a brown crude solid (167.3 mg); which carried forward without further purification. R_F = 0.15 (50% EtOAc in hexane); *m/z* (ESI+) 321.1 ($[M+Na]^+$, 85%)

3-(4-Aminothieno[3,2-*d*]pyrimidin-6-yl)-*N*-methylbenzenesulfonamide (OSM-S-488)

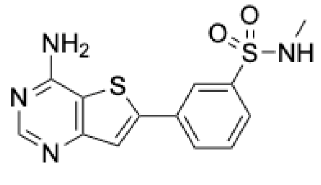

OSM-S-488

Prepared according to General Procedure 1 from: compound **4** (46 mg, 0.20 mmol, 1 equiv.), compound **10** (120 mg, 0.40 mmol, 2 equiv.), $PdCl_2$(dppf)·DCM (32 mg, 39 µmol, 20 mol%, 0.1 equiv.), *i*-PrOH (4 mL) and 1 M aq. K_2CO_3 (0.4 mL, 0.4 mmol) under microwave irradiation at 90 °C for 30 min to give the crude title compound; purified using column chromatography on silica (0 – 10% MeOH in DCM) to give *the title compound* as a pale brown solid (38.9 mg, 80%); **R_F** = 0.51 (10% MeOH in DCM); **M.P.** decomposes at 287.6 °C; **^{1}H NMR** (400 MHz, DMSO-d_6) δ 8.40 (s, 1H), 8.18 – 8.13 (m, 2H), 7.92 (s, 1H), 7.85 (apparent dt, *J* = 7.9, 1.3 Hz, 1H), 7.77 (apparent t, *J* = 7.8 Hz, 1H), 7.62 (br d, *J* = 4.7 Hz, 1H), 7.53 (s, 2H), 2.47 (d, *J* = 3.6 Hz, 5H) ppm; **^{13}C NMR** (101 MHz, DMSO-d_6) δ 160.4, 158.0, 155.4, 146.8, 140.4, 133.8, 130.5, 130.0, 127.3, 123.8, 121.9, 113.8, 28.7; ***m/z*** (ESI+) 321.1 ([M+H]$^+$, 100%); HRMS (ESI+) found 321.0478 ([M+H]$^+$), $C_{13}H_{20}NO_4S^+$ requires 321.0474.

Synthesis of OSM-LO-80

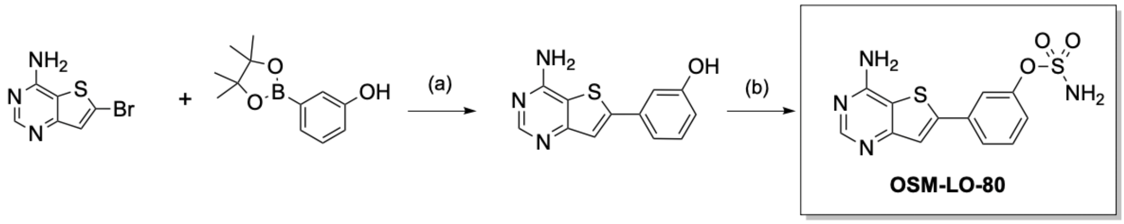

Scheme 4: Synthetic route to OSM-LO-80. Reagents and conditions: (a) $PdCl_2$(dppf)·DCM, potassium acetate, dioxane, microwave, 90 °C, 90 min; (b) sulfamoyl chloride, PhMe, DMA, 0 °C, overnight.

3-(4-Aminothieno[3,2-*d*]pyrimidin-6-yl)phenol (11)

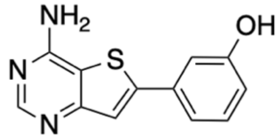

11

Prepared according to General Procedure 1 from: compound **4** (200 mg, 0.900 mmol, 1 equiv.), 3-(4,4,5,5-tetramethyl-1,3,2-dioxaborolan-2-yl)phenol (233 mg, 1.06 mmol, 1.22 equiv.), $PdCl_2$(dppf)·DCM (71 mg, 0.090 mmol, 0.1 equiv.), *i*-PrOH (17 mL, 0.5 M) and 1 M K_2CO_3 (2.61 mL, 2.61 mmol, 3 equiv.) under microwave irradiation at 90 °C for 90 min to give the crude title compound as a dark brown solid; purified by automated flash chromatography on silica (Biotage Isolera, 5 – 20% MeOH in DCM) to give *the title compound* as a brown solid (88 mg, 42%). R_F = 0.2 (10% MeOH in DCM); M.P. decomposes at 175.1 °C (no lit. M.P.); ^{1}H NMR (400 MHz, DMSO-d_6) δ 9.78 (s, 1 H), 8.36 (s, 1 H), 7.68 (s, 1 H), 7.42 (s, 2 H, NH_2), 7.33 – 7.36 (m, 2 H), 7.18 (s, 1 H), 6.87 – 6.84 (m, 1 H) ppm; ^{13}C NMR (101 MHz, DMSO-d_6) δ 160.5, 158.0, 157.9, 155.2, 149.0, 134.0, 130.4, 120.2, 117.0, 116.6, 113.1, 112.8; *m/z* (ESI+) 243.9 ([M+H]$^{+,}$ 100%). HRMS (ESI+) found 244.0566 ([M+H]$^+$), $C_{12}H_9N_3OS^+$ requires 244.0466.

3-(4-Aminothieno[3,2-*d*]pyrimidin-6-yl)phenyl sulfamate (OSM-LO-80)

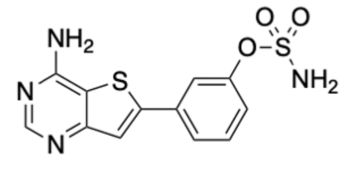

OSM-LO-80

A solution of sulfamoyl chloride (470 mg, 4.1 mmol, 10 equiv.) in PhMe (4 mL) was added dropwise to a solution of compound **6** (99 mg, 0.41 mmol, 1 equiv.) in DMA (4 mL) at 0 °C. The reaction mixture was stirred at rt overnight then quenched with H_2O (6 mL). The mixture was filtered and concentrated under reduced pressure to give a yellow crude oil. A few drops of DCM were added to form a white precipitate. The mixture was filtered and the solid was purified by reverse-phase automated flash chromatography on silica (Biotage Isolera, 5 – 50 % MeOH in H_2O) to give *the title compound* as a white solid (22 mg, 17 %). R_F = 0.55 (15% MeOH in DCM); M.P. decomposes at 269.7 °C (no lit. M.P.); ^{1}H NMR (400 MHz, DMSO-d_6) δ 8.39 (s, 1 H), 8.12 (s, 2 H), 7.87 – 7.78 (m, 2 H), 7.71 (apparent t, J = 2.0 Hz, 1 H), 7.61 (apparent t, J = 8.0 Hz, 1 H), 7.51 (s, 2 H), 7.36 (ddd, J = 8.1, 2.3, 0.9 Hz, 1 H); ^{13}C NMR (101 MHz, DMSO-d_6) δ 160.8, 158.4, 155.6, 151.1, 147.7, 134.8, 131.2, 124.7, 123.5, 121.7, 120.1, 113.9; *m/z* (ESI+) 323 ([M+H]$^{+,}$ 100%); HRMS (ESI+) found 323.0274 ([M+H]$^+$), $C_{12}H_{11}N_4O_3S_2^+$ requires 323.0267. IR ν_{max} (film) /cm^{-1} 1580, 1350, 1150.

Synthesis of OSM-LO-81

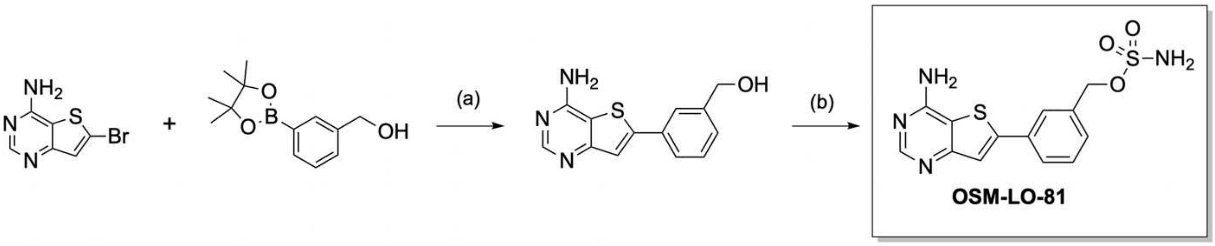

Scheme 5: Synthetic route to OSM-LO-81. Reagents and conditions: (a) $PdCl_2$(dppf)·DCM, potassium acetate, dioxane, 400 W microwave, 90 °C, 90 min; (b) Sulfamoyl chloride, DMF, rt, 5 h.

(3-(4-Aminothieno[3,2-*d*]pyrimidin-6-yl)phenyl)methanol (12)

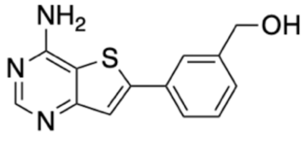

12

Prepared according to General Procedure 1 from: compound **4** (50.0 mg, 0.22 mmol, 1 equiv.), (3-(hydroxymethyl)phenyl)boronic acid (40 mg, 0.27 mmol, 1.2 equiv.), $PdCl_2$(dppf)·DCM (18 mg, 0.065 mmol, 0.1 equiv.), *i*-PrOH (6 mL, 0.5 M) and 1 M aq. K_2CO_3 (1.24 mL, 1.24 mmol) under microwave irradiation at 90 °C for 90 min to give the crude title compound as a dark brown solid; purified by automated flash chromatography on silica (Biotage Isolera, 5 – 20% MeOH in DCM) to give *the title compound* as a brown solid (33.7 mg, 60%). R_F = 0.3 (10% MeOH in DCM); M.P. decomposes at 201 °C (no lit. M.P.); ^{1}H NMR (400 MHz, DMSO-d_6) δ 8.37 (s, 1 H), 7.81 – 7.70 (m, 3 H), 7.52 – 7.24 (m, 4 H), 5.33 (apparent t, J = 5.8 Hz, 1 H), 4.59 (d, J = 5.7 Hz, 2 H); ^{13}C NMR (101 MHz, DMSO-d_6) δ 160.6, 157.9, 155.2, 149.0, 143.8, 132.6, 129.1, 127.5, 124.5, 124.0, 120.3, 113.2, 62.5; *m/z* (ESI+) 258 ([M+H]$^+$, 100 %); HRMS (ESI+) found 258.0707 ([M+H]$^+$), $C_{13}H_{12}N_3OS^+$ requires 258.0696.

3-(4-Aminothieno[3,2-*d*]pyrimidin-6-yl)benzyl sulfamate (OSM-LO-81)

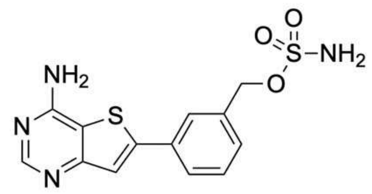

OSM-LO-81

Compound **7** (105 mg, 0.410 mmol, 1 equiv.) was dissolved in DMF (1.2 mL). Sulfamoyl chloride (143 mg, 1.24 mmol, 3.03 equiv) was added, and the mixture was stirred at rt for 5 h. The mixture was poured into a sat. aq. $Na_2S_2O_3$ solution and extracted with EtOAc (2 ×). The combined organic layers were washed with H_2O (2 ×), brine (2 ×), dried ($MgSO_4$), filtered and concentrated under reduced pressure to give the crude product which was purified by automated flash chromatography on silica (Biotage Isolera, 5 – 15% MeOH in DCM) to give *the title compound* as a white solid (41.3 mg, 30%). R_F = 0.4 (10% MeOH in DCM);

M.P. decomposes at 240 °C (no lit. M.P.); ^{1}H NMR (400 MHz, DMSO-d_6) δ 8.36 (s, 1 H), 7.81 – 7.87 (m, 3 H), 7.68 (s, 2 H), 7.46 – 7.56 (m, 4 H), 5.17 (s, 2 H); ^{13}C NMR (101 MHz, DMSO-d_6) δ 160.3, 157.8, 155.1, 148.0, 135.9, 132.9, 129.4, 129.0, 126.1, 125.7, 120.6, 113.2, 69.5; *m/z* (ESI+) 337 ([M+H]$^+$, 100%); HRMS (ESI+) found 337.0430 ([M+H]$^+$), $C_{13}H_{11}N_4O_3S_2^+$ requires 327.0424.

Synthesis of OSM-S-137

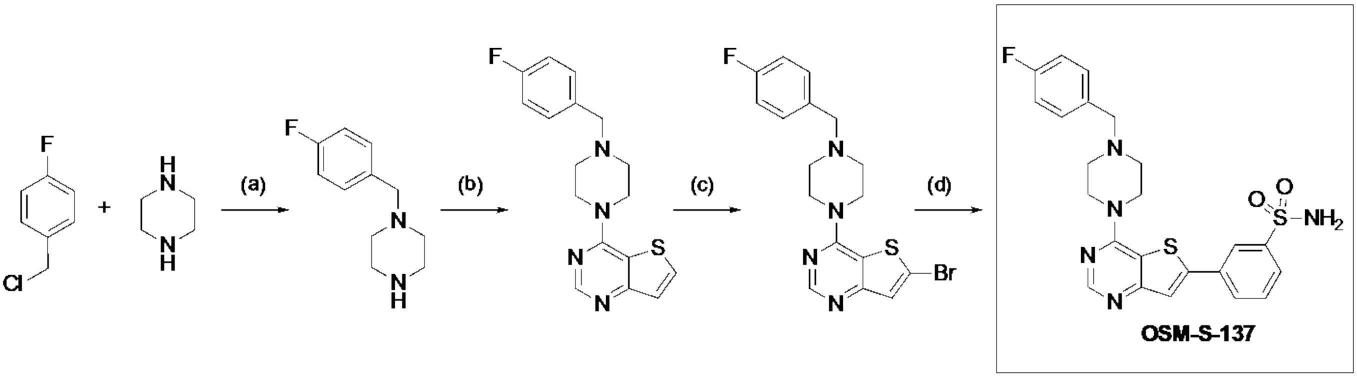

Scheme 6: Synthetic route to OSM-S-137. Reagents and conditions: (a) THF, reflux, 1 h; (b) compound 2, 80 °C under Ar atmosphere, 19 h; (c) THF, –78 °C, 10 min *then* *n*-BuLi, –78 °C, 40 min *then* Br_2, –78 °C, rt, 1 h; (d) compound **5**, $PdCl_2$(dppf)·DCM, potassium acetate, *i*-PrOH, 70 °C, 15 h under N_2.

1-(4-Fluorobenzyl)piperazine (13)

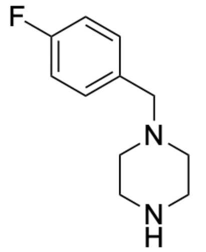

13

Anhydrous piperazine (4.98 g, 57.8 mmol, 6 equiv.) was added to THF (20 mL) and the mixture was heated at reflux until the piperazine was fully dissolved. 4-Fluorobenzyl chloride (1.2 mL, 10.0 mmol, 1 equiv.) was added dropwise and a white precipitate was formed immediately. The reaction mixture was heated at reflux for 1 h and then allowed to cool to rt. The mixture was filtered and washed with THF (100 mL) and then redissolved with EtOAc (100 mL). The combined organic layers were washed with 1 M KOH solution (2 ×), H_2O (2 ×), brine (2 ×), dried ($MgSO_4$), filtered and concentrated under reduced pressure to give a residue that was purified by automated flash chromatography on silica (Biotage Isolera, 0 – 100% MeOH in EtOAc) to give *the title compound* as a white solid (449 mg, 23%). R_F = 0.15 (50% MeOH in EtOAc); ^{1}H NMR (400 MHz, $CDCl_3$) δ 7.29 – 7.23 (m, 2 H), 7.02 – 6.95 (m, 2 H), 3.45 (s, 2 H), 3.18 (br s, 1 H), 2.94 – 2.89 (m, 4 H), 2.44 (s, 4 H); ^{13}C NMR (101 MHz, $CDCl_3$) δ 161.0 (d, *J* = 246.6 Hz), 133.8 (d, *J* = 4.3 Hz), 130.8, 115.2, 62.8, 53.8, 45.8; *m/z* (ESI+) 195.1 ([M+H]$^+$, 100%). The NMR data matched those in the literature (Zhang et al., 2011).

4-(4-(4-Fluorobenzyl)piperazin-1-yl)thieno[3,2-*d*]pyrimidine (14)

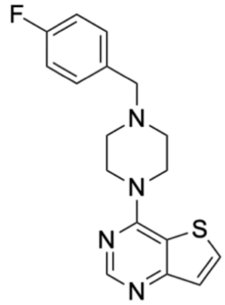

14

Compound **13** (1.97 g, 10.1 mmol, 2 equiv.) was dissolved in EtOH (24 mL) and compound **2** (870 mg, 5.09 mmol, 1 equiv.) was added. The reaction mixture was heated at 80 °C under Ar atmosphere for 19 h. The reaction mixture was cooled to rt and H_2O was added to give a white precipitate. Ethanol was removed under reduced pressure to give a precipitate that was filtered to give *the title compound* as a cream solid (1.4 g, 85%). R_F = 0.18 (70% EtOAc in hexane); M.P. 125 – 126 °C (no lit. M.P.); ^{1}H NMR (400 MHz, $CDCl_3$) δ 8.54 (s, 1 H), 7.70 – 7.68 (d, *J* = 5.6 Hz, 1 H), 7.41 – 7.40 (d, *J* = 5.6 Hz, 1 H), 7.32 – 7.28 (m, 2 H), 7.03 – 6.98 (m, 2 H), 4.02 – 3.97 (m, 4 H), 3.51 (s, 2 H), 2.59 – 2.53 (m, 4 H) ppm; ^{13}C NMR (101 MHz, $CDCl_3$) δ 162.4 (d, *J* = 246.6 Hz), 161.5, 158.1, 154.4, 133.48 (d, *J* = 4.1 Hz), 131.4, 130.66, 125.4, 115.4 (d, *J* = 22.7 Hz), 114.5, 62.2, 53.0, 46.1; *m/z* (ESI+) 329.4 ([M+H]$^+$, 100 %).

6-Bromo-4-(4-(4-fluorobenzyl)piperazin-1-yl)thieno[3,2-*d*]pyrimidine (15)

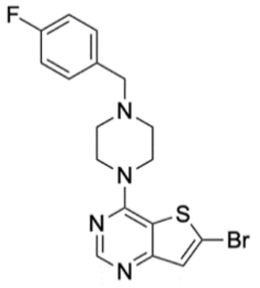

15

Compound **14** (1.04 g, 3.15 mmol, 1 equiv.) was dissolved in dry THF (60 mL) and stirred at – 78 °C under an Ar atmosphere for 20 min. *n*-Butyllithium (1.6 M in hexane hexane (Burchat et al., 1997), 3.1 mL, 5.0 mmol, 1.6 equiv.) was added to the solution at –78 °C. The stirring was continued at –78 °C for 40 min before bromine (0.34 mL, 6.6 mmol, 2.1 equiv.) was added. The reaction was stirred at rt for a further 2 h and quenched with sat. aq. $Na_2S_2O_3$ solution (100 mL). The solvent was removed under reduced pressure. Sat. aq. $Na_2S_2O_3$ solution (20 mL) and EtOAc (20 mL) were added to the residue, and the organic layer was separated. The aqueous phase was extracted with EtOAc (3 ×) and the combined organic layers were washed with H_2O (2 ×), brine (2 ×), dried ($MgSO_4$), filtered and concentrated under reduced pressure to give a brown crude product which was purified by automated flash chromatography on silica (Biotage Isolera, 50 – 100% EtOAc in hexane) to give *the title compound* as an orange solid (514 mg, 40%). R_F = 0.18 (50% EtOAc in hexane); M.P. 91 – 93 °C (no lit. M.P.); ^{1}H NMR (400 MHz, $CDCl_3$) δ 8.50 – 8.48 (m, 1 H), 7.40 (s, 1 H), 7.32 – 7.27 (m, 2 H), 7.03 (d, *J* = 8.6 Hz, 2 H), 3.93 (d, *J* = 4.8 Hz, 4 H), 3.53 – 3.51 (m, 2 H), 2.57 (d, *J* = 4.8 Hz, 4 H) ppm; ^{13}C NMR (101 MHz, $CDCl_3$) δ 162.4 (d, *J* = 246.6 Hz), 161.2, 156.8, 154.8, 133.3 (d, *J* = 4.1 Hz), 130.8, 128.1, 122.8, 116.2, 115.5 (d, *J* = 22.7 Hz), 62.2, 52.9, 46.1. *m/z* (ESI+) 407.0 ($[M+H]^+$, 100 %).

3-(4-(4-(4-Fluorobenzyl)piperazin-1-yl)thieno[3,2-*d*]pyrimidin-6-yl)benzenesulfonamide (OSM-S-137)

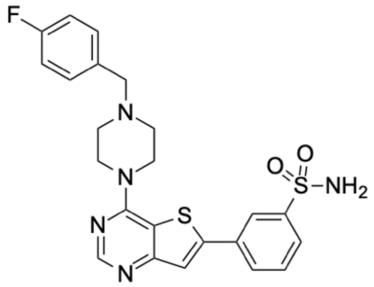

OSM-S-137

Prepared according to General Procedure 1 from: Compound **15** (51 mg, 0.13 mmol, 1 equiv.), compound **5** (38 mg, 0.13 mmol, 1 equiv.) and $PdCl_2$(dppf)·DCM (14 mg, 0.020 mmol, 0.15 equiv.), *i*-PrOH (3 mL) and K_2CO_3 solution (1 M, 0.2 mL, 0.2 mmol, 1.5 equiv.), heated at 70 °C for 15 h under Ar. The reaction was cooled and dissolved in ethyl acetate and water (1:1) (20 mL) and filtered through celite. The aqueous phase was extracted with EtOAc (3 ×) and the combined organic layers were washed with H_2O (2 ×), brine (2 ×), dried ($MgSO_4$), filtered and concentrated under reduced pressure to give *the title compound as* a brown crude product which was purified by automated flash chromatography on silica (Biotage Isolera, 10 – 100% EtOAc in hexane) and repurified by reversed-phase automated flash chromatography on silica (Biotage Isolera, 5 – 100 % MeOH in H_2O) to give *the title compound* as a brown solid (20 mg, 32%). R_F = 0.18 (100% EtOAc); M.P. decomposes at 226 °C (no lit. M.P.); ^{1}H NMR (500 MHz, DMSO-d_6) δ 8.51 (s, 1 H), 8.25 (s, 1 H), 8.16 (d, *J* = 8.1 Hz, 1 H), 7.97 (s, 1 H), 7.89 (d, *J* = 8.2 Hz, 1 H), 7.72 (apparent t, *J* = 7.8 Hz, 1 H), 7.50 (br s, 2 H), 7.38 (dd, *J* = 8.3, 5.6 Hz, 2 H), 7.17 (apparent t, *J* = 8.8 Hz, 2 H), 3.96 (s, 4 H), 3.54 (s, 2 H), 2.55 (s, 4 H) ppm; ^{13}C NMR (126 MHz, DMSO-d_6) δ 161.8 (d, *J* = 246.6 Hz), 161.3, 157.0, 154.5, 146.8, 145.2, 133.9 (d, *J* = 4.1 Hz), 132.9, 130.8, 129.5, 126.5, 123.0, 121.8, 115.0 (d, *J* = 22.7 Hz), 114.9, 113.4, 60.8, 52.3, 45.6; *m/z* (ESI+) 484.1 ($[M+H]^+$, 100 %); HRMS (ESI+) found 485.1306 ($[M+H]^+$), $C_{23}H_{23}FN_5O_2S_2^+$ requires 485.1306.

Synthesis of OSM-LO-87

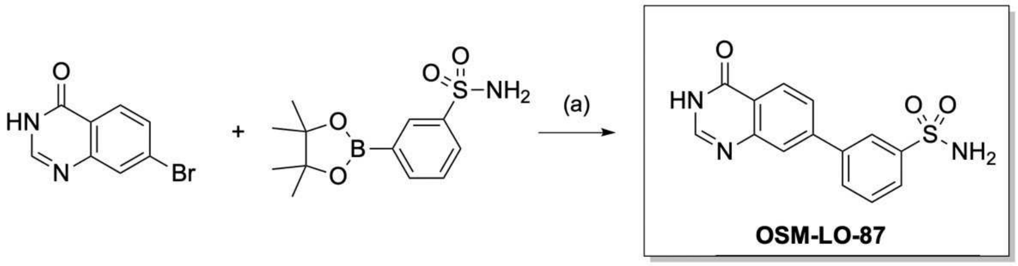

Scheme 7: Synthetic route to OSM-LO-87. Reagents and conditions: (a) General procedure 1: $PdCl_2$(dppf)·DCM, 1 M aq. K_2CO_3, *i*-PrOH, microwave, 90 °C, 90 min.

3-(4-Oxo-3,4-dihydroquinazolin-7-yl)benzenesulfonamide (OSM-LO-87)

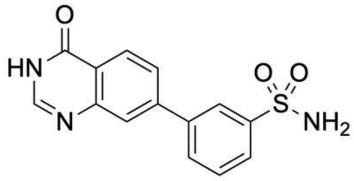

OSM-LO-87

Prepared according to General Procedure 1 from: 7-bromoquinazolin-4(3*H*)-one (60 mg, 0.030 mmol, 1 equiv.), 3-(4,4,5,5-tetramethyl-1,3,2-dioxaborolan-2-yl)benzenesulfonamide (90 mg, 0.030 mmol, 1.22 equiv.) and $PdCl_2$(dppf)·DCM (20 mg, 0.0030 mmol, 0.10 equiv.), *i*-PrOH (2 mL, 0.5 M) and 1 M aq. K_2CO_3 (0.8 mL, 0.8 mmol, 3 equiv.) under microwave irradiation at 90 °C for 1.5 h to give the crude title compound as a brown solid; purified by automated flash chromatography on silica (Biotage Isolera, 5 – 20% MeOH (with 10% MeOH): DCM) to give *the title compound* as a dark brown solid (30 mg, 43%). R_F = 0.25 (10% MeOH in DCM) ; M.P. decomposes at 269 °C; ^{1}H NMR (400 MHz, DMSO-d$_6$) δ 12.31 (s, 1 H), 8.29 – 8.21 (m, 2 H), 8.17 (s, 1 H), 8.06 (m, 1 H), 7.99 (d, *J* = 1.8 Hz, 1 H), 7.88 (td, J = 7.7, 1.7 Hz, 2 H), 7.73 (t, *J* = 7.8 Hz, 1H), 7.4 (s, 2 H) ppm; ^{13}C NMR (101 MHz, DMSO-d$_6$) δ 160.8, 149.4, 146.5, 145.0, 144.2, 139.4, 130.4, 129.9, 126.9, 125.4, 125.1, 124.9, 124.3, 122.1; *m/z* (ESI+) 302 ([M+H]$^+$, 100%); HRMS (ESI+) found 302.0600 ([M+H]$^+$), $C_{14}H_{11}N_3O_3S^+$ requires 302.0594.

Synthesis of OSM-LO-88

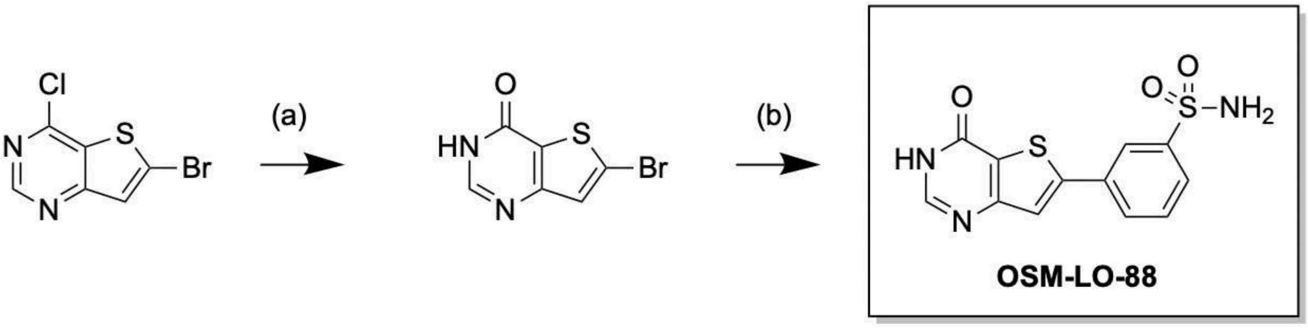

Scheme 8: Synthetic route to OSM-LO-88. Reagents and conditions: (a) 1 M NaOH, THF, 50 °C, overnight; (b) General procedure 1: $PdCl_2$(dppf)·DCM, 1 M aq. K_2CO_3, *i*-PrOH, microwave, 90 °C, 90 min.

6-Bromothieno[3,2-*d*]pyrimidin-4(3*H*)-one (16)

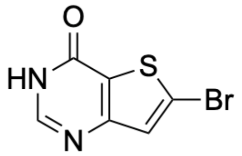

16

To a solution of 6-bromo-4-chlorothieno[3,2-d]pyrimidine (250 mg, 1.0 mmol, 1 equiv.) in THF (5 mL) was added 1 M NaOH (aq) (1.5 mL, 1.5 mmol, 1.5 equiv.). The reaction was heated at 50 °C overnight. The reaction was allowed to cool to rt and 1 M HCl (aq) was added until a neutral pH was achieved. The mixture was concentrated under reduced pressure to give the crude product which was purified by automated flash chromatography on silica (Biotage Isolera, 0 – 15% EtOAc in hexane) to give *the title compound* as a yellow solid (471 mg, 84%). R_F = 0.32 (50%

EtOAc in hexane); **^{1}H NMR** (400 MHz, DMSO-d$_6$) δ 12.64 (s, 1 H), 8.15 (s, 1 H), 7.62 (s, 1 H) ppm; *m/z* (ESI+) 230.0 ([M+H]$^+$, 100%); The NMR data matched those in the literature (De Schutter et al., 2014).

3-(4-Oxo-3,4-dihydrothieno[3,2-*d*]pyrimidin-6-yl)benzenesulfonamide (OSM-LO-88)

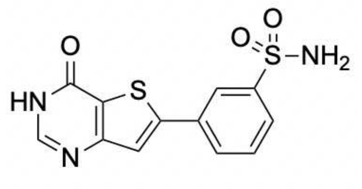

OSM-LO-88

Prepared according to General Procedure 1 from: compound **16** (45 mg, 0.20 mmol, 1 equiv.), 3-(4,4,5,5-tetramethyl-1,3,2-dioxaborolan-2-yl)benzenesulfonamide (67 mg, 0.24 mmol, 1.2 equiv.) and PdCl$_2$(dppf)·DCM (16 mg, 0.020 mmol, 0.10 equiv.), *i*-PrOH (6 mL, 0.5 M) and 1 M aq. K_2CO_3 (0.5 mL, 0.5 mmol, 3 equiv.) under microwave irradiation at 90 °C for 1.5 h to give the crude title compound as a brown solid which purified by automated flash chromatography on silica (Biotage Isolera, 5 – 20% MeOH (with 10% MeOH): DCM) to give *the title compound* as a dark brown solid (24.3 mg, 41%). R_F = 0.23 (5% MeOH in DCM); **^{1}H NMR** (400 MHz, DMSO-d$_6$) δ 8.23 (apparent t, *J* = 1.9 Hz, 1H), 8.20 (s, 1H), 8.12 (apparent dt, *J* = 8.0, 1.2 Hz, 1H), 7.93 (s, 1H), 7.90 – 7.87 (m, 1H), 7.71 (apparent t, *J* = 7.8 Hz, 1H), 7.50 (s, 2H); **^{13}C NMR** (101 MHz, DMSO-d$_6$) δ 158.4 156.9, 148.8, 147.3, 145.3, 133.2, 130.2, 129.4, 126.3, 123.0, 122.4; *m/z* (ESI+) 308 ([M+H]$^+$, 100%); **HRMS** (ESI+) found 308.0168 ([M+H]$^+$), $C_{12}H_9N_3O_3S_2^+$ requires 308.0158.

Synthesis of OSM-S-106-Asn adduct (UCL)

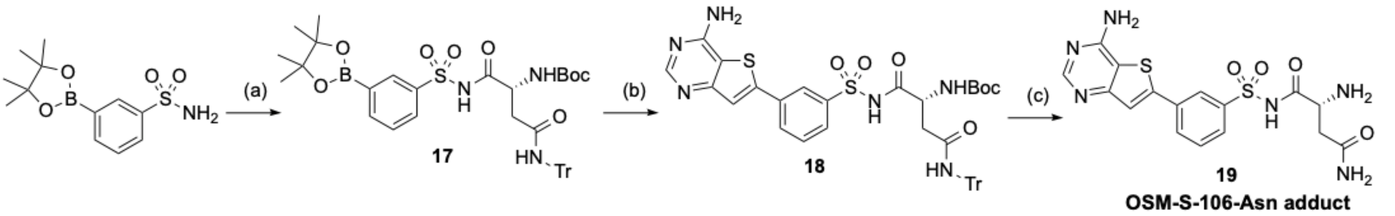

Scheme 9: Synthetic route of OSM-S-106-Asn adduct synthesis in UCL. Reagents and conditions: (a) Nα-Boc-Nγ-trityl-L-asparagine, DMAP, DCM, EDCl, 50 °C, 3 d; (b) 6-bromothieno[3,2-*d*] pyrimidin-4-amine, PdCl$_2$(dppf)•DCM, KOAc, dioxane, 400 W microwave, 90 °C, 90 min; (c) 4 M HCl, dioxane, 50 °C, overnight.

tert-Butyl (*R*)-(1,4-dioxo-1-((3-(4,4,5,5-tetramethyl-1,3,2-dioxaborolan-2-yl)phenyl) sulfonamido)-4-(tritylamino)butan-2-yl)carbamate (17)

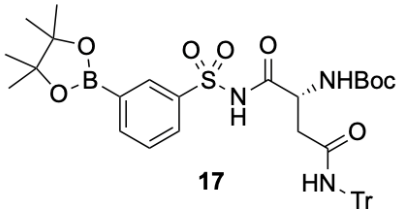

Compound **5** (250 mg, 0.883 mmol, 1 equiv.), Nα-Boc-Nγ-trityl-L-asparagine (625 mg, 1.32 mmol, 1.49 equiv.) and DMAP (479 mg, 3.92 mmol, 4.44 equiv.) were dissolved in DCM (9.0 mL, 0.1 M). EDCI (0.50 mL, 2.82 mmol, 3.2 equiv.) was added and the reaction was stirred at 50 °C for 3 d. The reaction mixture was diluted with DCM and poured into 1 M HCl (10 mL). The aqueous phase was extracted with DCM (2 ×) and the combined organic layers were washed with brine, dried (MgSO$_4$), and concentrated to give the crude material as a yellow residue (846 mg) that was used in the next reaction without further purification. *m/z* (ESI+) 762.3 ([M+Na]$^+$, 100%).

tert-Butyl (*R*)-(1-((3-(4-aminothieno[3,2-d] pyrimidin-6-yl) phenyl)sulfonamido)-1,4-dioxo-4-(tritylamino)butan-2-yl)carbamate (18)

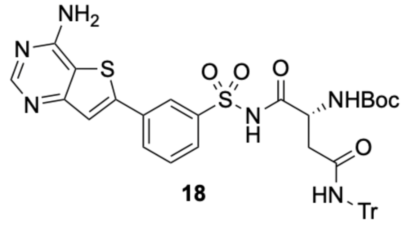

18

Prepared according to General Procedure 1 from: Compound **4** (180 mg, 0.782 mmol, 1 equiv.), crude **11** (653 mg, *ca.* 0.883 mmol, *ca.* 1.13 equiv.), $PdCl_2$(dppf)•DCM (64 mg, 0.078 mmol, 0.1 equiv.), *i*-PrOH (16.0 mL, 0.05 M) and 1 M aq. K_2CO_3 (2.4 mL, 2.40 mmol) under microwave irradiation at 90 °C for 90 min; purified by flash chromatography (5–10% MeOH (10% NH_4OH) in DCM), then repurified by reverse phase chromatography (5–100% MeOH in H_2O) to give *the title compound* as a light brown solid (207 mg, 35%); used in the next reaction without further purification. ^{1}H NMR (500 MHz, methanol-d_4) δ 8.37 (s, 1 H), 8.36 – 8.32 (m, 1 H), 8.01 – 7.86 (m, 2 H), 7.67 (s, 1 H), 7.60 – 7.51 (m, 1 H), 7.11 – 7.31 (m, 19 H), 4.31 – 4.41 (m, 1 H), 2.80 (s, 2 H), 2.40 – 2.54 (m, 1 H), 1.37 (s, 9 H) ppm.

(*R*)-2-amino-*N*^{1}-((3-(4-aminothieno[3,2-*d*]pyrimidin-6-yl)phenyl)sulfonyl)succinamide (19)

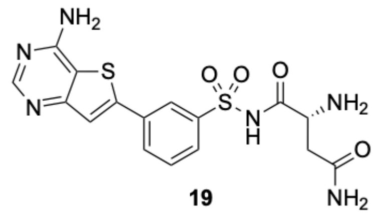

19

OSM-S-106-Asn adduct

Compound **12** (200 mg, 0.262 mmol, 1 equiv.) was dissolved in dioxane (2.2 mL, 0.12 M) and cooled to 0 °C. 4 M HCl in dioxane (1.3 mL, 5.2 mmol, 20 equiv.) was added dropwise and the reaction mixture was allowed to stir at 50 °C overnight. The volatiles were removed, and the crude material was purified by reverse phase chromatography (5 – 100% MeOH in H_2O). This material was then repurified by flash chromatography (5–40% 10% NH_4OH/ MeOH in DCM to give *the title compound* a white solid (11 mg, 10 %). ^{1}H NMR (400 MHz, DMSO-d_6) δ 8.38 (s, 1 H), 8.18 (s, 1 H), 7.96 (d, *J* = 7.6 Hz, 1 H), 7.84 (d, *J* = 7.6 Hz, 1 H), 7.80 (s, 1 H), 7.71 (s, 3 H), 7.60 – 7.51 (m, 2 H), 7.48 (s, 2 H), 7.10 (s, 1 H), 3.67 (dd, *J* = 9.3, 3.0 Hz, 1 H), 2.79 (dd, *J* = 17.1, 3.2 Hz, 1 H), 2.39 (dd, *J* = 16.9, 9.3 Hz, 1 H) ppm; ^{13}C NMR (500 MHz, DMSO-d_6) δ 171.7, 160.5, 158.0, 155.3, 148.0, 146.4, 132.2, 128.9, 127.9, 127.8, 124.6, 120.9, 113.4, 51.6, 35.3 ppm. *m/z* (ESI) 420.8 ($[M+H]^+$, 100%); HRMS (ESI+) found 421.0786 ($[M+H]^+$), $C_{16}H_{17}N_6O_4S_2$ requires 421.0747.

Alternative synthesis of OSM-S-106-Asn adduct (TCGLS)

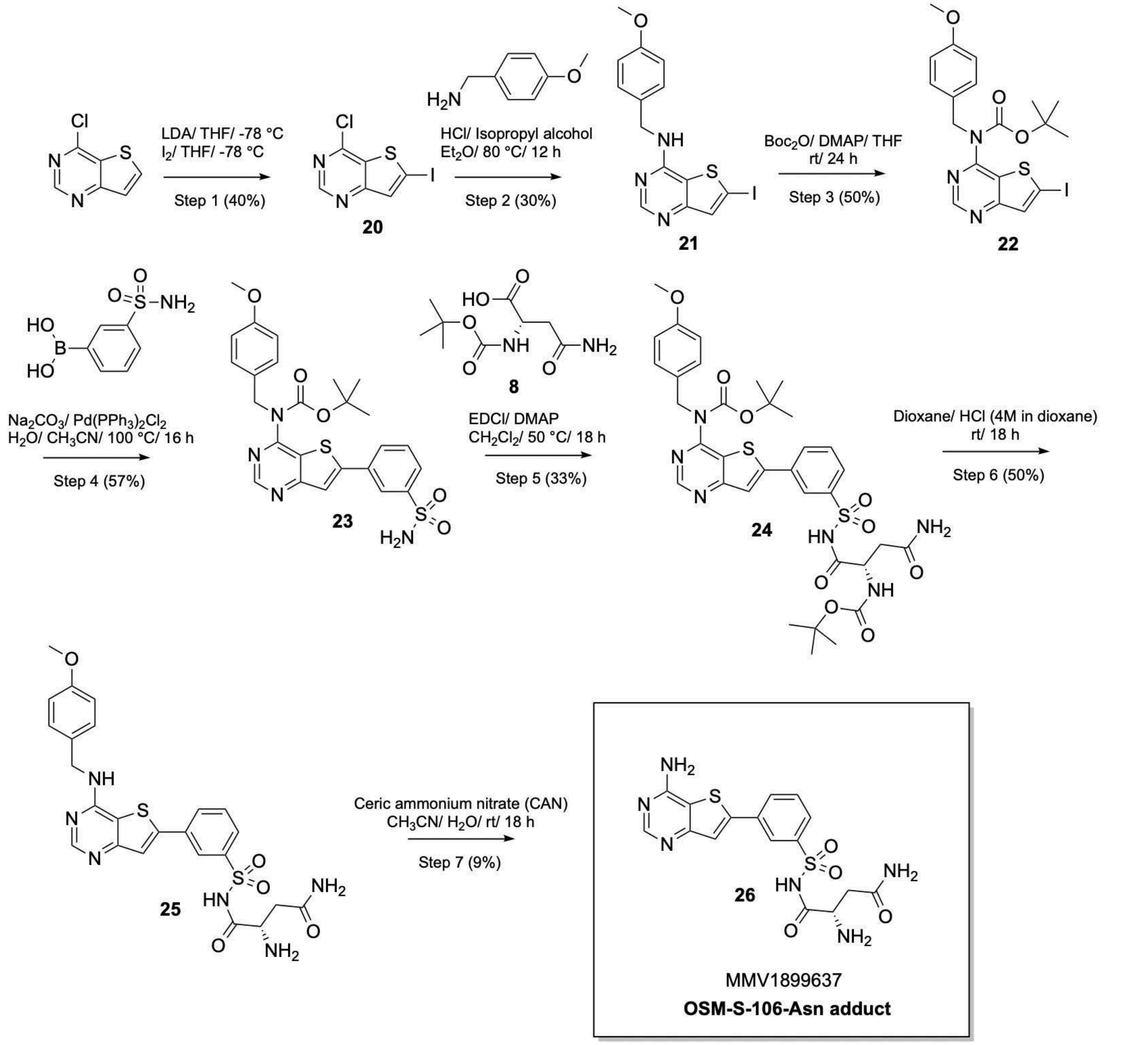

Scheme 10: Synthetic route of OSM-S-106-Asn adduct synthesis in TCGLS. Reagents and conditions are shown above**.** Compound 26 (also named as MMV1899637/OSM-S-106-Asn adduct) is identical in structure to compound 19 and shares the same data.

4-Chloro-6-iodothieno[3,2-*d*]pyrimidine (20):

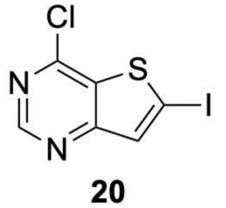

20

To a cooled solution of LDA (2 M in hexane, 10.7 mL, 21.4 mmol) in THF (30 mL) at -78 °C was added solution of 4-chlorothieno[3,2-*d*]pyrimidine (3 g, 17.6 mmol) in THF (20 mL). After stirring at -78 °C for 20 mins, solution of Iodine (5.42 g, 21.4 mmol) in THF (20 mL) was added. The reaction mixture was warmed to room temperature and stirred at room temperature for 30 mins. The reaction was quenched by adding 200 mL of chloroform. The reaction mixture was partitioned between chloroform and H_2O. The organic layer was collected, and the aqueous layer was extracted with 100 ml of chloroform. The combined organic layers were washed with of aqueous sodium thiosulfate twice and brine. The organic layer was dried over Na_2SO_4 and concentrated and purified by flash chromatography (20% EtOAc in hexane) to

give *the title compound* a white solid (2.1 g, 40% yield). ^1H NMR (400 MHz, DMSO) δ 8.96 (s, 1 H), 8.13 (s, 1 H) ppm; *m/z* (ESI) 297.06 ($[M+H]^+$, 100%). The NMR data matched those in the literature (Ni et al., 2011).

6-Iodo-N-(4-methoxybenzyl)thieno[3,2-*d*]pyrimidin-4-amine (21):

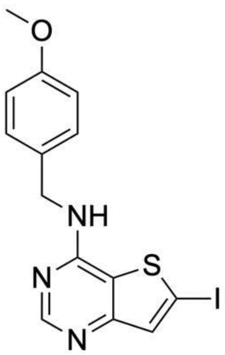

21

To the stirred solution of compound **20** (1.0 g, 3.4 mmol) in 2-propanol (12 mL) in a sealed tube was added compound (4-methoxyphenyl)methanamine (926 mg, 6.75 mmol) followed by HCl (2 mL, 2 M in Et_2O). The reaction mixture was heated at 80°C for 12 h. The reaction mixture was diluted with EtOAc and washed with saturated $NaHCO_3$ solution, organic extracts concentrated in vacuo to obtain a crude residue, which was purified by flash column chromatography (elution with 30 – 40% EtOAc in hexane) to *give the title compound* a white solid (400 mg, 30% yield). *m/z* (ESI) 398.2 ($[M+H]^+$, 100%). ^1H NMR (400 MHz, $CDCl_3$) δ 8.54 (s, 1 H), 7.6 (s, 1 H), 7.29 (d, *J* = 8.4 Hz, 2 H), 6.88 (d, *J* = 8.5 Hz, 2 H), 4.97 (s, 1 H), 4.75 (s, 2 H), 3.80 (s, 3 H). The NMR data matched those in the literature (Cai, 2007).

tert-Butyl (6-iodothieno[3,2-*d*]pyrimidin-4-yl)(4-methoxybenzyl)carbamate (22):

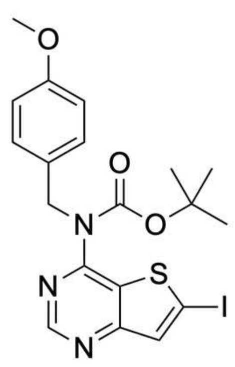

22

To the stirred solution of compound **21** (1.87 g, 4.72 mmol) in dry THF (20 mL), DMAP (633 mg, 5.18 mmol) was added at room temperature, the reaction mixture was stirred for 5 min at this temperature. To the reaction mixture was added Boc_2O (1.62 g, 7.07 mmol) and the reaction mixture was allowed to stir at rt for 24 h. The reaction mixture was partitioned between EtOAc and H_2O, organic extracts concentrated in vacuo to obtain a crude residue, which was purified by silica column chromatography (elution with 5 – 10% EtOAc in hexane) to *give the title compound* as a white solid (1.16 g, 50% yield). *m/z* (ESI) 497.5 ($[M+H]^+$, 100%); ^1H NMR (400 MHz, DMSO) δ 8.92 (s, 1 H), 7.90 (s, 1 H), 7.20 (d, *J* = 8.4 Hz, 2 H), 6.85 (d, *J* = 8.4 Hz, 2 H), 5.08 (s, 2 H), 3.69 (s, 3 H), 1.40 (s, 9 H).

tert-Butyl (4-methoxybenzyl)(6-(3-sulfamoylphenyl)thieno[3,2-*d*]pyrimidin-4-yl)carbamate (23):

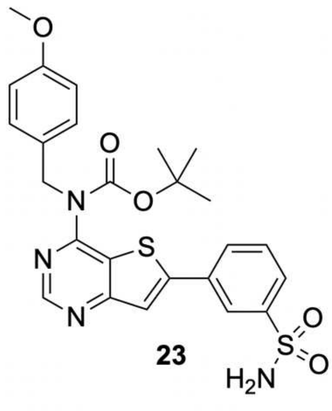

23

To a solution of compound 22 (750 mg, 1.5 mmol) and compound (3-sulfamoylphenyl)boronic acid (333 mg, 1.66 mmol) in MeCN (5 mL) in a sealed tube was added aqueous solution of Na2CO3 (2 M, 2.62 mL, 4.52 mmol) and the reaction mixture was purged with Ar for 20 mins. To the reaction mixture was added bis(triphenylphosphine)palladium(II) dichloride (105 mg, 0.151 mmol) and again purged with Ar for 5 mins. The reaction mixture was heated at 100 °C for 16 h. The reaction mixture was cooled to rt and filtered through the bed of celite, washed and extracted with EtOAc (2 x 20 mL). The combined organic layer was washed with brine, dried over over Na_2SO_4 and concentrated under reduced pressure to get crude which was purified by flash chromatography (50% EtOAc in hexane) to *give the title compound* a light brown solid (450 mg, 57% yield). *m/z* (ESI) 527 ($[M+H]^+$, 100%).

tert-Butyl (6-(3-(*N*-((*tert*-butoxycarbonyl)-*L*-asparaginyl)sulfamoyl)phenyl)thieno[3,2-*d*]pyrimidin-4-yl)(4-methoxybenzyl)carbamate (24):

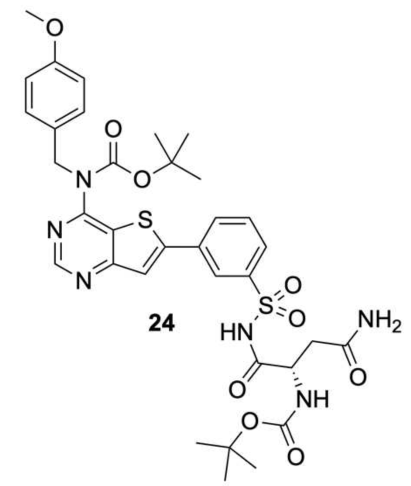

24

To a solution of compound (tert-butoxycarbonyl)-L-asparagine (595 mg, 2.56 mmol) in DCM (10 mL) was added EDCI (492 mg, 2.57 mmol), DMAP (627 mg, 5.13 mmol) and compound 23 (450 mg, 0.86 mmol) (10 mL) at rt and the reaction mixture was stirred at 50 °C for 18 h. The reaction mixture was concentrated under reduced pressure and crude compound was diluted with EtOAc and washed with H_2O and brine solution, dried over Na_2SO_4 and concentrated under reduced pressure to get crude which was purified by column chromatography (10% MeOH in DCM) to *give the title compound* as a light brown gummy solid (210 mg, 33% yield). *m/z* (ESI) 741.5 ($[M+H]^+$, 100%);

tert-Butyl (6-(3-(*N*-(*L*-asparaginyl)sulfamoyl)phenyl)thieno[3,2-*d*]pyrimidin-4-yl)(4-methoxybenzyl)carbamate (25):

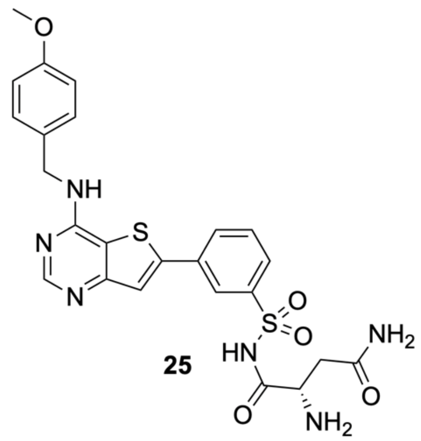

25

To a cooled solution of compound 24 (900 mg, 1.2 mmol) in dioxane (10 mL) at 0 °C was added 4 M HCl in dioxane (6 mL) dropwise. The reaction mixture was allowed to stir at rt for 18 h. The volatiles were removed, and the resultant residue was neutralized with NH_4OH, extracted with 10% MeOH in DCM. The combined organic layer was concentrated to *give the title compound* as a crude solid (330 mg, crude compound, 69% by LCMS) which was used of the next step as such. *m/z* (ESI) 541.5 ($[M+H]^+$, 100%);

(*S*)-2-amino-N^1-((3-(4-aminothieno[3,2-*d*]pyrimidin-6-yl)phenyl)sulfonyl)succinamide (26):

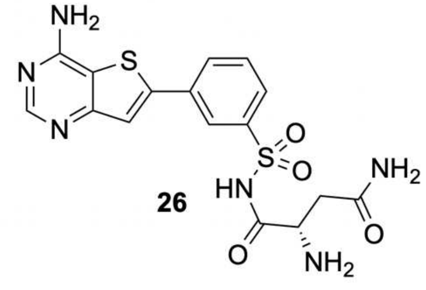

26

MMV1899637

OSM-S-106-Asn adduct

Compound 26 (also named as MMV1899637/OSM-S-106-Asn adduct) is identical in structure to compound 19 and shares the same data.

Synthesis of Asn-AMS (via General information 2)

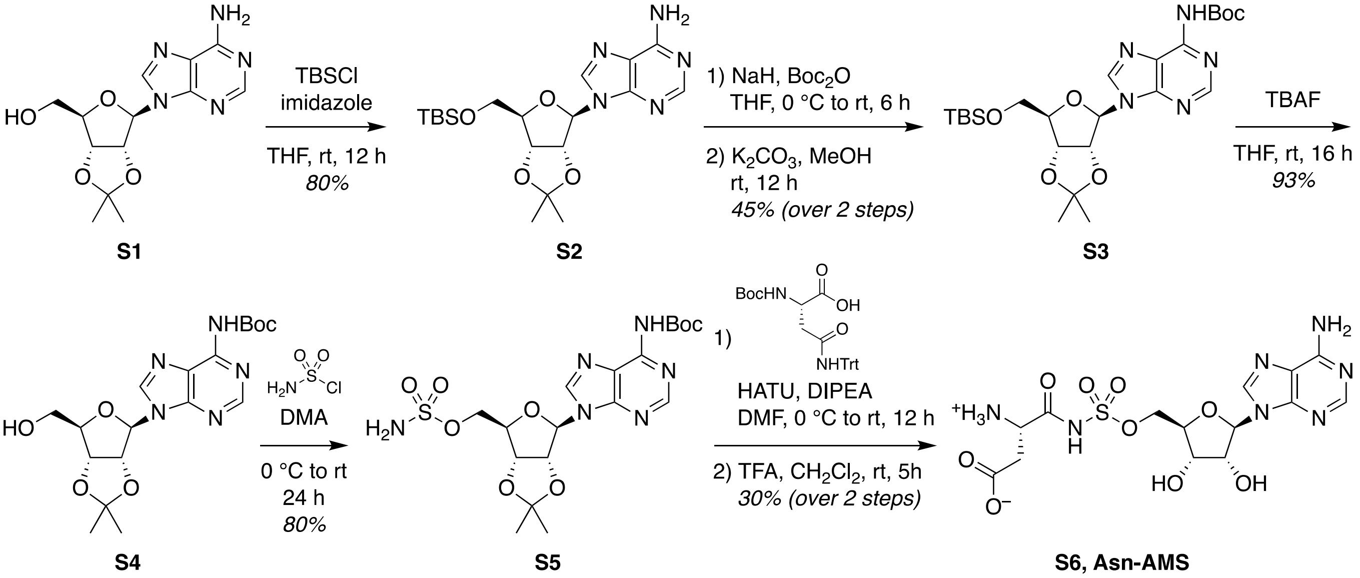

Scheme 11. Synthetic route of Asn-AMS. Reagents and conditions are shown above.

9-((3*aR*,4*R*,6*R*,6*aR*)-6-(((*tert*-Butyldimethylsilyl)oxy)methyl)-2,2-dimethyltetrahydrofuro[3,4-*d*][1,3]dioxol-4-yl)-9*H*-purin-6-amine (S2)

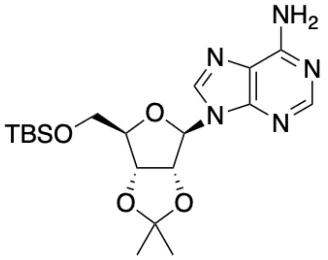

In a 250-mL round bottom flask, 2´,3´-*O*-isopropylideneadenosine **S1** (1 g, 3.25 mmol, 1.0 equiv) was dissolved in THF (20 mL) at rt, followed by addition of TBSCl (0.74 g, 4.88 mmol, 1.5 equiv) and imidazole (0.44 g, 6.5 mmol, 2.0 equiv). After 5 h, MeOH (2 mL) was added to quench excess TBSCl and the mixture was concentrated by rotary evaporation. The residue was diluted with EtOAc, washed with sat. aq. $NaHCO_3$ and brine, dried (Na_2SO_4), filtered, and concentrated by rotary evaporation. Purification by silica flash chromatography (50% EtOAc in hexane) to give *the title compound* as a white solid (1.1 g, 80%). Analytical data agreed with those reported previously (Ishikawa and Kakeya, 2014).

tert-Butyl (9-((3*aR*,4*R*,6*R*,6*aR*)-6-(((*tert*-butyldimethylsilyl)oxy)methyl)-2,2-dimethyltetrahydrofuro[3,4-*d*][1,3]dioxol-4-yl)-9*H*-purin-6-yl)carbamate (S3)

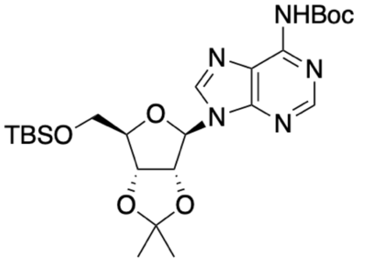

In a 250-mL round bottom flask, 5´-*O*-TBS adenosine **S2** (1 g, 2.37 mmol) was dissolved in THF (15 mL) and cooled to 0 °C, followed by addition of NaH (60% suspension in mineral oil, 164 mg, 3.55 mmol, 1.50 equiv). Once evolution of gas (H_2) had ceased, a solution of Boc_2O (815 µL, 3.55 mmol, 1.50 equiv) in THF (15 mL) was added with vigorous stirring at 0 °C. The reaction mixture was warmed to rt and stirred for 12 h. The reaction mixture was diluted with EtOAc and washed with sat. aq. $NaHCO_3$, then the aq layer was back-extracted with EtOAc. The combined organic extracts were washed with brine, dried (Na_2SO_4), and concentrated by rotary evaporation to afford a mixture of mono- and bis-Boc protected adenosines. The crude product was dissolved in MeOH (30 mL) at rt and K_2CO_3 (624 mg, 4.52 mmol, 2.00 equiv) was added. After 12 h, the reaction mixture was concentrated by rotary evaporation. The residue was extracted with EtOAc, washed with brine, dried (Na_2SO_4), and concentrated by rotary evaporation. Purification by silica flash chromatography (33% EtOAc in hexane) to *give the title*

compound as a white powder (480 mg, 40% over two steps). Analytical data agreed with those reported previously (Ishikawa and Kakeya, 2014).

tert-Butyl (9-((3*aR*,4*R*,6*R*,6*aR*)-6-(((*tert*-butyldimethylsilyl)oxy)methyl)-2,2-dimethyltetrahydrofuro[3,4-*d*][1,3]dioxol-4-yl)-9*H*-purin-6-yl)carbamate (S4)

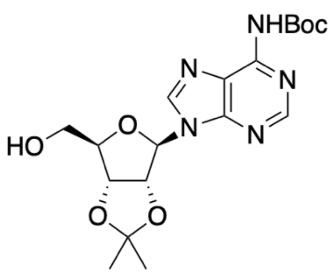

In a 250-mL round bottomed flask 5´-*O*-TBS adenosine **S3** (400 mg, 0.76 mmol) was dissolved in THF (10 mL) at rt, and TBAF (1 M in THF, 1.5 mL, 1.53 mmol, 2 equiv) was added. After 12 h, the reaction mixture was extracted with EtOAc, washed with brine, dried (Na_2SO_4), and concentrated by rotary evaporation. Purification by silica flash chromatography (75% EtOAc in hexane) to *give the title compound* as a white powder (287 mg, 93%). Analytical data agreed with those reported previously (Ishikawa and Kakeya, 2014).

tert-Butyl (9-((3*aR*,4*R*,6*R*,6*aR*)-2,2-dimethyl-6-((sulfamoylamino)methyl)tetrahydrofuro[3,4-*d*][1,3]dioxol-4-yl)-9*H*-purin-6-yl)carbamate (S5)

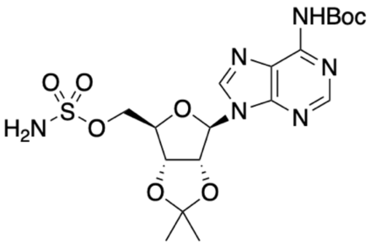

In a 50-mL round bottomed flask, protected adenosine **S4** (200 mg, 0.5 mmol, 1.00 equiv) was dissolved in DMA (10 mL) and cooled to 0 °C, followed by addition of sulfamoyl chloride (85 mg, 0.75 mmol, 1.5 equiv). The mixture was warmed to rt. After 12 h, sat. aq $NaHCO_3$ was added and the resulting mixture was extracted with EtOAc, washed with brine, dried (Na_2SO_4), and concentrated by rotary evaporation. Purification by silica flash chromatography (75% EtOAc in hexane) to *give the title compound* as a white powder (194 mg, 80%). Analytical data agreed with those reported previously (Ishikawa and Kakeya, 2014).

(*S*)-2-Amino-*N*1-(*N*-(((2*R*,3*S*,4*R*,5*R*)-5-(6-amino-9*H*-purin-9-yl)-3,4-dihydroxytetrahydrofuran-2-yl)-methyl)sulfamoyl)succinamide (S6, Asn-AMS)

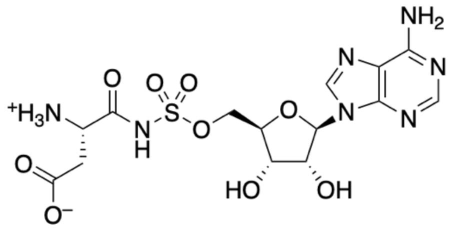

In a 10-mL round bottomed flask, protected AMS **S5** (80 mg, 0.016 mmol, 1.00 equiv) was dissolved in DMF (5 mL) and cooled to 0 °C, followed by addition of Boc-Asn(Trt)-OH (78 mg, 0.016 mmol, 1.00 equiv), HATU (62.5 mg, 0.016 mmol, 1.00 equiv) and DIPEA (86 µL, 0.048 mmol, 3.00 equiv). The reaction mixture was warmed to rt. After 12 h, the reaction mixture was extracted with EtOAc, washed with brine, dried (Na_2SO_4), and concentrated by rotary evaporation to afford the protected Asn-AMS intermediate. The crude product was dissolved in DCM (5 mL) at rt and TFA (1 mL) was added. After 5 h, the mixture was concentrated by rotary evaporation. Purification by reverse-phase HPLC (t_{ret} = 5.3 min) and lyophilization to *give the title compound* as a a clear colourless white solid (26 mg, 30% over two steps). HPLC (of 5–95% CH_3CN in 0.1% aq TFA): t_{ret} = 5.3 min, λ_{max} = 202, 240 nm; ^{1}H-NMR (600 MHz, methanol-d$_4$): δ 8.64 (s, 1H), 8.33 (s, 1H), 6.12 (d, *J* = 5.2 Hz, 1 H), 4.62 (t, *J* = 5.1 Hz, 1 H), 4.36 (m, 4 H), 3.96 (dd, *J* = 8.6, 3.6 Hz, 1 H), 2.97 (dd, *J* = 17.0, 3.6 Hz, 1 H), 2.79 (dd, *J* = 17.0, 8.6 Hz, 1 H); ^{13}C-NMR (151 MHz, methanol-d$_4$): δ 174.6, 174.2, 153.8, 150.3, 148.5, 142.9, 120.1, 89.8, 84.6, 76.5, 72.0, 69.1, 53.8, 35.6; MS (ESI) *m/z* calcd for $C_{14}H_{22}N_9O_7S$ ([M+H]$^+$) 460.95; found 460.97

References

Abdelsamie, A.S., C.J. van Koppen, E. Bey, M. Salah, C. Börger, L. Siebenbürger, M.W. Laschke, M.D. Menger, and M. Frotscher. 2017. Treatment of estrogen-dependent diseases: Design, synthesis and profiling of a selective 17β-HSD1 inhibitor with sub-nanomolar IC(50) for a proof-of-principle study. *European journal of medicinal chemistry*. 127:944-957.

Adams, P.D., P.V. Afonine, G. Bunkoczi, V.B. Chen, I.W. Davis, N. Echols, J.J. Headd, L.W. Hung, G.J. Kapral, R.W. Grosse-Kunstleve, A.J. McCoy, N.W. Moriarty, R. Oeffner, R.J. Read, D.C. Richardson, J.S. Richardson, T.C. Terwilliger, and P.H. Zwart. 2010. PHENIX: a comprehensive Python-based system for macromolecular structure solution. *Acta Crystallogr D*. 66:213-221.

Adjalley, S., and M.C.S. Lee. 2022. CRISPR/Cas9 editing of the *Plasmodium falciparum* genome. *Methods Mol Biol*. 2470:221-239.

Aragão, D., J. Aishima, H. Cherukuvada, R. Clarken, M. Clift, N.P. Cowieson, D.J. Ericsson, C.L. Gee, S. Macedo, N. Mudie, S. Panjikar, J.R. Price, A. Riboldi-Tunnicliffe, R. Rostan, R. Williamson, and T.T. Caradoc-Davies. 2018. MX2: a high-flux undulator microfocus beamline serving both the chemical and macromolecular crystallography communities at the Australian Synchrotron. *Journal of Synchrotron Radiation*. 25:885-891.

Burchat, A.F., J.M. Chong, and N. Nielsen. 1997. Titration of alkyllithiums with a simple reagent to a blue endpoint. *Journal of Organometallic Chemistry*. 542:281-283.

Cabrita, L.D., D. Gilis, A.L. Robertson, Y. Dehouck, M. Rooman, and S.P. Bottomley. 2007. Enhancing the stability and solubility of TEV protease using in silico design. *Protein science*. 16:2360-2367.

Cai, S.X.D., John A.;Kemnitzer, William E.;Sirisoma, Nilantha Sudath. 2007. N-arylalkyl-thienopyrimidin-4-amines and analogs as activators of caspases and inducers of apoptosis and the use thereof. Vol. US2007/99941. I.P. INC, editor. Cytovia, Inc., US.

Cingolani, P., A. Platts, L. Wang le, M. Coon, T. Nguyen, L. Wang, S.J. Land, X. Lu, and D.M. Ruden. 2012. A program for annotating and predicting the effects of single nucleotide polymorphisms, SnpEff: SNPs in the genome of Drosophila melanogaster strain w1118; iso-2; iso-3. *Fly*. 6:80-92.

De Schutter, J.W., J. Park, C.Y. Leung, P. Gormley, Y.-S. Lin, Z. Hu, A.M. Berghuis, J. Poirier, and Y.S. Tsantrizos. 2014. Multistage screening reveals chameleon ligands of the human farnesyl pyrophosphate synthase: Implications to srug discovery for neurodegenerative diseases. *Journal of medicinal chemistry*. 57:5764-5776.

Deitsch, K., C. Driskill, and T. Wellems. 2001. Transformation of malaria parasites by the spontaneous uptake and expression of DNA from human erythrocytes. *Nucleic Acids Res*. 29:850-853.

Dogovski, C., S.C. Xie, G. Burgio, J. Bridgford, S. Mok, J.M. McCaw, K. Chotivanich, S. Kenny, N. Gnadig, J. Straimer, Z. Bozdech, D.A. Fidock, J.A. Simpson, A.M. Dondorp, S. Foote, N. Klonis, and L. Tilley. 2015. Targeting the cell stress response of *Plasmodium falciparum* to overcome artemisinin resistance. *PLoS Biol*. 13:e1002132.

Duffey, M., B. Blasco, J.N. Burrows, T.N.C. Wells, D.A. Fidock, and D. Leroy. 2021. Assessing risks of *Plasmodium falciparum* resistance to select next-generation antimalarials. *Trends in Parasitology*. 37:709-721.

Eiler, S., A. Dock-Bregeon, L. Moulinier, J.C. Thierry, and D. Moras. 1999. Synthesis of aspartyl-tRNA(Asp) in *Escherichia coli* - a snapshot of the second step. *Embo J*. 18:6532-6541.

Emsley, P., B. Lohkamp, W.G. Scott, and K. Cowtan. 2010. Features and development of Coot. *Acta Crystallographica Section D-Biological Crystallography*. 66:486-501.

Evans, P.R. 2011. An introduction to data reduction: space-group determination, scaling and intensity statistics. *Acta Crystallogr D*. 67:282-292.

Evans, P.R., and G.N. Murshudov. 2013. How good are my data and what is the resolution? *Acta Crystallogr D*. 69:1204-1214.

Gamo, F.J., L.M. Sanz, J. Vidal, C. de Cozar, E. Alvarez, J.L. Lavandera, D.E. Vanderwall, D.V. Green, V. Kumar, S. Hasan, J.R. Brown, C.E. Peishoff, L.R. Cardon, and J.F. Garcia-Bustos. 2010. Thousands of chemical starting points for antimalarial lead identification. *Nature*. 465:305-310.

Ganesan, S.M., A. Falla, S.J. Goldfless, A.S. Nasamu, and J.C. Niles. 2016. Synthetic RNA-protein modules integrated with native translation mechanisms to control gene expression in malaria parasites. *Nat Commun*. 7:10727.

Ham, Y.J., D.-H. Lee, H.G. Choi, J.-M. Hah, and T. Sim. 2010. The efficient one-step chlorination of methylsulfanyl group on pyrimidine ring system with sulfuryl chloride. *Tetrahedron Letters*. 51:4609-4611.

Ishikawa, F., and H. Kakeya. 2014. Specific enrichment of nonribosomal peptide synthetase module by an affinity probe for adenylation domains. *Bioorg Med Chem Lett*. 24:865-869.

Kabsch, W. 2010. XDS. *Acta Crystallogr D*. 66:125-132.

Lawrence, G., Q.Q. Cheng, C. Reed, D. Taylor, A. Stowers, N. Cloonan, C. Rzepczyk, A. Smillie, K. Anderson, D. Pombo, A. Allworth, D. Eisen, R. Anders, and A. Saul. 2000. Effect of vaccination with 3 recombinant asexual-stage malaria antigens on initial growth rates of *Plasmodium falciparum* in non-immune volunteers. *Vaccine*. 18:1925-1931.

Liang, R., S. Li, R. Wang, L. Lu, and F. Li. 2017. N-Methylation of amines with methanol catalyzed by a Cp*Ir complex bearing a functional 2,2'-bibenzimidazole ligand. *Org Lett*. 19:5790-5793.

Manary, M.J., S.S. Singhakul, E.L. Flannery, S.E. Bopp, V.C. Corey, A.T. Bright, C.W. McNamara, J.R. Walker, and E.A. Winzeler. 2014. Identification of pathogen genomic variants through an integrated pipeline. *BMC bioinformatics*. 15:63.

Mccoy, A.J., R.W. Grosse-Kunstleve, P.D. Adams, M.D. Winn, L.C. Storoni, and R.J. Read. 2007. Phaser crystallographic software. *Journal of Applied Crystallography*. 40:658-674.

McKenna, A., M. Hanna, E. Banks, A. Sivachenko, K. Cibulskis, A. Kernytsky, K. Garimella, D. Altshuler, S. Gabriel, M. Daly, and M.A. DePristo. 2010. The Genome Analysis Toolkit: a MapReduce framework for analyzing next-generation DNA sequencing data. *Genome Res*. 20:1297-1303.

Miles, A., Z. Iqbal, P. Vauterin, R. Pearson, S. Campino, M. Theron, K. Gould, D. Mead, E. Drury, J. O'Brien, V. Ruano Rubio, B. MacInnis, J. Mwangi, U. Samarakoon, L. Ranford-Cartwright, M. Ferdig, K. Hayton, X.Z. Su, T. Wellems, J. Rayner, G. McVean, and D. Kwiatkowski. 2016. Indels, structural variation, and recombination drive genomic diversity in *Plasmodium falciparum*. *Genome Res*. 26:1288-1299.

Moriarty, N.W., R.W. Grosse-Kunstleve, and P.D. Adams. 2009. Electronic ligand builder and optimization workbench (eLBOW): A tool for ligand coordinate and restraint generation. *Acta Crystallographica Section D: Biological Crystallography*. 65:1074-1080.

Murithi, J.M., C. Pascal, J. Bath, X. Boulenc, N.F. Gnädig, C.F.A. Pasaje, K. Rubiano, T. Yeo, S. Mok, S. Klieber, P. Desert, M.B. Jiménez-Díaz, J. Marfurt, M. Rouillier, M.H. Cherkaoui-Rbati, N. Gobeau, S. Wittlin, A.C. Uhlemann, R.N. Price, G. Wirjanata, R. Noviyanti, P. Tumwebaze, R.A. Cooper, P.J. Rosenthal, L.M. Sanz, F.J. Gamo, J. Joseph, S. Singh, S. Bashyam, J.M. Augereau, E. Giraud, T. Bozec, T. Vermat, G. Tuffal, J.M. Guillon, J. Menegotto, L. Sallé, G. Louit, M.J. Cabanis, M.F. Nicolas, M. Doubovetzky, R. Merino, N. Bessila, I. Angulo-Barturen, D. Baud, L. Bebrevska, F. Escudié, J.C. Niles, B. Blasco, S. Campbell, G. Courtemanche, L. Fraisse, A. Pellet, D.A. Fidock, and D. Leroy. 2021. The antimalarial MMV688533 provides potential for single-dose cures with a high barrier to *Plasmodium falciparum* parasite resistance. *Science translational medicine*. 13.

Nasamu, A.S., A. Falla, C.F.A. Pasaje, B.A. Wall, J.C. Wagner, S.M. Ganesan, S.J. Goldfless, and J.C. Niles. 2021. An integrated platform for genome engineering and gene expression perturbation in *Plasmodium falciparum*. *Sci Rep*. 11:342.

Ni, Y., A. Gopalsamy, D. Cole, Y. Hu, R. Denny, M. Ipek, J. Liu, J. Lee, J.P. Hall, M. Luong, J.B. Telliez, and L.L. Lin. 2011. Identification and SAR of a new series of thieno[3,2-d]pyrimidines as Tpl2 kinase inhibitors. *Bioorg Med Chem Lett*. 21:5952-5956.

Park, J.S., M.C. Park, K.Y. Lee, P.C. Goughnour, S.J. Jeong, H.S. Kim, H.J. Kim, B.J. Lee, S. Kim, and B.W. Han. 2018. Unique N-terminal extension domain of human asparaginyl-tRNA synthetase elicits CCR3-mediated chemokine activity. *Int J Biol Macromol*. 120:835-845.

Phillips, M.A., J. Lotharius, K. Marsh, J. White, A. Dayan, K.L. White, J.W. Njoroge, F. El Mazouni, Y. Lao, S. Kokkonda, D.R. Tomchick, X. Deng, T. Laird, S.N. Bhatia, S. March, C.L. Ng, D.A. Fidock, S. Wittlin, M. Lafuente-Monasterio, F.J. Benito, L.M. Alonso, M.S. Martinez, M.B. Jimenez-Diaz, S.F. Bazaga, I. Angulo-Barturen, J.N. Haselden, J. Louttit, Y. Cui, A. Sridhar, A.M. Zeeman, C. Kocken, R. Sauerwein, K. Dechering, V.M. Avery, S. Duffy, M. Delves, R. Sinden, A. Ruecker, K.S. Wickham, R. Rochford, J. Gahagen, L. Iyer, E. Riccio, J. Mirsalis, I. Bathhurst, T. Rueckle, X. Ding, B. Campo, D. Leroy, M.J. Rogers, P.K. Rathod, J.N. Burrows, and S.A. Charman. 2015. A long-duration dihydroorotate dehydrogenase inhibitor (DSM265) for prevention and treatment of malaria. *Science translational medicine*. 7:296ra111.

Schmitt, E., L. Moulinier, S. Fujiwara, T. Imanaka, J.C. Thierry, and D. Moras. 1998. Crystal structure of aspartyl-tRNA synthetase from *Pyrococcus kodakaraensis* KOD: archaeon specificity and catalytic mechanism of adenylate formation. *Embo J*. 17:5227-5237.

Schrödinger, LLC. 2022. The PyMOL Molecular Graphics System, Version 2.5.

Schuck, P., and P. Rossmanith. 2000. Determination of the sedimentation coefficient distribution by least-squares boundary modeling. *Biopolymers*. 54:328-341.

Song, Y.-H. 2007. A facile synthesis of new 4-(phenylamino)thieno[3,2-d]pyrimidines using 3-aminothiophene-2-carboxamide. *Heterocyclic Communications*. 13:33-34.

Straimer, J., N.F. Gnadig, B. Witkowski, C. Amaratunga, V. Duru, A.P. Ramadani, M. Dacheux, N. Khim, L. Zhang, S. Lam, P.D. Gregory, F.D. Urnov, O. Mercereau-Puijalon, F. Benoit-Vical, R.M. Fairhurst, D. Menard, and D.A. Fidock. 2015. Drug resistance. K13-propeller mutations confer artemisinin resistance in *Plasmodium falciparum* clinical isolates. *Science*. 347:428-431.

Summers, R.L., C.F.A. Pasaje, J.P. Pisco, J. Striepen, M.R. Luth, K. Kumpornsin, E.F. Carpenter, J.T. Munro, D. Lin, A. Plater, A.S. Punekar, A.M. Shepherd, S.M. Shepherd, M. Vanaerschot, J.M. Murithi, K. Rubiano, A. Akidil, S. Ottilie, N. Mittal, A.H. Dilmore, M. Won, R.E.K. Mandt, K. McGowen, E. Owen, C. Walpole, M. Llinás, M.C.S. Lee, E.A. Winzeler, D.A. Fidock, I.H. Gilbert, D.F. Wirth, J.C. Niles, B. Baragaña, and A.K. Lukens. 2022. Chemogenomics identifies acetyl-coenzyme A synthetase as a target for malaria treatment and prevention. *Cell chemical biology*. 29:191-201.e198.

Swann, J., V. Corey, C.A. Scherer, N. Kato, E. Comer, M. Maetani, Y. Antonova-Koch, C. Reimer, K. Gagaring, M. Ibanez, D. Plouffe, A.-M. Zeeman, C.H.M. Kocken, C.W. McNamara, S.L. Schreiber, B. Campo, E.A. Winzeler, and S. Meister. 2016. High-throughput luciferase-based assay for the discovery of therapeutics that prevent malaria. *ACS infectious diseases*. 2:281-293.

Wang, J., M. Su, T. Li, A. Gao, W. Yang, L. Sheng, Y. Zang, J. Li, and H. Liu. 2017. Design, synthesis and biological evaluation of thienopyrimidine hydroxamic acid based derivatives as structurally novel histone deacetylase (HDAC) inhibitors. *European journal of medicinal chemistry*. 128:293-299.

Winn, M.D., C.C. Ballard, K.D. Cowtan, E.J. Dodson, P. Emsley, P.R. Evans, R.M. Keegan, E.B. Krissinel, A.G.W. Leslie, A. McCoy, S.J. McNicholas, G.N. Murshudov, N.S. Pannu, E.A. Potterton, H.R. Powell, R.J. Read, A. Vagin, and K.S. Wilson. 2011. Overview of the CCP4 suite and current developments. *Acta Crystallogr D*. 67:235-242.

Woodring, J.L., G. Patel, J. Erath, R. Behera, P.J. Lee, S.E. Leed, A. Rodriguez, R.J. Sciotti, K. Mensa-Wilmot, and M.P. Pollastri. 2015. Evaluation of aromatic 6-substituted thienopyrimidines as scaffolds against parasites that cause trypanosomiasis, leishmaniasis, and malaria. *MedChemComm*. 6:339-346.

Xie, S.C., C. Dogovski, S. Kenny, L. Tilley, and N. Klonis. 2014. Optimal assay design for determining the in vitro sensitivity of ring stage *Plasmodium falciparum* to artemisinins. *Int J Parasitol*. 44:893-899.

Zhang, C., C. Tan, X. Zu, X. Zhai, F. Liu, B. Chu, X. Ma, Y. Chen, P. Gong, and Y. Jiang. 2011. Exploration of (S)-3-aminopyrrolidine as a potentially interesting scaffold for discovery of novel Abl and PI3K dual inhibitors. *European Journal of Medicinal Chemistry*. 46:1404-1414.
